# Identification of *RAN1* orthologue associated with sex determination through whole genome sequencing analysis in fig (*Ficus carica* L.)

**DOI:** 10.1038/srep41124

**Published:** 2017-01-25

**Authors:** Kazuki Mori, Kenta Shirasawa, Hitoshi Nogata, Chiharu Hirata, Kosuke Tashiro, Tsuyoshi Habu, Sangwan Kim, Shuichi Himeno, Satoru Kuhara, Hidetoshi Ikegami

**Affiliations:** 1 Faculty of Agriculture, Kyushu University, Fukuoka, Japan; 2 Kazusa DNA Research Institute, Kisarazu, Chiba, Japan; 3 Fukuoka Agriculture and Forestry Research Center Buzen Branch, Yukuhashi, Fukuoka, Japan; 4 Fukuoka Keichiku Agricultural Extension Center, Fukuoka, Japan; 5 Fukuoka Agriculture and Forestry Research Center, Fukuoka, Japan; 6 Graduate School of Agriculture, Ehime University, Ehime, Japan

## Abstract

With the aim of identifying sex determinants of fig, we generated the first draft genome sequence of fig and conducted the subsequent analyses. Linkage analysis with a high-density genetic map established by a restriction-site associated sequencing technique, and genome-wide association study followed by whole-genome resequencing analysis identified two missense mutations in *RESPONSIVE-TO-ANTAGONIST1 (RAN1*) orthologue encoding copper-transporting ATPase completely associated with sex phenotypes of investigated figs. This result suggests that *RAN1* is a possible sex determinant candidate in the fig genome. The genomic resources and genetic findings obtained in this study can contribute to general understanding of *Ficus* species and provide an insight into fig’s and plant’s sex determination system.

The fig (*Ficus carica* L.; Moraceae; 2*n* = 2*x* = 26)^1^ has been an important food source throughout human history. The species, which has been cultivated for over 11,000 years, is considered to be the oldest cultivated crop^2^,^3^. In 2013, the world’s total production of fig fruit was estimated to be 1.1 million metric tons^4^, mainly from Mediterranean countries^5^. In addition to serving as a food source over many centuries, members of the genus *Ficus* are also one of the earliest and best sources of cultivated medicine^6^.

Fig plants possess several unique characteristics, such as the presence of syconium, fruit-bearing^7^, caprification, and sex differences. Sexual system are essential to species survival in plants including fig and one of the most important subject in reproductive biology, and reproductive biology on sex differences is rather complex. Fig is a diclinous plant, with individuals generally represented by two sexual forms: the caprifig and the fig^2^. The caprifig is ambisexual tree with staminate flowers and short-style pistillate flowers (Monoecious; functionally male fig), whereas the fig is unisexual tree possessing only long-style pistillate flowers (Gynoecious; female fig: i.e., Smyrna, San pedro, and common-type figs). (Fig. 1A and B) The caprifig (‘male’) is presumed to be wild and ancestor form of the fig (‘female’)^8^. On their functional grounds, it is equally acceptable to label this species as either gynodioecious or dioecious (or subdioecious)^8^. Fig has the XY chromosome-based sex-determination system^2^ but seems to have homomorphic chromosomes because sex chromosomes are indistinguishable cytologically^9^. Data from crossing experiments suggest that sex determination in fig is controlled by a single locus being responsible for the presence or absence of stamens, i.e. *A* locus, closely linking to *G* locus controlling the length of pistils^2^ (Fig. 1C and D). However, genes and molecular mechanisms conferring the sex determination have not been identified because of lacks of genomics information on *Ficus* species including *F. carica*.

To identify sex determinants in fig, genetic analyses, e.g., quantitative trait locus (QTL) mapping and genome wide association study (GWAS), would be necessary and a straightforward strategy. In the present study, we thereby organized the first draft genome of fig and examined its overall sequence characteristics. We subsequently conducted a restriction site-associated DNA sequencing (RAD-seq) analysis of an F_1_ population to construct the first-ever high-density linkage map on the basis of genome-wide single nucleotide polymorphisms (SNPs). Using the genomics resources for the genetic analyses, we searched a series of molecular databases for staminate flower forming gene putatively involved in sex determination of fig individuals. The result led to the discovery of a prime candidate gene *RESPONSIVE-TO-ANTAGONIST1 (RAN1*). The genomic resources and genetic findings obtained in this study not only contribute to general understanding and improvement of fig species but also provide an insight into plant’s sex determination system.

## Results

### Sequencing and genome assembly

We used ‘Horaishi’, the most traditional fig cultivar in Japan, as experimental material and sequenced its genome using the Illumina platform in conjunction with the shotgun method. A total of 34,876,515,042 bp of sequence information was obtained by next-generation sequencing using three platforms: Illumina GAIIx, MiSeq and HiSeq 2000 (Supplementary Table S1). *K*-mer analysis revealed that two large peaks were present with considerable heterogeneity (Supplementary Figure S1). The size of the fig genome was estimated to be 259–393 Mb, consistent with the 356-Mb level estimated by flow cytometry^10^.

In the assembly of 184 million paired-end reads of sequencing data generated using the three Illumina sequencers, 154.6 million reads (84.0%) could be assembled into 2.8 million contigs, with a total length of 466 Mb. The largest contig length and N50 value were 10,794 and 241 bases, respectively. Subsequent scaffold construction generated 478,193 scaffolds including sequences above 100 bases and the total length was 314 Mb. After closing gaps represented by ‘N’ bases in the scaffolds with Illumina reads, the total length of final scaffolds with more than 500 bases was 248 Mb, with a GC% of 33.4. We finally obtained a draft genome sequence of fig consisting of 27,995 scaffolds, in which the largest scaffold size and N50 were 1.7 Mb and 166 kb, respectively (Table 1). The draft genome sequence covered approximately 70% of the fig genome size (356 Mb; Table 1). On the other hand, BUSCO data set assessment of the genome assembly indicated a composition of 90% complete single copy, 5.1% fragmented and 4.6% missing (Supplementary Table S2). Since the draft genome sequence would cover large parts of gene-rich regions in the fig genome, we concluded that the genome sequence data would be sufficient to discover genes for sex determination in fig.

### Genome annotation and characterization

A total of 36,138 protein-coding genes (total length of 32,823,112 bases with a GC% of 47.4) were predicted in the genome assembly (Table 1 and Supplementary_Data_1 and 2). Average and N50 lengths of the predicted genes were 908.3 and 1,383 bases, respectively. Among them, 22,250, 20,679 and 17,432 predicted genes could be annotated by BLAST nr, InterPro and Gene Ontology (GO) databases was, respectively. In total, 69.2% of the predicted genes were functionally annotated (Supplementary Table S3). Repetitive sequences comprised 20.9% of the fig genome. Retroelements (4,580) were approximately four times as frequent as DNA transposons (1,174), with unclassified transposons constituting the largest group (14.9%) of repetitive sequences. The most common retroelements were LTR elements, which were represented by similar proportions of *Ty1/Copia* and *Gypsy/DIRS1* (Supplementary Table 4). With respect to the Kyoto Encyclopedia of Genes and Genomes (KEGG) pathway distributions, signal transduction pathway genes (class III) (780) followed by carbohydrate metabolism pathway genes (class I) (765) were the most common. The signalling molecule and interaction pathway was represented by the lowest gene count (3) (Supplementary Figure S2).

### Expressing gene profiles in each organs

To further understand the characteristics of the fig genome, we mapped transcriptome data from three kinds of organs (fruit, leaf and stem) to the assembled genome and obtained their expression profiles. Of the 17,355 genes that were mapped to the fig genome, which was roughly half the number of predicted genes (48.0%), 8,292 were expressed in all organs. In addition, 1,069 (fruit), 2,337 (leaf) and 1,377 (stem) genes were identified as showing organ-specific expression (Supplementary Figure S3).

### Construction of a high-density genetic linkage map

**Figure 2:**
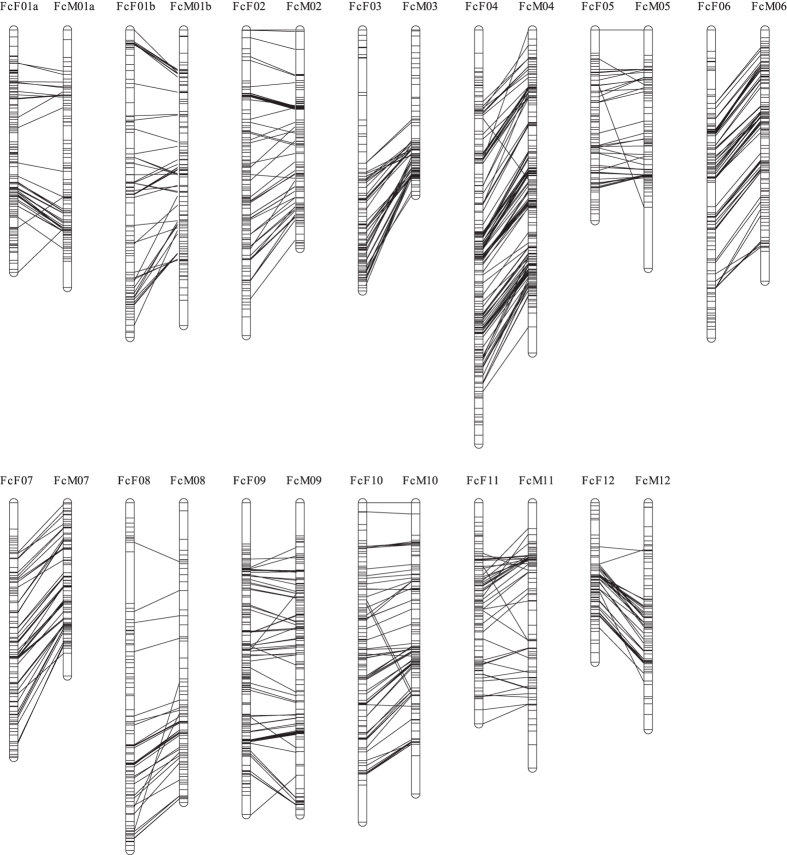
Comparison of genetic maps from Caprifig (male) and Fig (female). Total 7498 SNP markers were mapped to the 13 linkage groups corresponding to 13 chromosomes. Most of marker positons were conserved between Caprifig and Fig maps.

To anchor the genome sequence to the fig chromosomes, we developed a high-density genetic map with two molecular marker systems. Because few DNA markers have been reported in fig, we initially designed 3,648 simple sequence repeat (SSR) markers in this and a previous study^11^ using transcript sequences from fig fruit, leaf and stem (Supplementary_Data_3). After 480 of 3,467 designed markers were tested, 79 were selected as polymorphic markers between male (Caprifig6085) and female (Horaishi) figs as parental lines of an F1 mapping population. Next, we also carried out RAD-Seq on the 52 F1 individuals as well as the parental lines (Supplementary Tables S5 and S6). Approximately 5.3 million reads per line were obtained and 69% of them were aligned onto the draft genome sequence to detect SNP candidates. After filtering with criteria described in Materials and methods section, 8,664 high-quality SNPs were selected for the following linkage analysis. As results, 64 SSRs and 8,180 SNPs that segregated in the mapping population were grouped into 13 groups each for paternal and maternal genome. After linkage analysis, we obtained two genetic maps composed of 13 linkage groups encompassing 1,063.2 cM for the paternal genome and 13 linkage groups covering 1,167.4 cM for the maternal genome (Fig. 2). A one-to-one correspondence was observed between the female and male linkage groups by connecting identical marker loci between the two parents. Subsequently, the two genetic maps were integrated into a high-density consensus linkage map (total linkage map length: 1,024.1 cM; average map distance between two neighbouring loci: 0.14 cM) bearing 7,498 markers (58 SSRs and 7,440 SNPs) (Supplementary Figure S4, Supplementary Table S7, Supplementary_Data_4). The fig linkage groups were numbered in accordance with the names of jujube (*Ziziphus jujuba*) chromosomes reported by Liu *et al*.^12^ (see below for details).

### Genomic organization comparisons with other species

**Figure 3:**
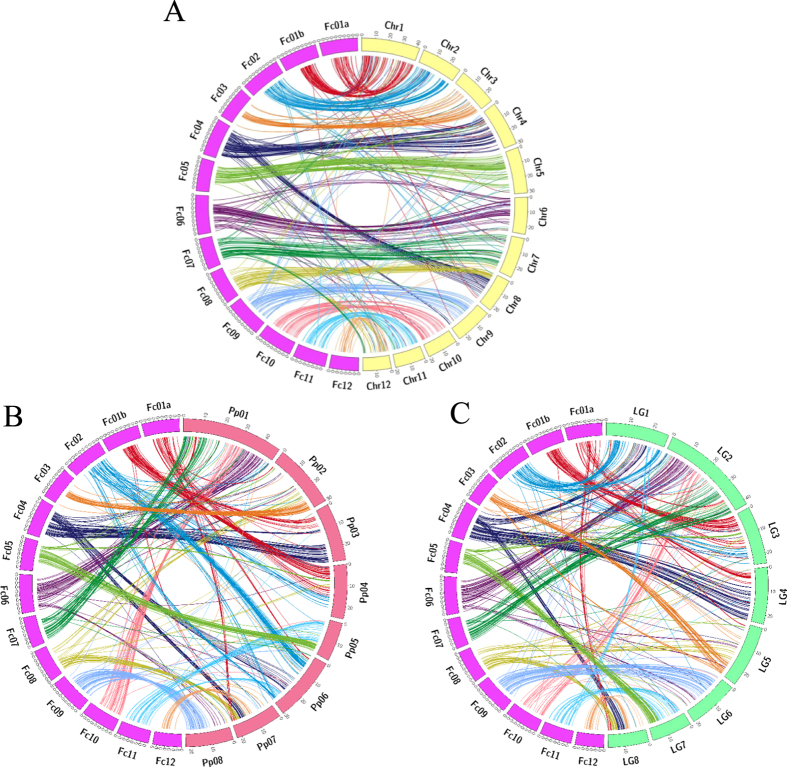
Synthetic relationships between fig and other fruit tree species. (**A**) pseudo chromosome comparison between fig and jujube (*Ziziphus jujube*). one to one relationship of pseudo-chromosomes was confirmed except for Fc01. Fig pseudo-chromosome which correspond to jujube Chr1 is divided into two pseudo-chromosomes Fc01a and Fc01b. Fig chromosome number was determined by reference to correspondent jujube chromosome number. (**B**) pseudo chromosome comparison between fig and peach (*Prunus persica*). (**C**) pseudo chromosome comparison between fig and Japanese apricot (*Prunus mume*).

To understand the structural features of the fig genome at the chromosome level, we carried out a similarity search of the genome sequences covering SNP loci on the fig genetic map vs. the pseudo-molecules of peach (*Prunus persica*)^13^, mume (*Prunus mume*)^14^ and jujube^12^, all of which are Rosales and are close to Moraceae including *F. carica*^15^. Of the 7,440 SNPs on the fig genetic map, 798, 750 and 887 showed significant sequence similarities (E-value < 1 × 10^−15^) to the genome sequences of peach, mume and jujube, respectively. Orders of the marker loci on the fig map were basically conserved in the genomes of peach, mume and jujube, indicating the existence of clear syntenic relationships on many of the chromosomes (Fig. 3). In particular, we note that while most of the fig linkage groups were observed to have a strong syntenic relationship to the jujube genome with a roughly one-to-one correspondence, two probable chromosome rearrangements were predicted: Fc01a and Fc01b corresponding to chromosome 1 of jujube and part of Fc04 and all of Fc08 matching chromosome 8 of jujube.

### Genetic diversity of fig cultivars

To assess fig genetic diversity, a whole-genome resequencing analysis was performed on five divergent fig cultivars consisting of two male and three female lines. Approximately 20–90 × depth of sequence read coverages were obtained. An average of 67% of the reads were mapped onto the draft genome sequence. After eliminating low-quality data by filtering with criteria mentioned in Materials and methods section, a total of 3,822,503 high-quality polymorphic loci including 3,144,140 SNPs and 678,363 indels were detected from the mapping alignment. The SNPs comprised 1,893,302 transition and 1,250,838 transversion variations (Ts/Tv = 1.51). Among the SNPs and indels, 70.4% were located in intergenic regions followed by 21.3% in introns and 7.7% in exons. In accordance with the Sequence Ontology terms^16^ in SnpEff software^17^, predicted functional impacts on genes in the draft genome sequence were categorized into four classes, high (0.5%), moderate (5.3%), modifier (89.9%) and low (4.4%).

### Genes involved in sex determination

**Figure 4:**
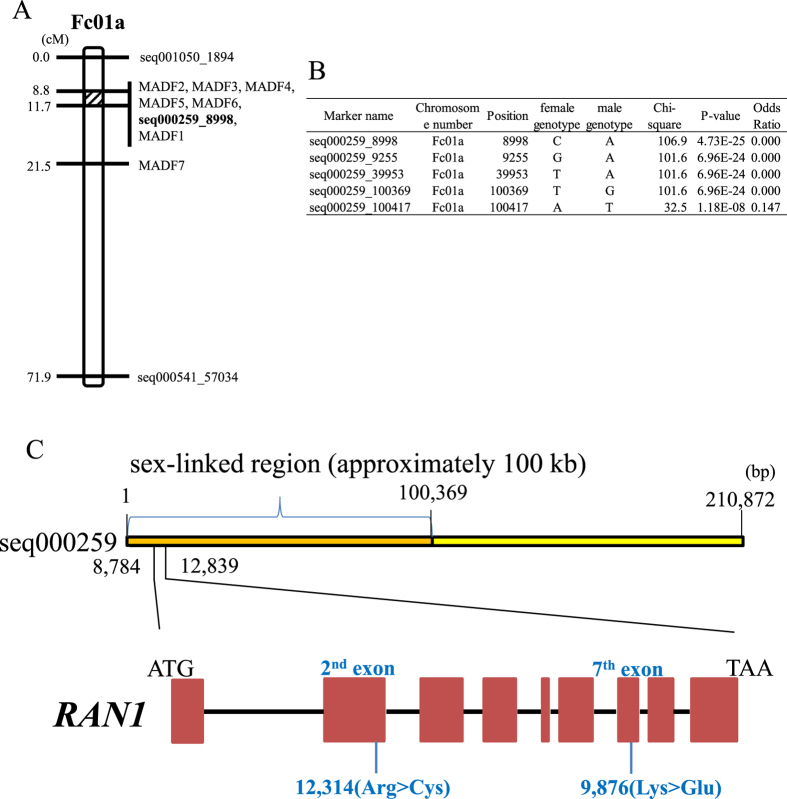
Search of sex determination *A* gene. (**A**) MADF (Male DNA Associated Sequence) markers and seq000259_8998 marker positioned at the diagonal region of Fc01a chromosome. (**B**) Top 5 GWAS detecting SNP markers. Most statistically significant SNP markers were mapped to the scaffold seq000259 on the Fc01a chromosome. seq000259_8998 marker genotypes completely matched the sex phenotypes of 119 test materials. (**C**) RAN1 gene structure, sex controlling and sex evolution model in fig. RAN1 is composed of 9 exons and 8 introns. Each of 2nd exon and 7th exon has one missense variations at 12,314th and 9,876th positon respectively.

A linkage analysis using the genetic map generated in this study should be an effective initial approach for identifying the locus position of the sex-determination gene. Because seven random amplified polymorphic DNA-sequence characterized amplified region (RAPD-SCAR) markers that have been developed as linked to the male phenotype (AB331722–AB331728; Supplementary Table S8) are avaliable, we investigated map positions of the seven RAPD-SCAR markers. Six of the markers were found to be located between positions 8.8 to 11.7 cM of the Fc01a linkage group, while the remaining one was on 21.5 cM of the Fc01a. This result indicated that the sex-determining *A* gene is likely located near the 8.8–11.7 cM region on the Fc01a linkage group (Fig. 4A, Supplementary_Data_4).

To further refine the location of the causative gene, we performed a GWAS on 122 genotypes, out of which sex phenotypes of 119 were known (Supplementary Tables S9 and S10). By mapping RAD-seq sequences from the 122 genotypes to the draft genome, we were able to detect 16,124 SNPs (Supplementary_Data_5). Out of them, 95 were significantly associated with sex phenotype (Bonferroni-corrected *p* < 3.1 × 10^−6^), and 72 of them were considered to be linked to the 2.4–31.4 cM region on the Fc01a linkage group (Fig. 4A). In paticular, the top 4 SNPs of ranking were highly (99.2–100.0%) associated with all the known sex phenotypes of 119 individuals, including one (seq000259_8998) being completely correlated with the sex phenotypes (Fig. 4B). Because the four SNPs with high significance were concentrated in an approximately 100-kb region of scaffold seq000259 at 9.2 cM (Supplementary Table S11), we considered the *A* gene is located in this scaffold. Therefore, we investigated all mutation sites within this region using the whole-genome resequencing data of the five fig cultivars and the sequenced draft genome (a total of two males and four females). While 889 SNPs (corresponding to 11 genes) were identified in this region, 39 SNPs having probable critical effects (high or moderate) on gene functions were correlated with sex phenotypes in the five cultivars and Horaishi (Table 2). To specify the causative SNP(s) and gene(s), we genotyped the 39 SNPs across additional 18 male lines. Genotypes of the *A* locus in the 18 male lines were expected to be heterozygous. As a result, only two SNP sites (seq000259_9876, seq000259_12314) with moderate impacts (missense mutations), both of which located on a single gene annotated as *RAN1*, showed heterozygous genotypes in all 18 males including “Palmata” (*F. palmata*) that is distantly related to other varieties (Supplementary Tables S12 and S13). Furthermore, genoytpes of the two SNPs were completely match with the phenotypes of the 119 individuals (Supplementary_Data_7). The results suggest that *RAN1* is a prime candidate for the sex determining *A* gene in fig.

To clarify the precise transcription of *RAN1*, we sequenced *RAN1* transcripts and checked its expression using RNA-seq data. *RAN1* cDNA sequence was total 3,015 bp and proved that there were no difference in splicing pattern between male and female (Supplementary_Data_8). In addition, all cDNA SNP positions detected were completely consistent with SNP positions observed through whole genome resequencing analysis. Horaishi had two allelic sequences, a complete full length coding sequences and that having a single base deletion at 9,823th position causing frameshift mutations, the latter of which was represented in the genome assembly. The expression value (in RPKM) for *RAN1* in differential material was 18.2–50.3, indicating no clear evidence of sex and organ specificity in its expression (Supplementary Figure S5).

The two SNPs were missense mutations causing amino acid residue substitutions in the putative peptide sequences of RAN1 protein. The seq000259_9876 caused an amino acid substitution at the 724^th^ peptide position in 7^th^ exon from lysine (AAA in female) to glutamic acid (GAA in male). The other SNP of seq000259_12314 was a little complex because it was found at the position of 12,315^th^, which genotypes were not associated with the sex phenotype (Fig. 4C). A combination of the two SNPs at 12,314^th^ and 12,315^th^ positions in 2^nd^ exon generated three codons for the 278^th^ amino acid, e.g., CAT for histidine (male), CGT for arginine (female) and TGT for cysteine (female) (Supplementary_Data_7). A simple PCR screening method by CAPS marker was developed to easily distinguish the three alleles. The PCR products amplified from a single pair of primers for the CAT allele and the CGT were digested with *Pci*I and *Hpy*CH4IV, respectively, while those from the remaining TGT alleles were not fragmented. The resultant banding patterns of the CAPS analysis completely matched to the results from the sequence analysis, indicating that the sex phenotypes in the investigated fig individuals could be screened with the CAPS marker (Supplementary Figure S6).

## Discussion

In this study, we generated the first reported draft genome sequence of fig and the genus *Ficus*. In our genome assembly, the length of the largest scaffold (1.7 Mb) and N50 value (166 kb) were almost similar to those reported for *Morus notabilis* (mulberry) (3.5 Mb and 39 kb, respectively)^18^, a species closely related to fig. The number of predicted genes was also comparable to those of mulberry (29,338)^18^. Indeed, the total length of the assembled genome (248 Mb) was approximately 30% shorter than the estimated size (356 Mb). Repetitive sequences constituted only 20.9% of the draft sequence of fig, a proportion much lower than that in the similar-sized genome of mulberry (357 Mb). Because the predicted genes (36,138) in the assembled draft sequence covered 96.2% of transcripts (>500 bp) obtained from fruit, leaf and stem and 90% of the BUSCO gene set in our previous study^11^, we suspected that the remaining unassembled genome fraction might be enriched in repetitive rather than coding sequences. Based on this assumption, the proportion of repetitive sequences in the fig genome was estimated to be 20.9–64.5%, which is consistent with the percentage in the mulberry genome (40.30%)^18^. We also successfully predicted a candidate gene for sex determination, which indicate that the current draft genome assembly should be sufficient for subsequent post-genome studies in fig.

Genetic maps for female and male parents as well as the integrated consensus map consisted of 13 linkage groups. The number of linkage groups inferred from these maps is identical to the haploid chromosome number^19^, which suggests that each linkage group represents a single chromosome. Because the order of mapped loci between male and female maps is basically consistent, the genome structure of male and female plants is totally conserved including sex chromosome, Fc01a. This result supports the chromosome observation by Storey^9^, which demonstrates that sex in fig is determined by a single locus on homomorphic sex chromosomes, rather than on heteromorphic sex chromosomes, e.g., the XY chromosomes^20^.

Interestingly, comparative chromosomal analysis revealed a high level of synteny between genome structures of fig and jujube^1^,^2^,^21^ (Fig. 3A), both of which are members of order Rosales, but belong to different families, namely, Moraceae and Rhamnaceae, respectively. A strong one-to-one correspondence was detected between jujube chromosomes and all chromosomes of fig except for Fc01a, Fc01b, Fc04 and Fc08, indicating the existence of a close evolutionary relationship of these species. This observation supported the molecular-based classification, Moraceae is more closely related to Rhamnaceae than to Rosaceae^22^.

Our exploration of the *A* gene responsible for formation of male flowers revealed that chromosome Fc01a is the location of this gene. In particular, *RAN1* orthologue on the 100-kb region of scaffold seq000259 most closely matches the true *A* gene for the following reasons. (1) a number of sex linked markers located near *RAN1* orthologue locus on Fc01a chromosome, (2) *RAN1* orthologue locus positioned in the probably sex linked region^23^ (100-kb region), (3) *RAN1* orthologue has the exclusive mutations that could account for the sex phenotypes of all genotypes including “Palmata” (*F. palmata*) possibly distantly related to other varieties (Fig. 4, Supplementary Table S13, Supplementary_Data_7), (4) *RAN1* is involved in the transduction of ethylene which was reported to control sex phyenotype in some species^24^,^25^.

Recently, regarding the genetic model for monoecy sex determination in plants, one model was proposed. In this model, differences in the development of the unisex flowers of melon and cucumber involve multiple genes related to ethylene synthesis^26^. Specifically, *ACS11* represses the carpel inhibitor *WIP1*, which in turn represses the stamen inhibitor *ACS7*. Because it allows *WIP1* to prevent directly carpel formation and indirectly stimulate stamens, the absence of *ACS11* results in exclusively male flowers^26^,^27^.

*RAN1*, which encodes copper-transporting ATPase, is involved in the first step of ethylene perception. *RAN1* delivers the copper cofactor to membrane-targeted ethylene receptor apoproteins. After the incorporation of a copper ion, the receptors are able to coordinate ethylene^28^. As a result of reduced copper transport in *RAN1*-cosuppressed plants (no copper), the metal-deficient ethylene receptors are non-functional, resulting in a constitutively activated pathway and in constitutive ethylene response plant phenotypes^29^. If ethylene action applicable to melon and cucumber also works in fig, *RAN1* with normal function can allow *WIP1* expression through reduced sensitivity of ethylene synthesized by *ACS11* or simply reduce sensitivity of ethylene synthesized by *ACS7*, which leads to stamen formation. In other words, a possible mechanism of stamen developmental control in fig involves ethylene receptors that are active because of the presence of normal *RAN1* in male plants harbouring *RAN1* hetero mutations (*RAN1/ran1*), with these receptors thus suppressing ethylene perception and inducing stamen formation (Supplementary Figures S7 and S8). Intriguingly, malfunction of Arabidopsis *ran1* mutants are caused by a single base changes in 2^nd^ and 4^th^ exon region^28^. In addition, *Ficus* and *Cucumis* have similar pathway to gender dimorphism^30^, which is reinforced by the confirmation of the presence of several *WIP* and *ACS* homologues in the fig genome sequence (e.g. g09010, g24002, g02797 etc.; Supplementary_Data_2). Although this hypothesis is not proven functionally, it is the strongest candidate currently (Table 2, Supplementary Table S13).

Charlesworth described that change from cosexuality (monoecy) to dioecy probably involves a mutation creating females (a mutation suppressing some or all female flowers in an initially monoecious species, or replacing them with male flowers), and then one or more female-suppressing mutations, creating males or male-biased plants^31^,^32^. If either or both of *RAN1* variations correspond with the former mutation, *RAN1* is responsible gene for creating females. We are currently analysing the function of the *RAN1* gene through transgenic experiments.

As for the dioecy sex determination, the *OGI*/*MeGI* gene system, has been reported in *Diospyros lotus*^33^. The model proposed to explain this system is that *OGI* encoding a small RNA and homologous to *MeGI*, which inhibits male growth, influences the function of *MeGI* in an oppressive manner through RNA silencing. Although fig is functionally dioecious and thus different from *D. lotus*, this model might be applicable to fig as well. Interestingly, the presence of *MeGI* was confirmed in the draft fig genome (not linked to the linkage map), whereas no *OGI* sequence homologous to the *MeGI* sequence was observed in the present sequences of female or male individuals.

Fig has many excellent properties such as a small genome size, a rapid transition from seedling to fruit bearing, the possibility of transformation, production of a large number of seeds and abundant genomic resources^1^,^5^,^34^ that may be highly heterozygous and polymorphic (Supplementary Figure S1, Supplementary_Data_5 and 6). Fig may therefore be a suitable species for genomic applications such as GWAS and genomic selection. Future applications of the fig genomic information uncovered in this study would contribute to an understanding of its useful and unique properties and to the improvement of its productivity and marketability.

## Materials and Methods

### Whole genome sequencing (WGS) analysis

Total genomic DNA that was extracted from leaves of the fig cultivar Horaishi using a DNeasy plant kit (Qiagen, Valencia, CA, USA) was subjected to library construction. Paired-end and mate-pair libraries with multiple insert sizes (500 to 5,000 bp) were constructed for Illumina sequencing according to the manufacturer’s instructions (Illumina, San Diego, CA, USA). Genomic DNA was fragmented, linked to adapters and size selected. We obtained 32.6 Gb of raw WGS data. Five rules were applied during processing of the raw data: (1) adapter removal, (2) removal of leading low-quality (quality score < 3) or ‘N’ bases, (3) removal of trailing low-quality or N bases, (4) scanning of reads (using a 4-base-wide sliding window) and removal when the average quality per base dropped below 15 and (5) removal of reads less than 36 bases long. After filtering, 18.6 Gb of high-quality data were retained, representing approximately 60× genome coverage. To find genome-wide SNPs and indel polymorphisms, Horaishi and five additional cultivars, namely, males Caprifig 6085 and Capri Type, and females Masui Dauphine, Toyomitsuhime and King, were used for the whole-genome resequencing analysis.

### *De novo* genome assembly

The fig genome was *de novo* assembled using a hierarchical assembly strategy along with a WGS approach. We assembled the WGS sequencing data using Platanus software (ver. 1.2.1)^35^ with the *K*-mer value set automatically. The procedure included the following three steps: (1) contig assembling (2) scaffold construction and (3) closing of gaps. Repetitive elements in the assembly were identified by the Repbase-based method^36^. As a first step, we built *de novo* repeat libraries using the RepeatScout^37^ program based on a *F. carica* model. Next, we searched for repeats by applying RepeatMasker^38^ with an *Arabidopsis thaliana* model to the assembly and then re-applying RepeatMasker with the *F. carica* model to the results.

### Gene prediction and annotation

We used *ab initio* and homology-based methods to predict genes in the *F. carica* genome. For *ab initio* gene prediction, we used the Augustus program (ver. 3.2.1)^39^. Gene model and exon-intron rules were acquired by the program via training using the known gene sequences from *Morus notabilis* and all RNA-seq reads from *F. carica* leaf aligned with TopHat. We then carried out homology-based gene prediction by mapping protein sequences from six plant species onto the *F. carica* genome using TBLASTN (*E*-value < 1 × 10^−5^) according to the method of Liu *et al*.^12^. Finally, we combined all data sets to generate a high confidence gene set.

We performed functional gene annotations by BLASTP, InterProScan and KEGG KAAS alignments to nr, InterPro and KEGG GENES databases, respectively. Gene ontology and KEGG orthology terms were obtained from the corresponding InterProScan and KEGG entries.

### RNA-seq and EST-SSR marker development

Sequences for EST-SSR primer design were collected from three sources: fruit (syconium)^11^, leaf and stem (in this study) transcriptome. RNA-Seq library construction and sequencing were performed as described in Ikegami *et al*.^11^. Total 3,648 pairs of primers were designed using Primer3^40^ according to Ueno *et al*.^41^ and 480 pairs were screened for the detectability of polymorphisms between the F1 parents by 10.0% PAGE (Poly-Acrylamide Gel Electrophoresis).

### RAD-seq for genetic map construction

RAD-Seq analysis was performed as described in Shirasawa *et al*.^42^. The RAD-Seq libraries for the F1 mapping population (*n* = 52), its parents Horaishi and Caprifig6085, and 122 genotypes (22 Males, 97 Females and 3 unknown sex individuals; Supplementary Table 9) were constructed using restriction enzymes *Pst*I and *Msp*I. Nucleotide sequences of the libraries were determined on a HiSeq (Illumina) system in paired-end, 93-bp mode. The sequence data was processed and mapped onto the genome assembly. SNPs called from the mapping alignments were used for linkage and association analysis.

The SSRs and SNPs segregated data of the mapping population were prepared for the CP mode of JoinMap4^43^ and classified into groups using the Grouping Module of JoinMap4 with logarithm of odds (LOD) scores of 4 to 7. The Combine Groups of Map Integration Module was used to integrate the parental linkage maps. Marker order and relative map distances were calculated using the regression-mapping algorithm with the following parameters: Haldane’s mapping function, recombination frequency ≤0.35 and LOD score ≥2.0. Graphical linkage maps were drawn using the MapChart program^44^.

For comparative genomics, similarity searches of marker-associated sequences against genome sequences of jujube^12^, peach^13^ and mume^14^ were carried out using BlastN^45^ with a cut-off value of 1 × 10^−15 ^33. Graphical comparative maps were drawn using the Circos program^46^.

### Association analysis

A GWAS of 122 genotypes was conducted using their corresponding genotype datasets derived from RAD-Seq. In each panel, only SNPs with a minor allele frequency >5% were used for the association analysis. A case-control association study treating the caprifig type as the control and the common fig type as the case was conducted using PLINK 1.07^47^. Sex phenotype data were collected at the Fukuoka Agriculture and Forestry Research Center, Buzen Station, Japan, between 1989 and 2015.

### Genome resequencing analysis and SNP annotation

A comparative analysis using genome sequences of Horaishi and five other fig cultivars was performed to validate the significant SNPs of the GWAS. Other than the data for Horaishi, we obtained resequencing data by HiSeq2000 sequencing. The sequence reads were trimmed and mapped onto the Horaishi reference genome assembly. SNP and indel candidates were predicted from the mapping alignments using thresholds of SNP quality value >10 and raw-read depth >5 as described in our previous study^42^. Categorization of SNP effects was performed using SnpEff v3.0^17^. The default parameters of SnpEff were used to perform the variant effect analysis.

### CAPS marker analysis

A pair of PCR primers, FigFM_f (5′-CAATACCAAAATGATATGCACGA-3′) and FigFM_r (5′-TGGCATATACAGTGAGATGGATG-3′), were designed on the flanking sequences of the SNP of seq000259_12314 to amplify 315-bp-length DNA fragments. Two restriction enzymes, *Pci*I (New England Biolabs, lpswich, MA, USA) and *Hpy*CH4IV (New England Biolabs) were used for digestion of the PCR products. CAPS analysis was performed as described previously^48^.

### cDNA sequencing and expression profiling

The complete *RAN1* coding regions of two sexes (Caprifig6085 and Horaishi) were amplified from fruit totalRNA using PrimeScript™ II High Fidelity One Step RT-PCR Kit (Takara Bio, Shiga, Japan). A pair of PCR primers, FcRAN1-F1 (5′-ATGGCGGCGAGCGTCCGACACCT-3′) and FcRAN1-R1 (5′-TTATTTCTAGTATAGTGGTCAGC-3′), were designed on the edge sites of Fc*RAN1*. The amplified products were purified and directly sequenced using a Big-Dye Terminator v3.1 Cycle Sequencing kit on an ABI 3730xl platform (Applied Biosystems, Sunnyvale, CA, USA).

To determine gene expression profile including *RAN1* expression, RNA-seq reads (two sexes fruits^11^, leaf and stem) were aligned to the fig genome sequence using TopHat^49^ and summarized.

## Additional Information

**Accession codes:** The fig genome data have been deposited in DDBJ/EMBL/GenBank under accession code BDEM01000001-BDEM01027995 (27995 entries). Sequence reads of transcriptome sequencing have been deposited in the NCBI sequence read archive under accession code DRA004650.

**How to cite this article**: Mori, K. *et al*. Identification of *RAN1* orthologue associated with sex determination through whole genome sequencing analysis in fig (*Ficus carica* L.). *Sci. Rep.* **7**, 41124; doi: 10.1038/srep41124 (2017).

**Publisher's note:** Springer Nature remains neutral with regard to jurisdictional claims in published maps and institutional affiliations.

## Supplementary Material

Supplementary Information

Supplementary Dataset 1

Supplementary Dataset 2

Supplementary Dataset 3

Supplementary Dataset 4

Supplementary Dataset 5

Supplementary Dataset 6

Supplementary Dataset 7

Supplementary Dataset 8

## Figures and Tables

**Figure 1:**
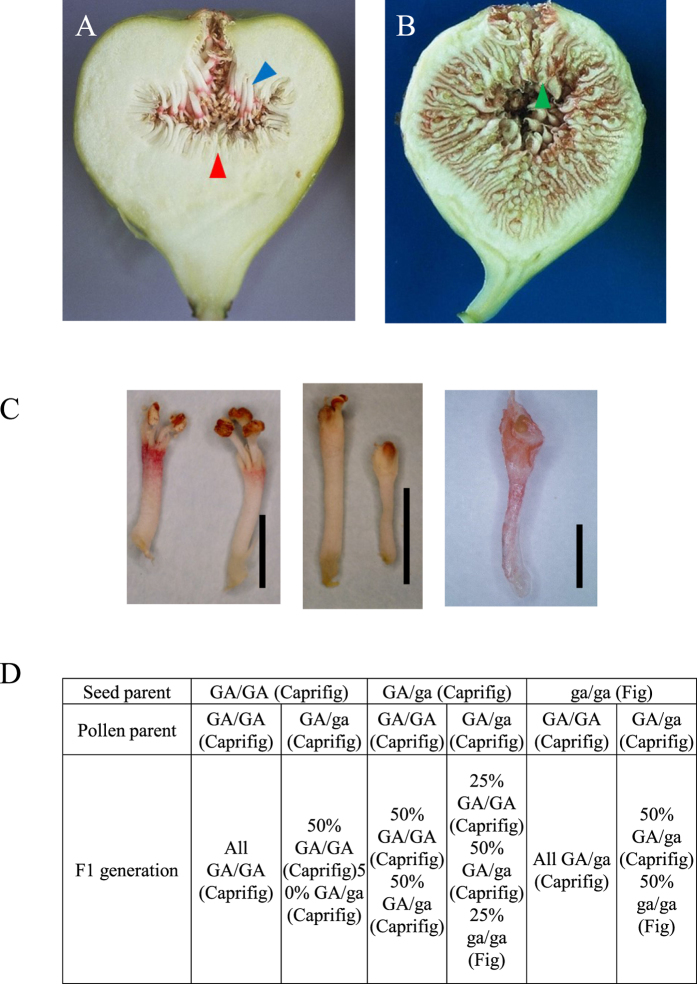
(**A**) Monoecious caprifig fruit and (**B**) female fig fruit. Blue, red and green arrowheads indicate staminate flower, short-style pistillate flower and long style pistillate flower, respectively. (**C**) caprifig staminate flowers (left), caprifig short-style pistillate flowers (center) and fig long-style pistillate flower (right). (**D**) Genetics of sex determination in F. carica. G, dominant allele for gynoecious flowers short-style pistils; g, recessive allele for gynoecious flowers short-style pistils; A, dominant allele for presence of the androecium; a, recessibe allele for suppression of the androecium. Two genes G/g and A/a are considered to be closely linked. Table was generated in reference to Storey (1975).

**Table 1:** Fig genome assembly and annotation statistics.

Estimate of genome size	356 Mb
Number of scaffolds (100 bp)	27,995
Total size of assembled scaffolds	248 Mb
N50 (scaddolds)	166 kb
Longest scaffold	1.7 Mb
Number of contigs	2,807,457
Total size of assembled contigs	466 Mb
N50 (contigs)	241b
Longest contig	10,794b
GC content	33.38%
Number of gene models	36,138
Number of annotated gene models	25,011
Rate of annotated gene models	69.2%
Interspersed or simple repeats masked	49.5 Mb
Repeat masking rate	20.0%
Total size of transposable elements	3,061,239
Transposable elements share in genome	1.24%

**Table 2:** List of high and moderate impact sex associated SNPs identified in the scaffold seq000259.

scaffold position	gene ID	REF	ALT	mutation type	impact	blast_nr description	accession_id	E-value
430	s00259g14130.t1	C	T	missense_variant	MODERATE	No hits found	—	—
433	s00259g14130.t1	C	T	missense_variant	MODERATE	No hits found	—	—
543	s00259g14130.t1	T	C	missense_variant	MODERATE	No hits found	—	—
9876	s00259g14131.t1	T	C	missense_variant	MODERATE	Copper-transporting ATPase RAN1 [Morus notabilis]	ref|XP_010087932.1	0
9900	s00259g14131.t1	C	T	missense_variant	MODERATE	Copper-transporting ATPase RAN1 [Morus notabilis]	ref|XP_010087932.1	0
12314	s00259g14131.t1	C	T	missense_variant	MODERATE	Copper-transporting ATPase RAN1 [Morus notabilis]	ref|XP_010087932.1	0
12722	s00259g14131.t1	C	G	missense_variant	MODERATE	Copper-transporting ATPase RAN1 [Morus notabilis]	ref|XP_010087932.1	0
12743	s00259g14131.t1	A	G	missense_variant	MODERATE	Copper-transporting ATPase RAN1 [Morus notabilis]	ref|XP_010087932.1	0
16247	s00259g14132.t1	C	A	missense_variant	MODERATE	No hits found	—	—
16262	s00259g14132.t1	G	A	missense_variant	MODERATE	No hits found	—	—
16332	s00259g14132.t1	G	A	missense_variant	MODERATE	No hits found	—	—
16431	s00259g14132.t1	G	T	missense_variant	MODERATE	No hits found	—	—
16644	s00259g14132.t1	A	G	missense_variant	MODERATE	No hits found	—	—
33672	s00259g14133.t1	G	A	missense_variant	MODERATE	Retrovirus-related Pol polyprotein from transposon TNT 1-94 [Morus notabilis]	ref|XP_010104938.1	5E-31
33676	s00259g14133.t1	C	T	missense_variant	MODERATE	Retrovirus-related Pol polyprotein from transposon TNT 1–94 [Morus notabilis]	ref|XP_010104938.1	5E-31
33702	s00259g14133.t1	G	A	missense_variant	MODERATE	Retrovirus-related Pol polyprotein from transposon TNT 1–94 [Morus notabilis]	ref|XP_010104938.1	5E-31
33801	s00259g14133.t1	G	C	missense_variant	MODERATE	Retrovirus-related Pol polyprotein from transposon TNT 1–94 [Morus notabilis]	ref|XP_010104938.1	5E-31
33951	s00259g14133.t1	A	G	missense_variant	MODERATE	Retrovirus-related Pol polyprotein from transposon TNT 1–94 [Morus notabilis]	ref|XP_010104938.1	5E-31
36750	s00259g14134.t1	G	T	missense_variant	MODERATE	hypothetical protein VITISV_041073 [Vitis vinifera]	emb|CAN76196.1	5E-35
36821	s00259g14134.t1	T	C	splice_acceptor_variant&intron_variant	HIGH	hypothetical protein VITISV_041073 [Vitis vinifera]	emb|CAN76196.1	5E-35
36935	s00259g14134.t1	C	T	missense_variant	MODERATE	hypothetical protein VITISV_041073 [Vitis vinifera]	emb|CAN76196.1	5E-35
37211	s00259g14134.t1	G	A	missense_variant	MODERATE	hypothetical protein VITISV_041073 [Vitis vinifera]	emb|CAN76196.1	5E-35
37307	s00259g14134.t1	C	A	missense_variant	MODERATE	hypothetical protein VITISV_041073 [Vitis vinifera]	emb|CAN76196.1	5E-35
38048	s00259g14134.t1	A	T	missense_variant	MODERATE	hypothetical protein VITISV_041073 [Vitis vinifera]	emb|CAN76196.1	5E-35
39084	s00259g14135.t1	A	T	missense_variant	MODERATE	Farnesylcysteine lyase [Morus notabilis]	ref|XP_010094602.1	0
39144	s00259g14135.t1	C	A	missense_variant	MODERATE	Farnesylcysteine lyase [Morus notabilis]	ref|XP_010094602.1	0
41088	s00259g14135.t1	C	T	missense_variant	MODERATE	Farnesylcysteine lyase [Morus notabilis]	ref|XP_010094602.1	0
45750	s00259g14136.t1	GA	G	frameshift_variant	HIGH	No hits found	—	—
45832	s00259g14136.t1	G	A	missense_variant	MODERATE	No hits found	—	—
45840	s00259g14136.t1	A	G	missense_variant	MODERATE	No hits found	—	—
64179	s00259g14139.t1	A	T	missense_variant	MODERATE	pentatricopeptide repeat-containing protein At2g16880-like [Malus domestica]	ref|XP_008362682.1	0
65387	s00259g14139.t1	G	A	missense_variant	MODERATE	pentatricopeptide repeat-containing protein At2g16880-like [Malus domestica]	ref|XP_008362682.1	0
67378	s00259g14140.t1	G	A	missense_variant	MODERATE	uncharacterized protein LOC103426396 [Malus domestica]	ref|XP_008362712.1	2E-93
68029	s00259g14140.t1	G	T	missense_variant	MODERATE	uncharacterized protein LOC103426396 [Malus domestica]	ref|XP_008362712.1	2E-93
68033	s00259g14140.t1	C	A	stop_gained	HIGH	uncharacterized protein LOC103426396 [Malus domestica]	ref|XP_008362712.1	2E-93
68512	s00259g14140.t1	G	A	missense_variant	MODERATE	uncharacterized protein LOC103426396 [Malus domestica]	ref|XP_008362712.1	2E-93
68583	s00259g14140.t1	G	A	missense_variant	MODERATE	uncharacterized protein LOC103426396 [Malus domestica]	ref|XP_008362712.1	2E-93
71075	s00259g14141.t1	C	T	missense_variant	MODERATE	Putative retroelement polyprotein [Arabidopsis thaliana]	gb|AAG10812.1	2E-81
100369	s00259g14144.t1	G	T	missense_variant	MODERATE	No hits found	—	—
