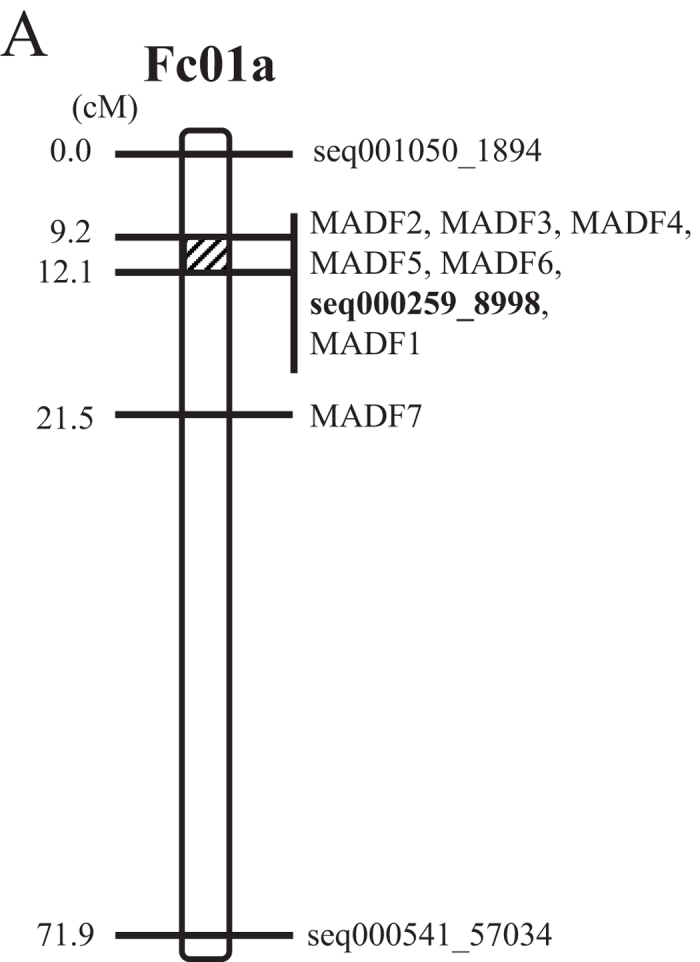# Corrigendum: Identification of RAN1 orthologue associated with sex determination through whole genome sequencing analysis in fig (*Ficus carica* L.)

**DOI:** 10.1038/srep46784

**Published:** 2017-05-10

**Authors:** Kazuki Mori, Kenta Shirasawa, Hitoshi Nogata, Chiharu Hirata, Kosuke Tashiro, Tsuyoshi Habu, Sangwan Kim, Shuichi Himeno, Satoru Kuhara, Hidetoshi Ikegami

Scientific Reports 7: Article number: 4112410.1038/srep41124; published online: 01 25 2017; updated: 05 10 2017

This Article contains errors. In the legend of Figure 1:

‘(D) Genetics of sex determination in F. carica. G, dominant allele for gynoecious flowers short-style pistils’

Should read:

‘(D) Genetics of sex determination in F. carica. G, dominant allele for gynoecious flowers long-style pistils’

In Table 1, row 4:

‘N50 (scaddolds)’

Should read:

‘N50 (scaffolds)’

In the Results section under the subheading ‘Genes involved in sex determination’:

‘Six of the markers were found to be located between positions 8.8 to 11.7 cM of the Fc01a linkage group, while the remaining one was on 21.5 cM of the Fc01a. This result indicated that the sex-determining A gene is likely located near the 8.8–11.7 cM region on the Fc01a linkage group.’

Should read:

‘Six of the markers were found to be located between positions 9.2 to 12.1 cM of the Fc01a linkage group, while the remaining one was on 21.5 cM of the Fc01a. This result indicated that the sex-determining A gene is likely located near the 9.2–12.1 cM region on the Fc01a linkage group’.

In the discussion:

‘Charlesworth described that change from cosexuality (monoecy) to dioecy probably involves a mutation creating females (a mutation suppressing some or all female flowers in an initially monoecious species, or replacing them with male flowers), and then one or more female-suppressing mutations, creating males or male-biased plants.’

Should read:

‘Charlesworth described that change from cosexuality (monoecy) to dioecy probably involves a mutation creating females (a mutation suppressing some or all male flowers in an initially monoecious species, or replacing them with female flowers), and then one or more female-suppressing mutations, creating males or male-biased plants.’

In addition, the legend of Figure 4:

(A) MADF (Male DNA Associated Sequence) markers and seq000259_8998 marker positioned at the diagonal region of Fc01a chromosome. (B) Top 5 GWAS detecting SNP markers. Most statistically significant SNP markers were mapped to the scaffold seq000259 on the Fc01a chromosome. seq000259_8998 marker genotypes completely matched the sex phenotypes of 119 test materials. (C) RAN1 gene structure, sex controlling and sex evolution model in fig. RAN1 is composed of 9 exons and 8 introns. Each of 2nd exon and 7th exon has one missense variations at 12,314th and 9,876th positon respectively.

Should read:

(A) MADF (Male Associated DNA Sequence in F. carica) markers and seq000259_8998 marker positioned at the diagonal region of Fc01a chromosome. (B) Top 5 GWAS detecting SNP markers. Most statistically significant SNP markers were mapped to the scaffold seq000259 on the Fc01a chromosome. seq000259_8998 marker genotypes completely matched the sex phenotypes of 119 test materials. (C) RAN1 gene structure in fig. RAN1 is composed of 9 exons and 8 introns. Each of 2nd exon and 7th exon has one missense variations at 12,314th and 9,876th positon respectively.

Further, this Article contains an error in Figure 4A, where the values of 8.8 and 11.7 should be 9.2 and 12.1 respectively. The correct Figure 4A appears below as Figure 1.

There were also errors in the Supplementary Information of this article.

In Supplementary Table S9 all instances of bisexual should be instead written as male.

In Supplementary Table S11 all instances of bisexual should be instead written as male and in row 0031 female should be written as male.

These errors have now been corrected in the Supplementary Information file that accompanies the Article. The PDF version was correct at the time of publication.

## Figures and Tables

**Figure 1:**