# Supplementary material for: Identification of RAN1 orthologue associated with sex determination through whole genome sequencing analysis in fig (Ficus carica L.)
Source: Sci Rep. 2017 Jan 25;7:41124. doi: 10.1038/srep41124 (PMC5264649; doi:10.1038/srep41124)
Supplement: Supplementary Information [file srep41124-s1.pdf]

Identification of *RAN1* orthologue associated with sex determination through whole genome sequencing analysis in fig (*Ficus carica* L.)

Kazuki Mori[1], Kenta Shirasawa[2], Hitoshi Nogata[3,4], Chiharu Hirata[5], Kosuke Tashiro[1], Tsuyoshi Habu[6], Sangwan Kim[1], Shuichi Himeno[3], Satoru Kuhara[1] & Hidetoshi Ikegami[3*]

[1]Faculty of Agriculture, Kyushu University, Fukuoka, Japan

[2]Kazusa DNA Research Institute, Kisarazu, Chiba, Japan

[3]Fukuoka Agriculture and Forestry Research Center Buzen Branch, Yukuhashi, Fukuoka, Japan

[4]Fukuoka Keichiku Agricultural Extension Center, Fukuoka, Japan

[5]Fukuoka Agriculture and Forestry Research Center, Fukuoka, Japan

[6]Graduate School of Agriculture, Ehime University, Ehime, Japan

[7] K. M., K. S. and H. I. contributed equally to this work.

*Address correspondence to: Hidetoshi Ikegami

E -mail: ikegami@farc.pref.fukuoka.jp
Present Address: Fukuoka Agriculture and Forestry Research Center Buzen Branch, 2-4-1 Nishiizumi, Yukuhashi, Fukuoka 824-0038, Japan

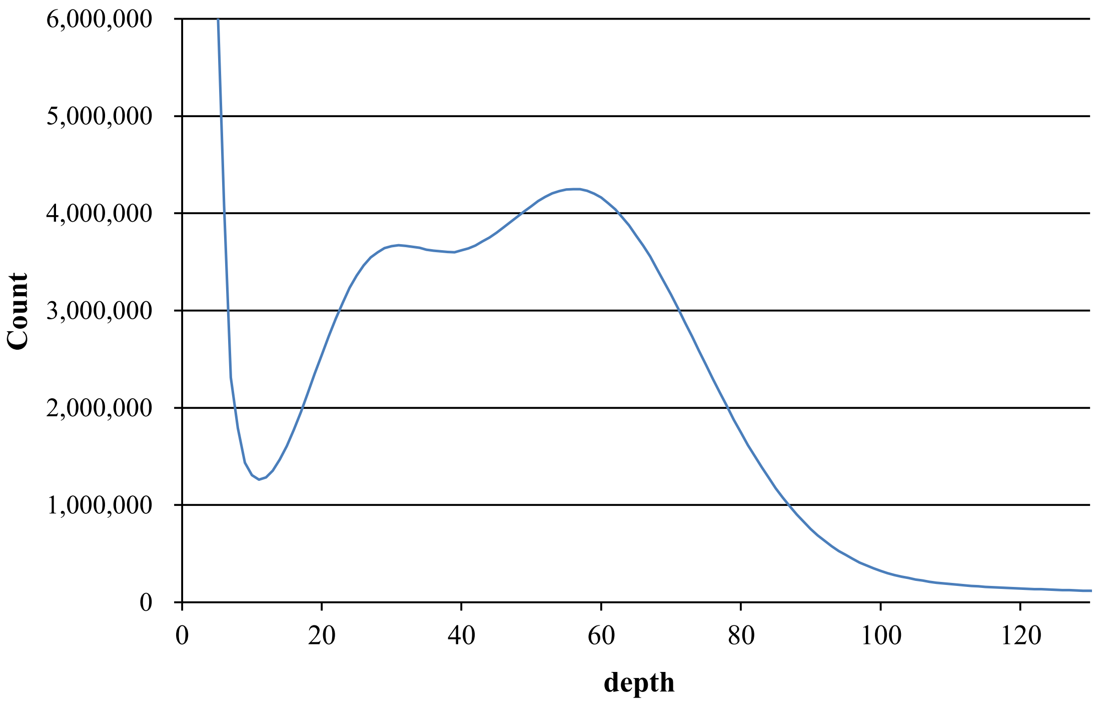

Supplementary Figure S1. 21-mer frequency distribution analysis of sequencing reads. A K-mer refers to an artificial sequence division of K nucleotides. The x-axis is depth (X); the y-axis is the proportion, which represents the frequency at that depth divide by the total frequency of all the depth. The high heterozygosis rate of jujube caused a sub peak (depth 30X) at the position of the half of the main peak (depth 60X).

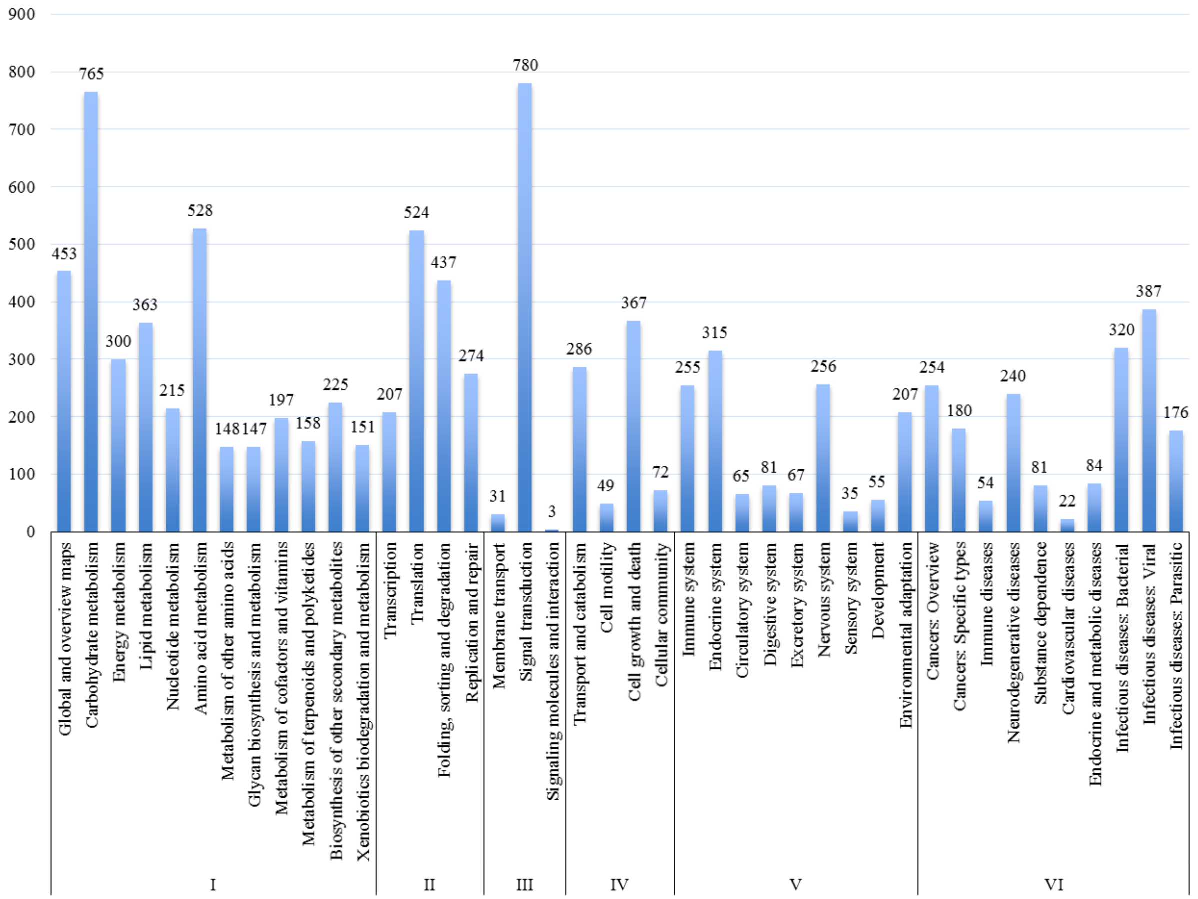

Supplementary Figure S2. Distribution of KEGG pathway frequency in the predicted gene set. I, Metabolism; II, Genetic Information Processing; III, Environmental Information Processing; IV, Cellular Processes; V, Organismal Systems; VI, Human Diseases

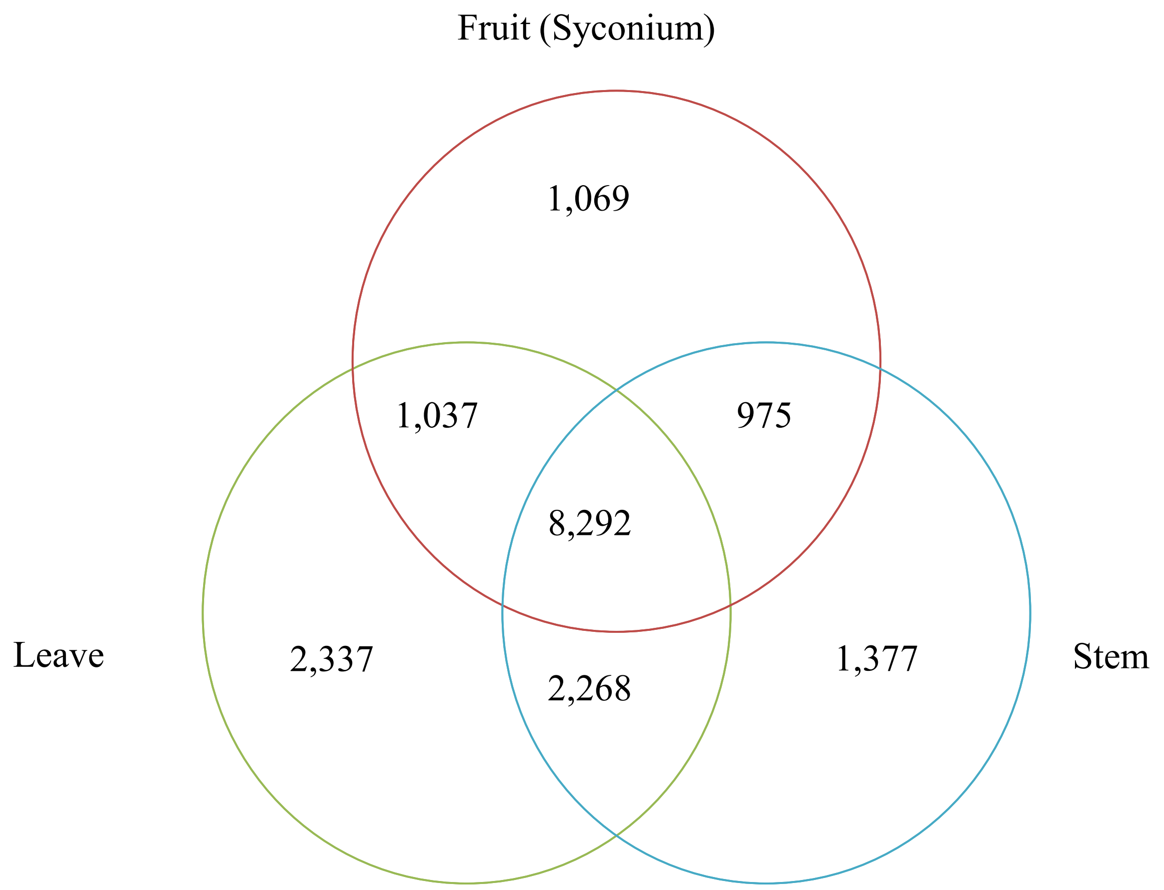

Supplementary Figure S3. Organ-specific gene expression. Venn diagram of expressed genes in three types of organs (Fruit, Leaf, Stem). 454 RNA-seq reads from each organ were mapped to reference fig genome and gene expression was estimated digitally.

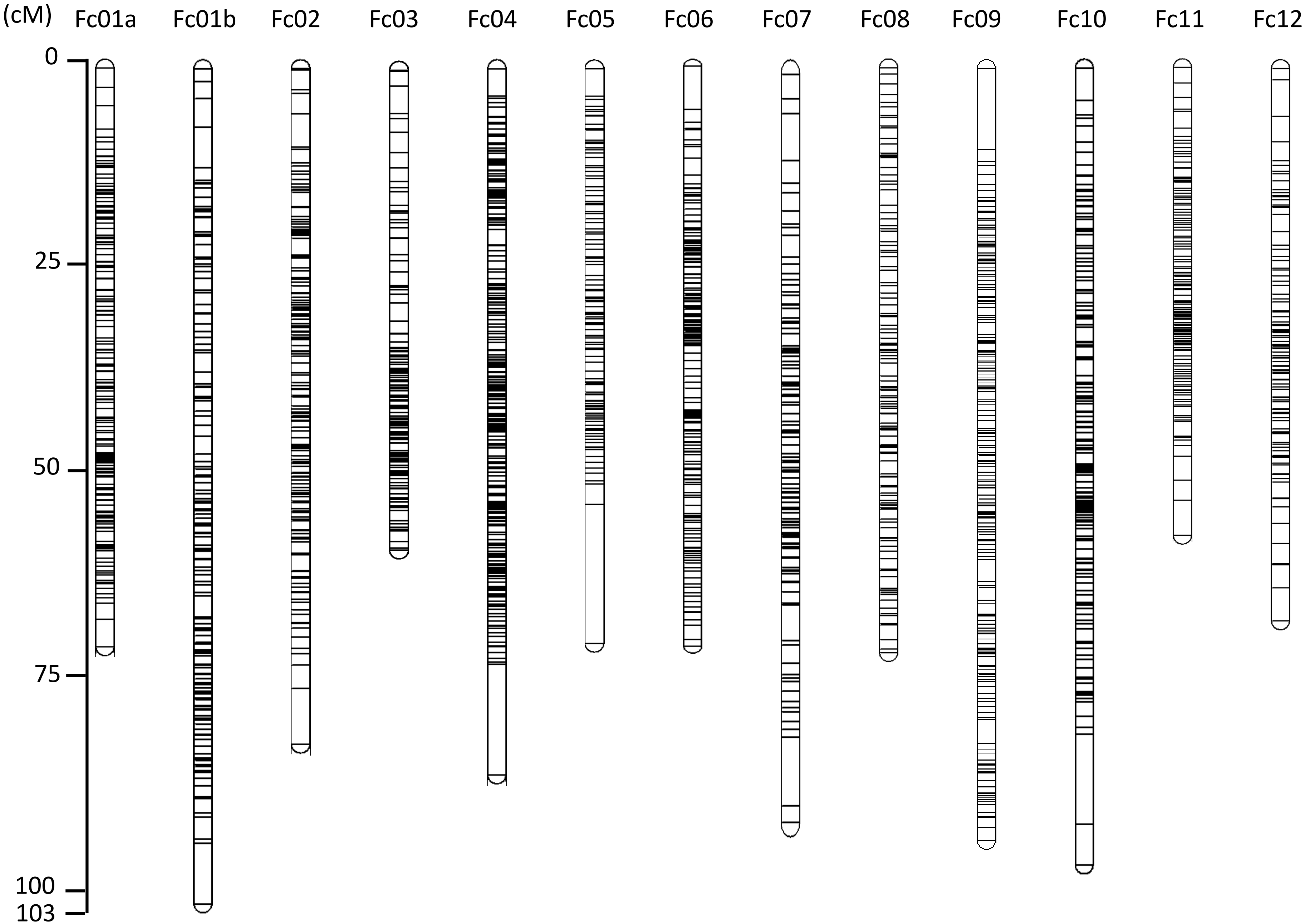

Supplementary Figure S4. The consensus genetic linkage map of the 13 chromosomes of *Ficus carica* L. We constructed of the genetic map with SSRs and SNP markers by JoinMap 4.

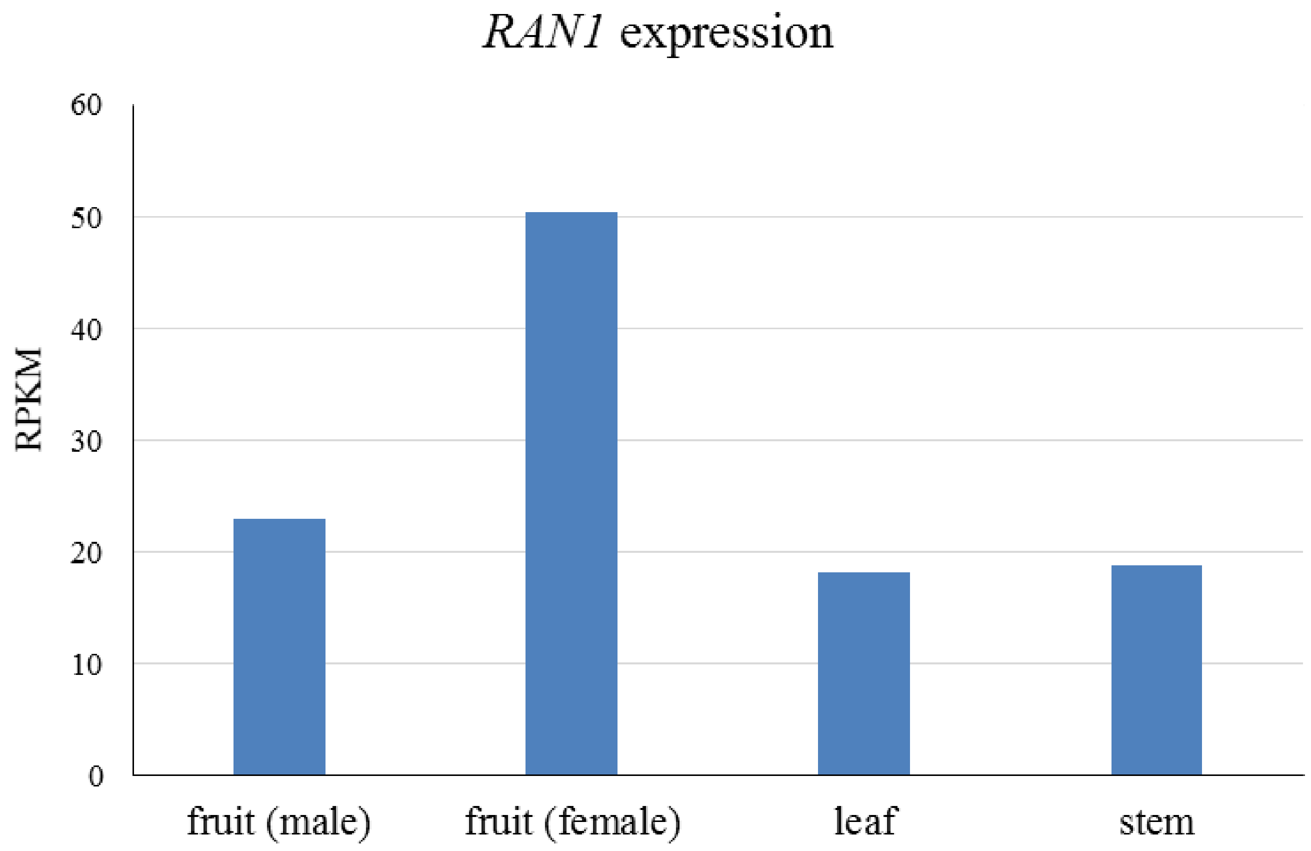

Supplementary Figure S5. Comparison of expression levels (measured as RPKM) for *RAN1* in fruit (from male and female), leaf and stem of fig.

*Pci*I – 1

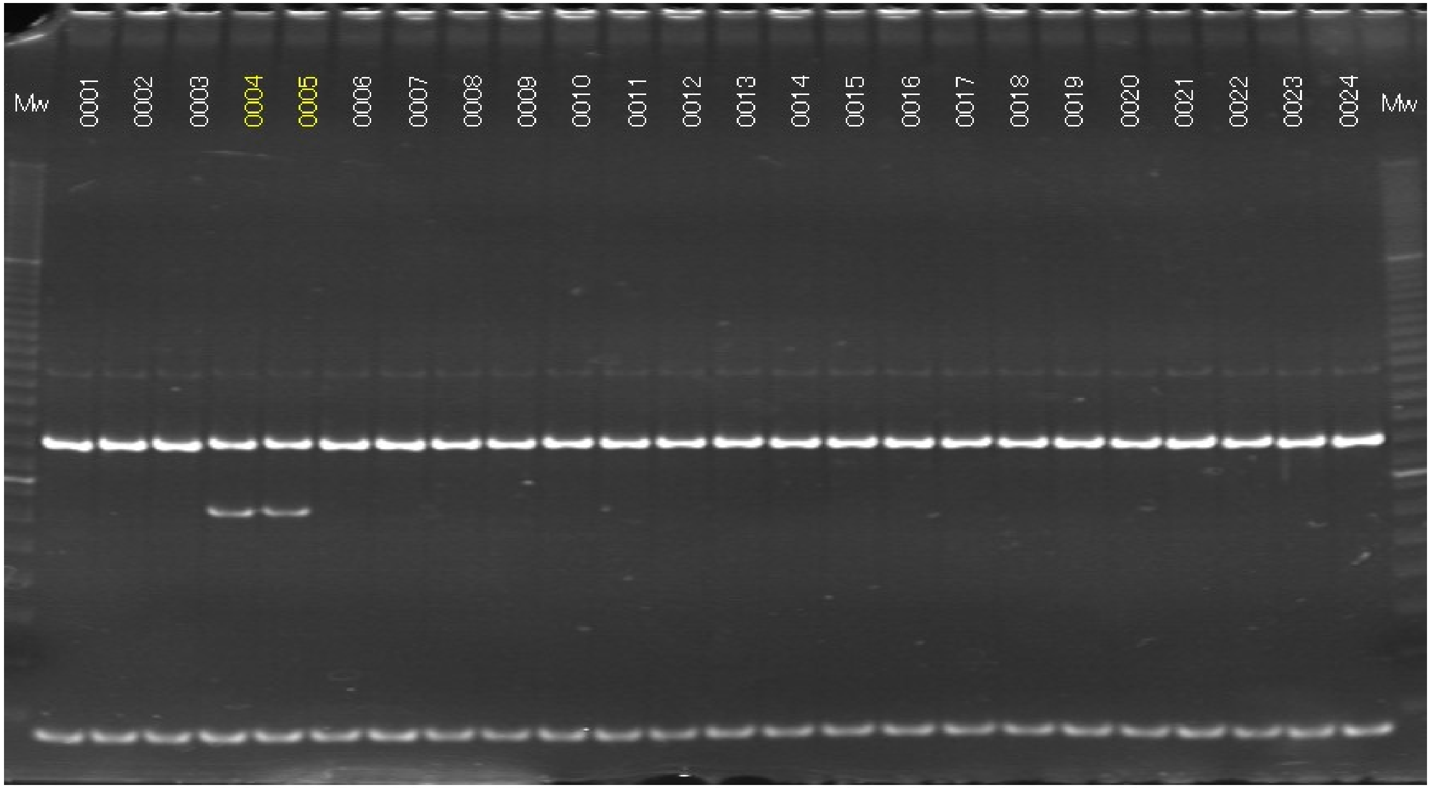

Supplementary Figure S6. CAPS marker analysis of 122 individuals detecting three alleles (CAT, CGT and TGT) using developed PCR primers. The band patterns with digested fragment are completely associated with male phenotypes.

*Pci*I – 2

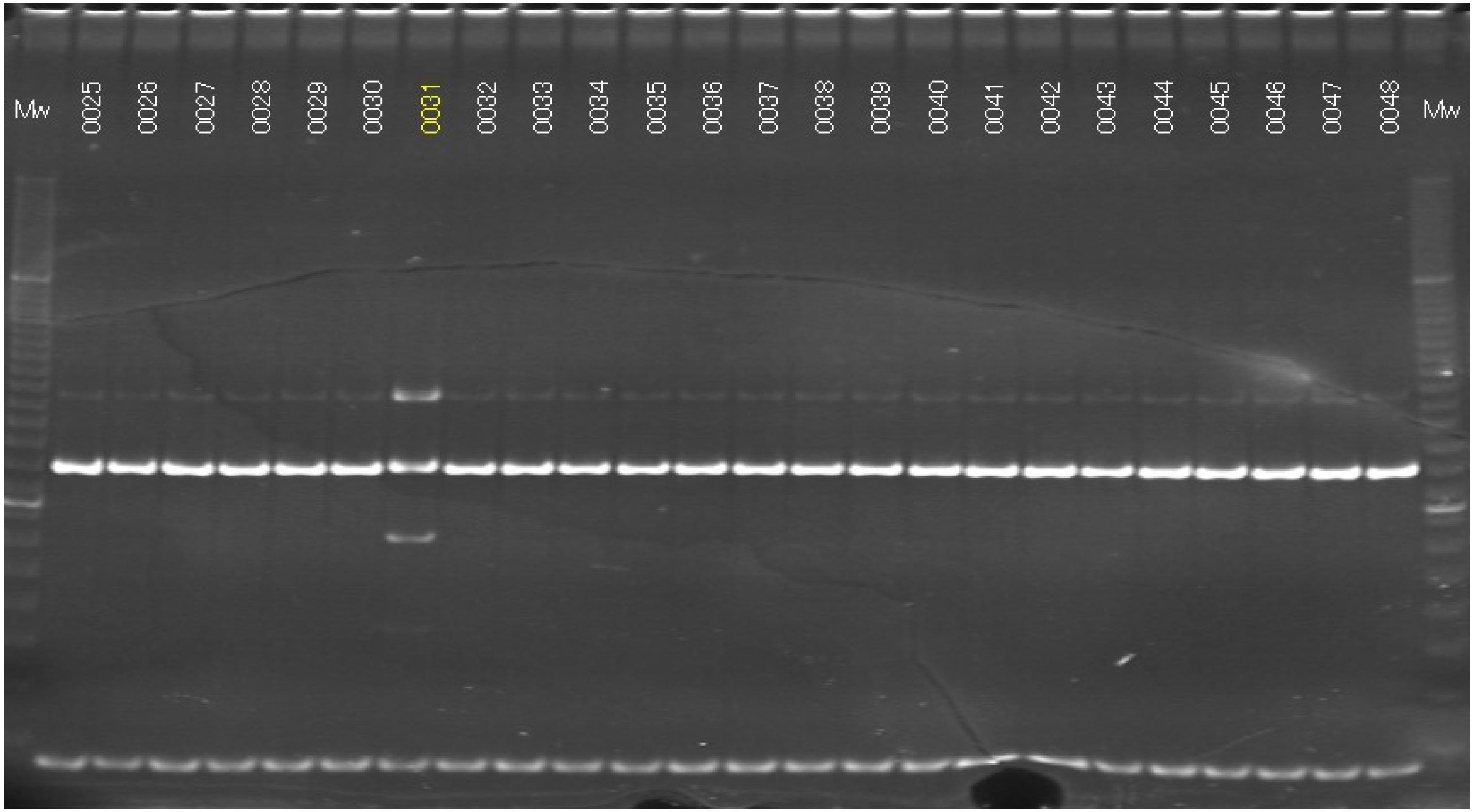

Supplementary Figure S6. CAPS marker analysis of 122 individuals detecting three alleles (CAT, CGT and TGT) using developed PCR primers. (Continued)

*Pci*I – 3

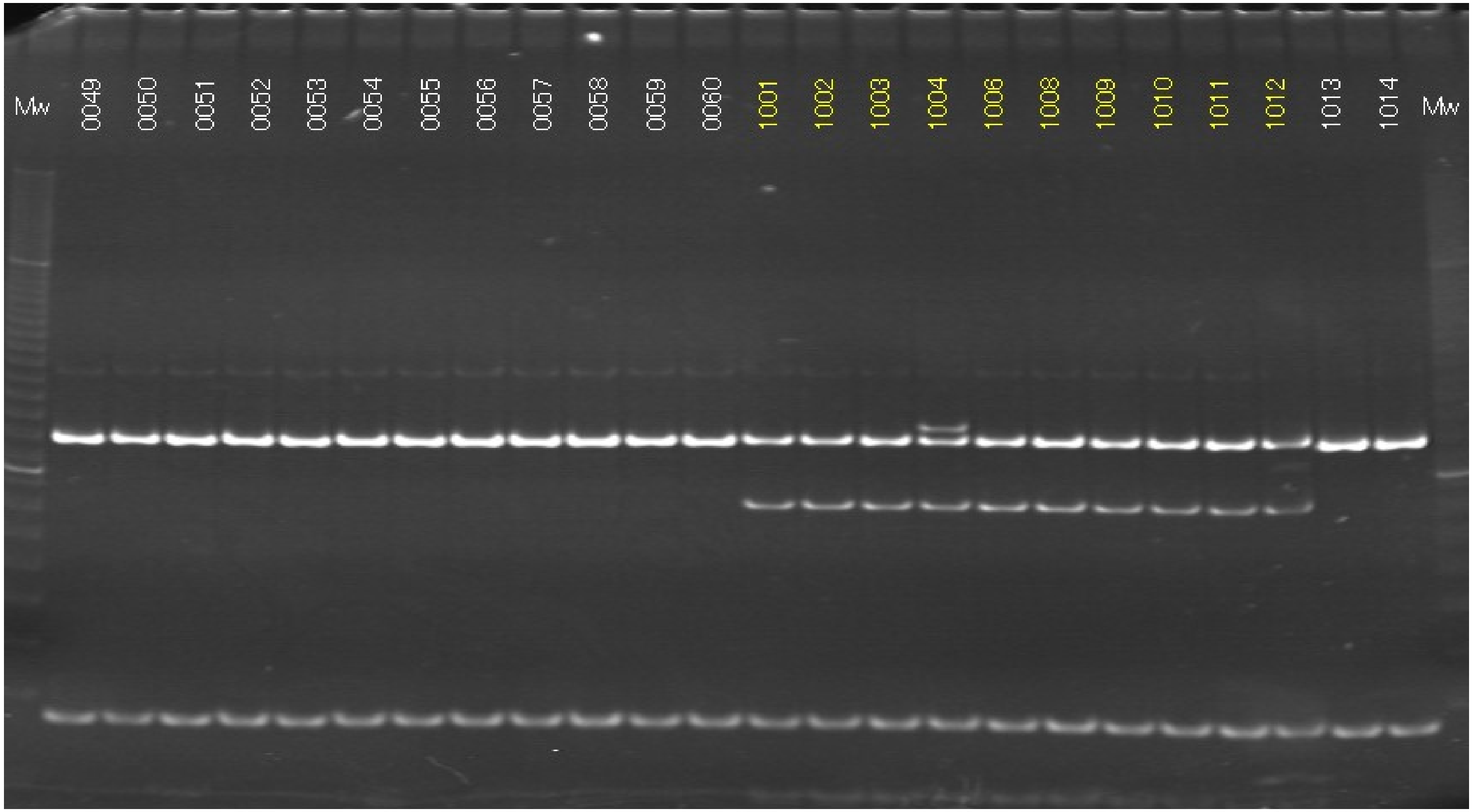

Supplementary Figure S6. CAPS marker analysis of 122 individuals detecting three alleles (CAT, CGT and TGT) using developed PCR primers. (Continued)

*Pci*I – 4

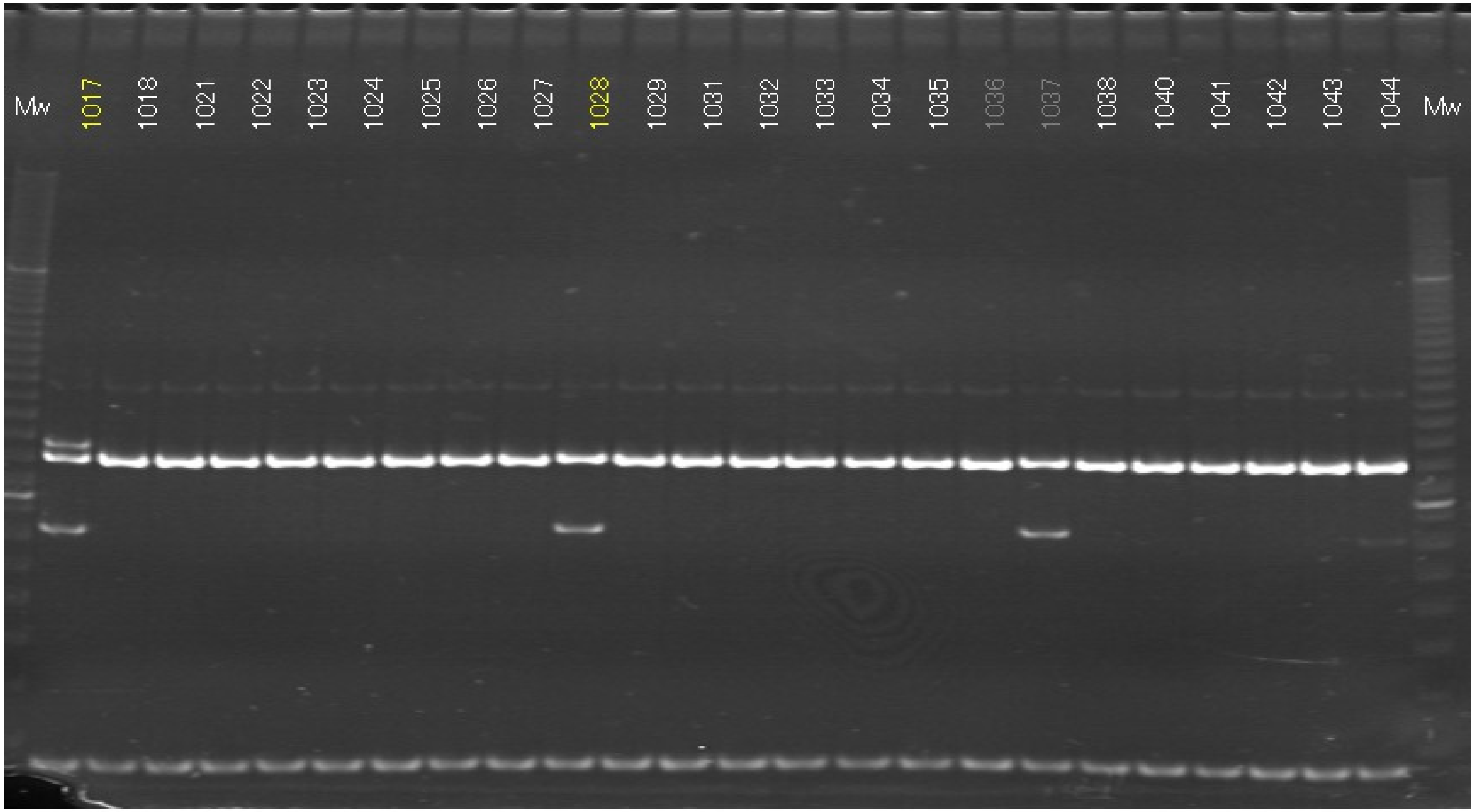

Supplementary Figure S6. CAPS marker analysis of 122 individuals detecting three alleles (CAT, CGT and TGT) using developed PCR primers. (Continued)

*Pci*I – 5

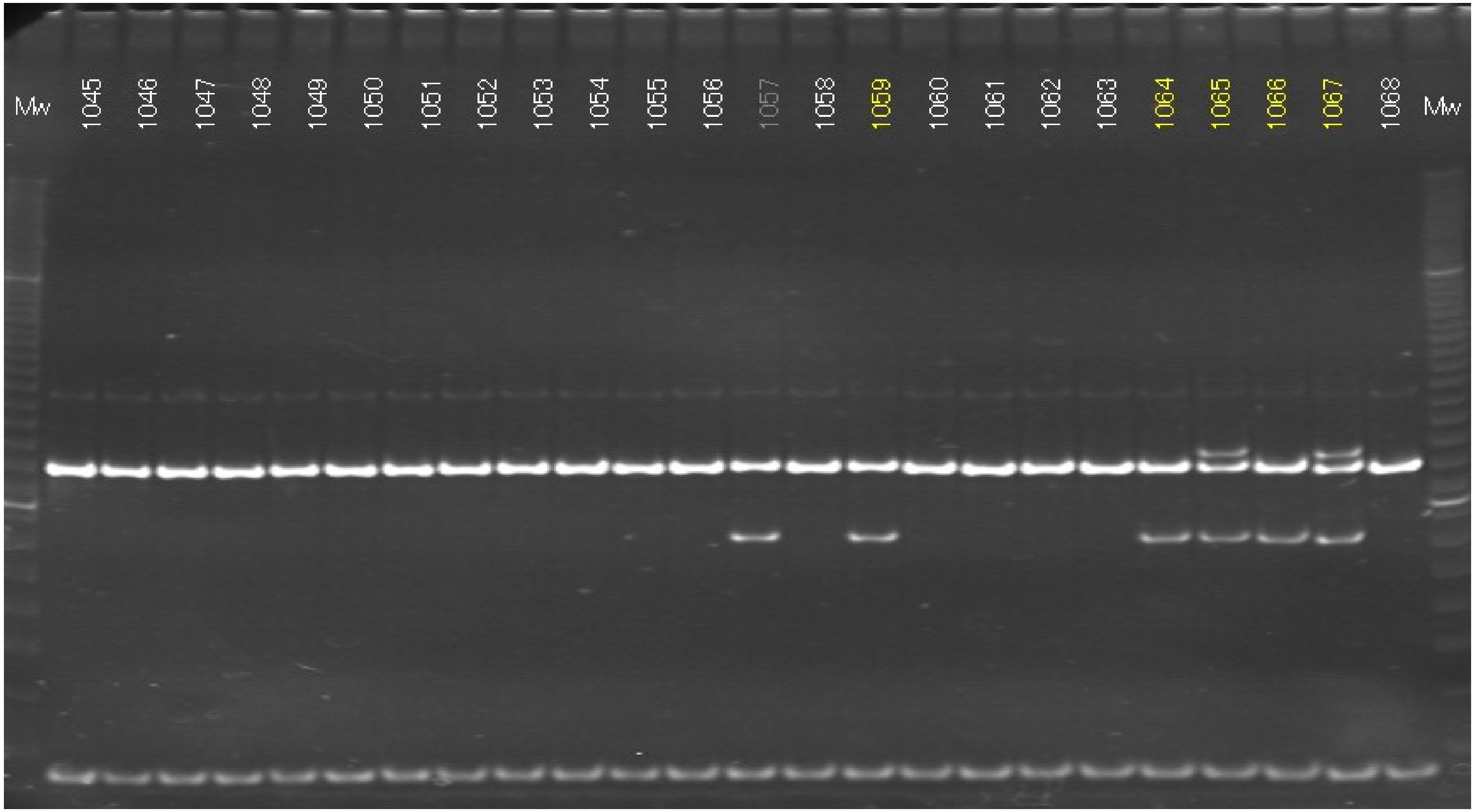

Supplementary Figure S6. CAPS marker analysis of 122 individuals detecting three alleles (CAT, CGT and TGT) using developed PCR primers. (Continued)

*Pci*I – 6

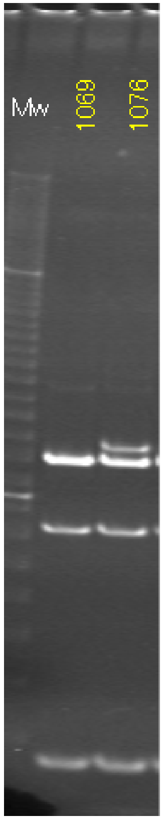

Supplementary Figure S6. CAPS marker analysis of 122 individuals detecting three alleles (CAT, CGT and TGT) using developed PCR primers. (Continued)

HpyCH4IV – 1

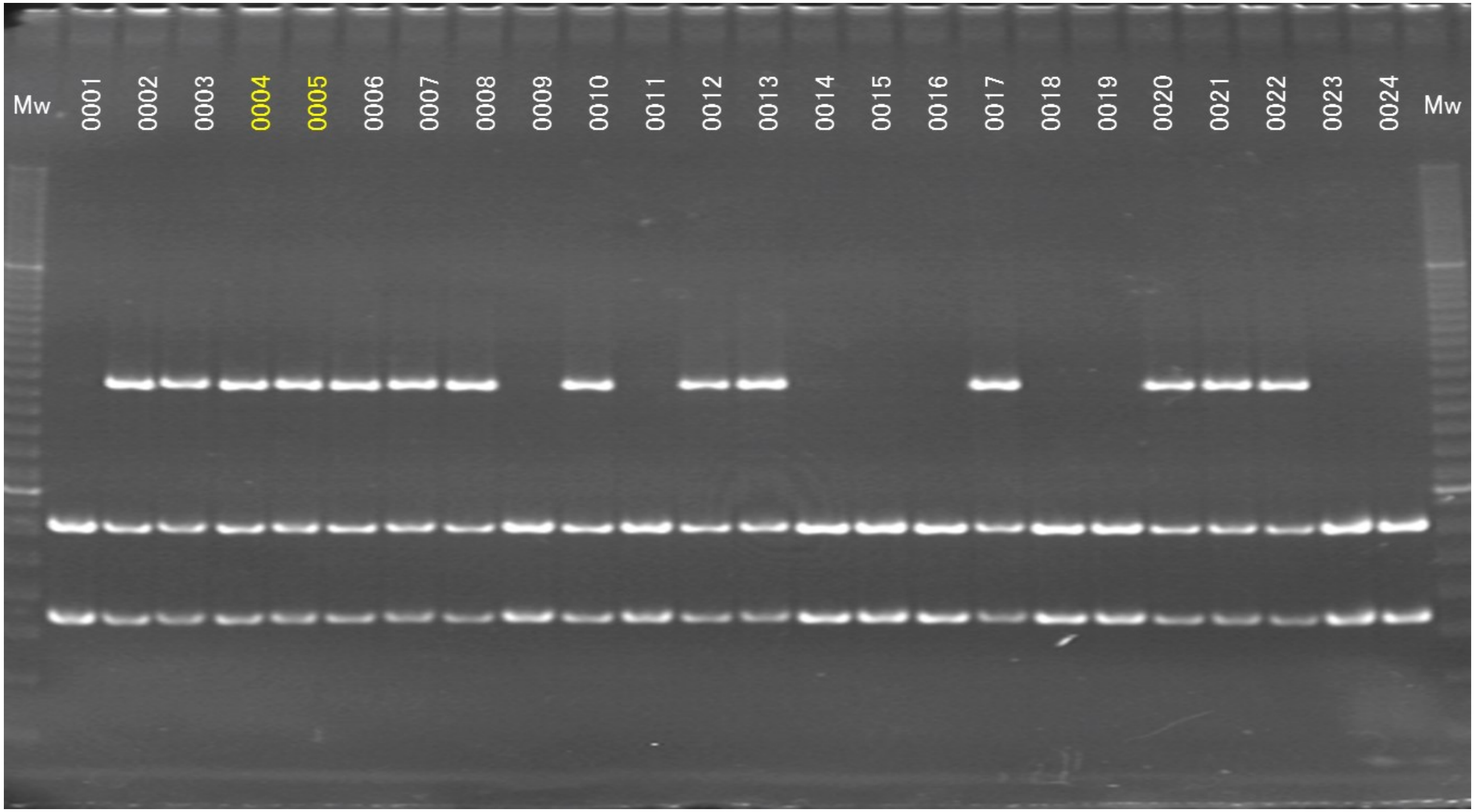

Supplementary Figure S6. CAPS marker analysis of 122 individuals detecting three alleles (CAT, CGT and TGT) using developed PCR primers. (Continued)

HpyCH4IV – 2

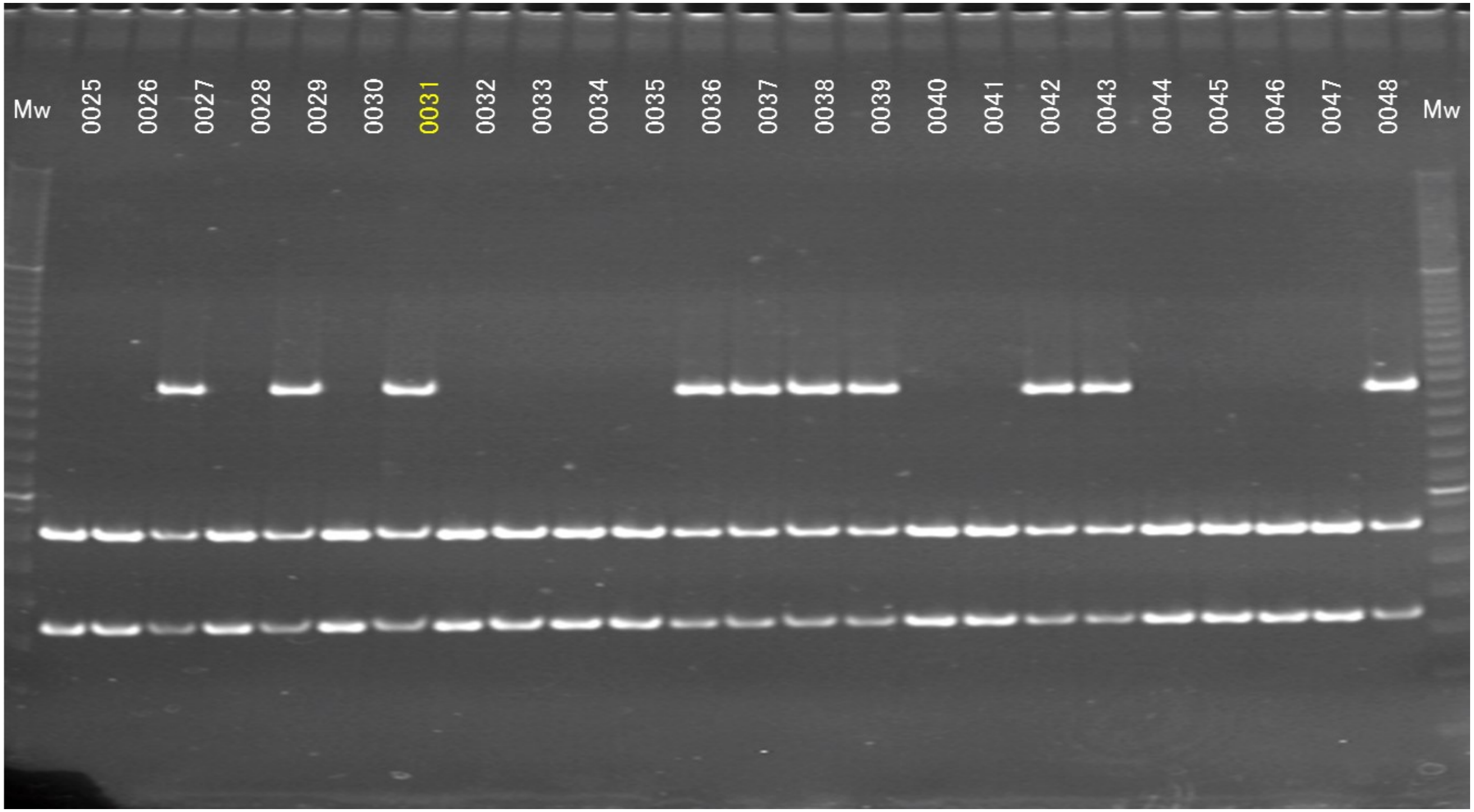

Supplementary Figure S6. CAPS marker analysis of 122 individuals detecting three alleles (CAT, CGT and TGT) using developed PCR primers. (Continued)

HpyCH4IV – 3

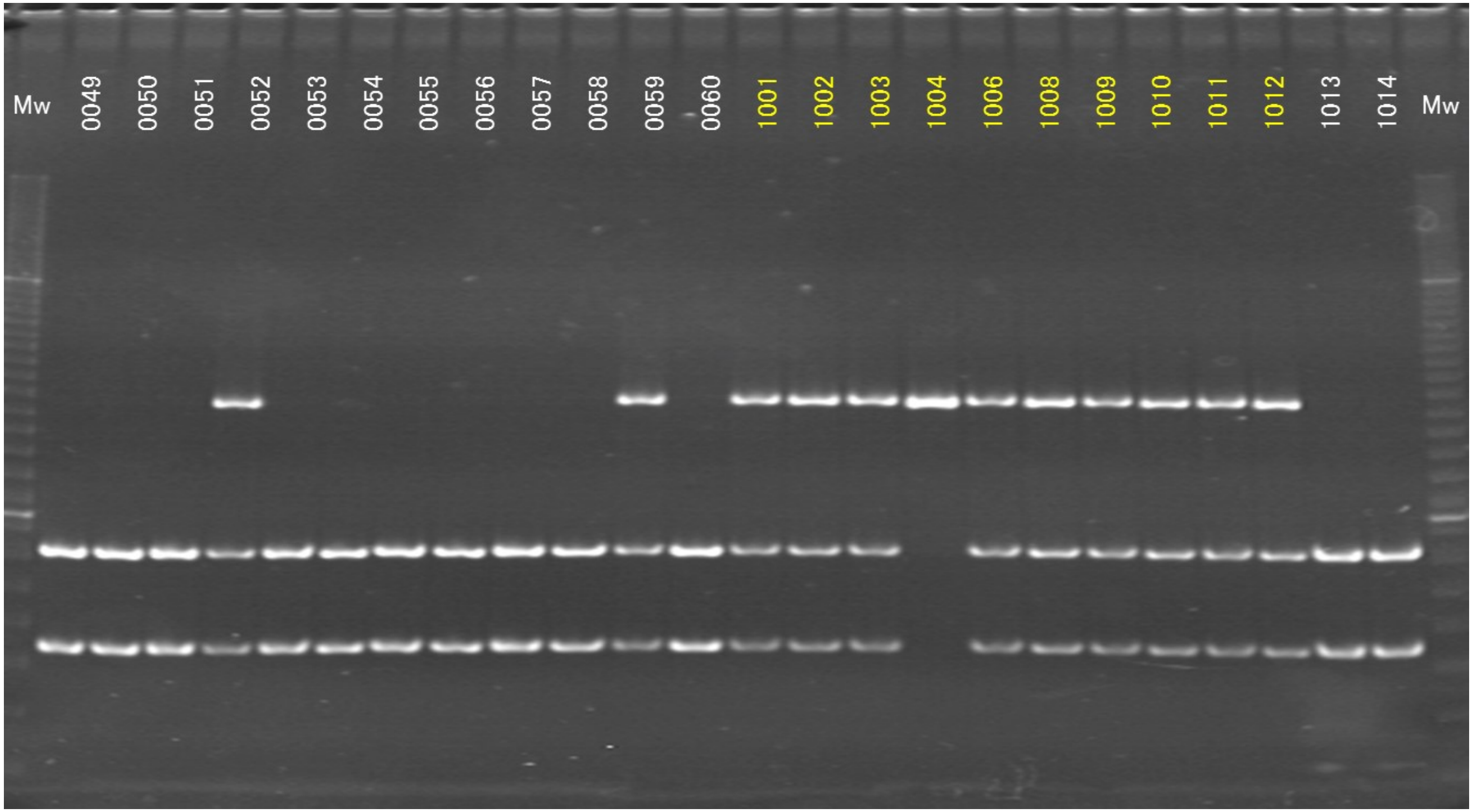

Supplementary Figure S6. CAPS marker analysis of 122 individuals detecting three alleles (CAT, CGT and TGT) using developed PCR primers. (Continued)

HpyCH4IV – 4

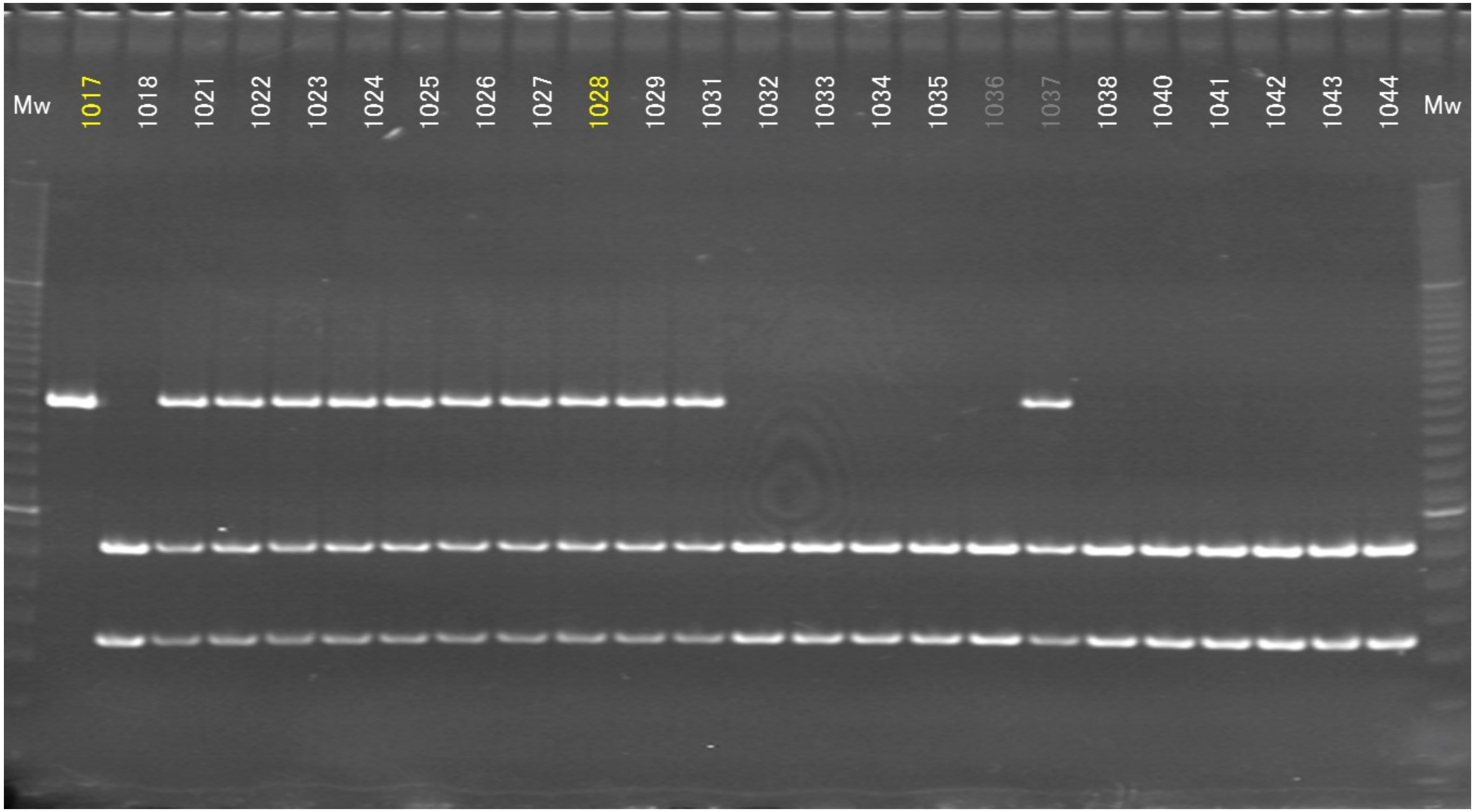

Supplementary Figure S6. CAPS marker analysis of 122 individuals detecting three alleles (CAT, CGT and TGT) using developed PCR primers. (Continued)

HpyCH4IV – 5

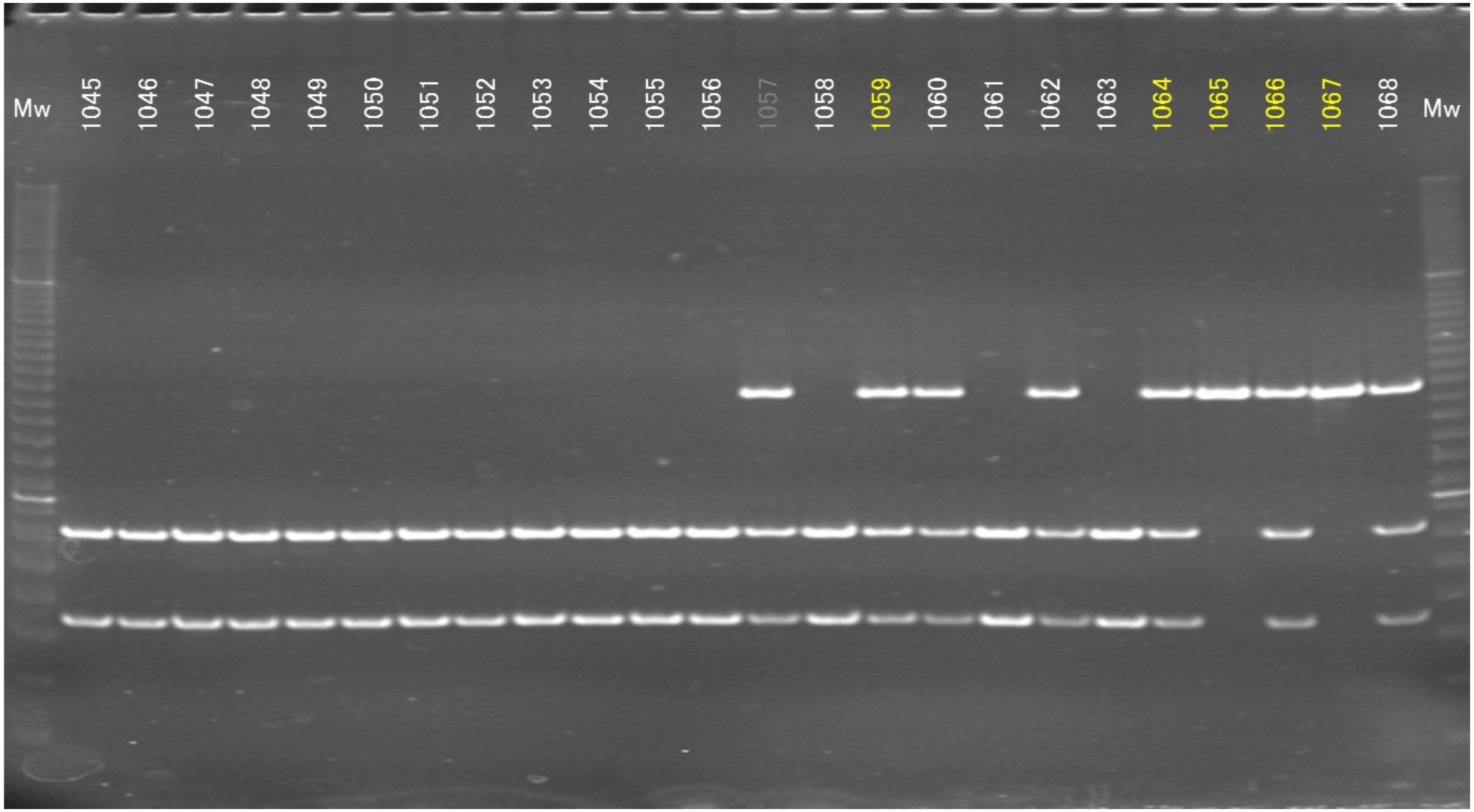

Supplementary Figure S6. CAPS marker analysis of 122 individuals detecting three alleles (CAT, CGT and TGT) using developed PCR primers. (Continued)

HpyCH4IV – 6

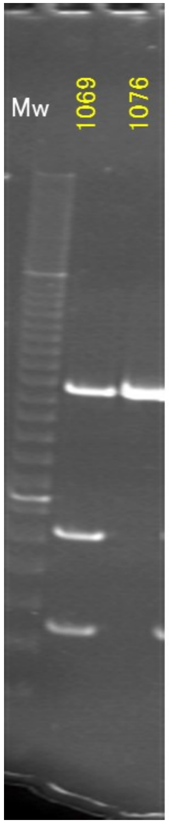

Supplementary Figure S6. CAPS marker analysis of 122 individuals detecting three alleles (CAT, CGT and TGT) using developed PCR primers. (Continued)

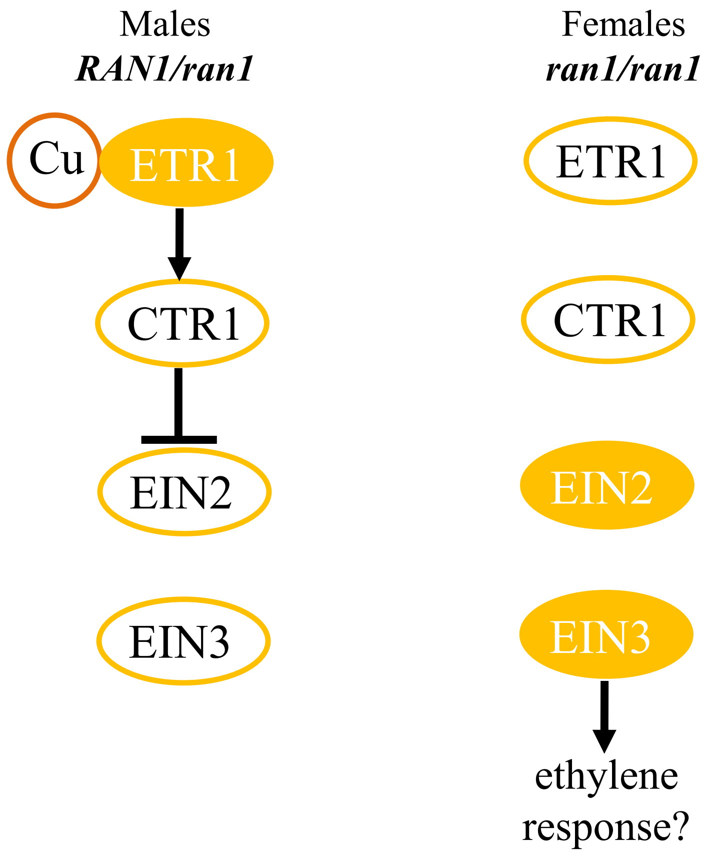

Supplementary Figure S7. The hypothesis of the relationship between *RAN1* genotypes and ethylene responses. In males, the receptors are active through the incorporation of copper ions and function negatively regulate downstream signaling pathway components, preventting hormone responsephenotypes. By contrast, the receptor are nonfunctional due to reduced copper transport, resulting in a activated ethylene signaling pathway in females.

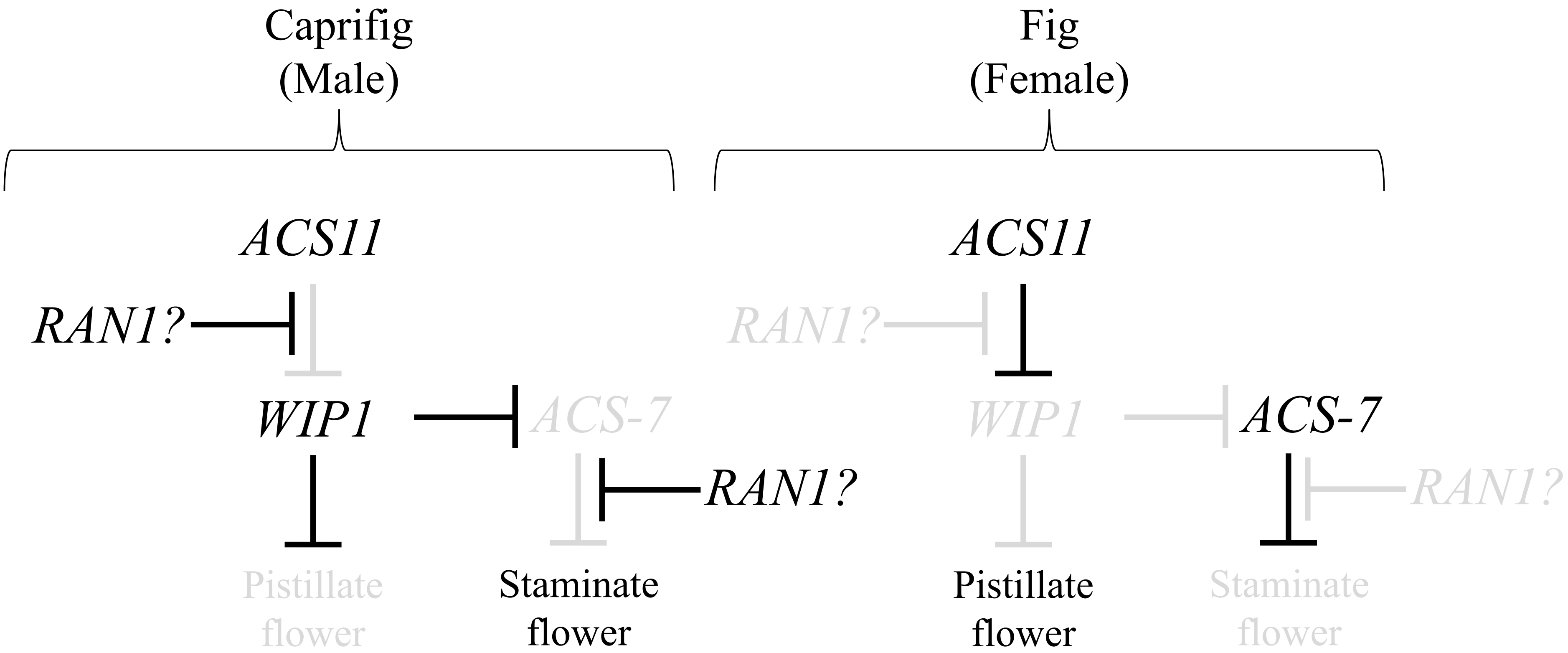

Supplementary Figure S8. A genetic and functional model of the fig sex determination integrating *ACS11*, *WIP1*, *ACS7* and *RAN1*. *RAN1* can neutralize the function of *ACS* genes through reduced ethylene sensitivity.

Supplementary Table S1. Summary of NGS sequencing for *F. carica* genome

NGS sequencers	Library type	Read length (bp)	Total length (bp)
Genome Analyzer Ⅱx (Illumina)	Pair end	75	8,829,348,600
MiSeq (Illumina)	Pair end	250	7,909,926,842
HiSeq 2000 (Illumina)	Mate pair 5kb	100	18,137,239,600
		sub total	34,876,515,042
		Total	34,876,515,042

Supplementary Table S2. Assessment of fig genome assemble and gene set in BUSCO notation (C:complete, F:fragmented, M:missing, n: gene number)

Size	BUSCO notation assessment results			
	C:	F:	M	n
248Mb	91%	5.2%	3.3%	956
36,138genes	90%	5.1%	4.6%	956

Supplementary Table S3. Annotation summary of predicted genes for *F. carica*

		Number	Rate (%)
Gene Models Total		36,138	-
Annotated	Blast nr	22,250	61.6
	InterPro	20,679	57.2
	GO	17,432	48.2
	KAAS K	6,288	17.4
	KAAS EC	2,810	7.8
	KAAS pathway	3,846	10.6
Unannotated		11,127	30.8

Supplementary Table S4. Repeat content in the *F. carica* draft genome.

Classification		Copy Number	Length (bp)	DNA Content (%)
Retroelements	SINEs:	2	140	0.00
	LINEs:	199	52,099	0.02
	LTR elements	4,379	2,789,729	1.13
	Ty1/Copia	2,276	1,335,055	0.54
	Gypsy/DIRS1	2,100	1,454,478	0.59
	Others	3	196	0.00
	Retroelements total	4,580	2,841,968	1.15
DNA transposons	hobo-Activator	88	19,431	0.01
	Tc1-IS630-Pogo	79	14,798	0.01
	En-Spm	11	5,963	0.00
	Tourist/Harbinger	23	6,781	0.00
	Others	973	172,298	0.07
	DNA transposon total	1,174	219,271	0.09
Unclassified elements		222,067	36,710,264	14.86
Transposable elements Total		227,821	39,771,503	16.10
Satellite		1	129	0.00
Simple repeat		260,203	9,553,761	3.87
Low complexity		46,904	2,378,715	0.96
Total		534,929	51,704,108	20.93

Supplementary Table S5. Preliminary restricution enzyme experiments for RAD-seq using F1 population (Horaishi ×Caprifig 6085).

Parent		Restriction enzyme site	
Horaishi	Caprifig 6085	PstI-EcoRI (PE)	PstI-MspI (PM)
REF	Het	4,011	4,243
Het	REF	2,133	2,193
Het	Het	2,203	2,701
Het	ALT	2,006	2,020
ALT	Het	103	106

Supplementary Table S6. The RAD-seq alignment results of 52 plants for genetic linkage map construction.

name	input	pair aligned	single aligned	total aligned	alinment rate
HO-PM	1995800	1019340	262485	1281825	64.23%
caprifig6058-PM	1787950	773138	216039	989177	55.32%
HOcaprifigF1-01	4832442	3099892	356566	3456458	71.53%
HOcaprifigF1-02	4510730	2804244	394220	3198464	70.91%
HOcaprifigF1-03	5373047	3277444	449159	3726603	69.36%
HOcaprifigF1-04	4265030	2695652	356995	3052647	71.57%
HOcaprifigF1-05	3892605	2267306	350816	2618122	67.26%
HOcaprifigF1-06	4752380	2992792	383734	3376526	71.05%
HOcaprifigF1-07	4288142	2901870	308034	3209904	74.86%
HOcaprifigF1-08	4907463	2980580	390311	3370891	68.69%
HOcaprifigF1-09	5006757	3043624	416819	3460443	69.12%
HOcaprifigF1-10	5331994	3224002	444974	3668976	68.81%
HOcaprifigF1-11	6080926	3827216	490545	4317761	71.00%
HOcaprifigF1-12	5236580	2996154	442207	3438361	65.66%
HOcaprifigF1-13	4431024	2809618	364152	3173770	71.63%
HOcaprifigF1-14	5163006	3185322	433456	3618778	70.09%
HOcaprifigF1-15	4702750	2735670	418047	3153717	67.06%
HOcaprifigF1-16	5035519	3166976	405625	3572601	70.95%
HOcaprifigF1-17	5879291	3565596	505396	4070992	69.24%
HOcaprifigF1-18	5296004	3190256	471496	3661752	69.14%
HOcaprifigF1-19	6105990	3526148	541885	4068033	66.62%
HOcaprifigF1-20	5072234	2765894	494227	3260121	64.27%
HOcaprifigF1-21	4588422	2805196	392621	3197817	69.69%
HOcaprifigF1-22	5054191	2988198	440681	3428879	67.84%
HOcaprifigF1-23	4723768	2935082	399533	3334615	70.59%
HOcaprifigF1-24	4562430	2795856	384000	3179856	69.70%
HOcaprifigF1-25	5545001	3078212	474817	3553029	64.08%
HOcaprifigF1-26	5983813	3662602	482385	4144987	69.27%
HOcaprifigF1-27	5874316	3608370	482870	4091240	69.65%
HOcaprifigF1-28	5328310	3139906	477633	3617539	67.89%
HOcaprifigF1-29	4589603	2822676	370978	3193654	69.58%
HOcaprifigF1-30	5224541	2929940	427557	3357497	64.26%
HOcaprifigF1-31	4719220	2522610	438442	2961052	62.74%
HOcaprifigF1-32	4632692	2714068	404136	3118204	67.31%
HOcaprifigF1-33	6420556	3760010	525156	4285166	66.74%
HOcaprifigF1-34	5899329	3551726	504598	4056324	68.76%
HOcaprifigF1-35	5996619	3528728	520740	4049468	67.53%
HOcaprifigF1-36	5429684	3283802	475258	3759060	69.23%
HOcaprifigF1-37	4754185	2821646	410583	3232229	67.99%
HOcaprifigF1-38	5437888	3190368	489191	3679559	67.67%
HOcaprifigF1-39	5099334	2987092	440251	3427343	67.21%
HOcaprifigF1-40	5077754	2851248	451335	3302583	65.04%
HOcaprifigF1-41	5934718	3350168	502065	3852233	64.91%
HOcaprifigF1-42	6212100	3734682	531727	4266409	68.68%
HOcaprifigF1-43	6682105	3995908	571782	4567690	68.36%
HOcaprifigF1-44	6135807	3516020	537748	4053768	66.07%
HOcaprifigF1-45	4976907	3049024	428708	3477732	69.88%
HOcaprifigF1-46	5976080	3546882	505697	4052579	67.81%
HOcaprifigF1-47	4260978	2382496	240598	2623094	61.56%
HOcaprifigF1-48	4137974	2713496	298017	3011513	72.78%
HOcaprifigF1-49	6519731	4355458	452797	4808255	73.75%
HOcaprifigF1-50	6016513	3803954	481560	4285514	71.23%
HOcaprifigF1-51	7059406	4662060	502180	5164240	73.15%
HOcaprifigF1-52	6180914	3881750	496595	4378345	70.84%
Total	278,982,553	167,817,968	23,439,427	191,257,395	
Average	5,365,049	3,227,269	450,758	3,678,027	68.34%

Supplementary Table S7. Summary of genetic linkage map construction by RAD-seq in *F. carica*.

Group	Horaishi (Seed parent)		Caprifig6085 (pollen parent)		Integration		Density (cM)
	Number of Loci	Length(cM)	Number of Loci	Length(cM)	Number of Loci	Length(cM)	
Fc01a	404	77.7	445	82.5	755	71.9	0.10
Fc01b	269	98.5	341	94.7	526	102.9	0.20
Fc02	518	97.9	435	70.0	790	83.8	0.11
Fc03	264	83.6	214	53.0	397	60.4	0.15
Fc04	468	132.7	615	103.5	906	87.4	0.10
Fc05	263	61.1	318	76.4	485	71.5	0.15
Fc06	358	98.7	334	80.5	583	93.7	0.16
Fc07	267	81.6	243	55.5	424	58.6	0.14
Fc08	356	111.4	192	96.0	479	72.7	0.15
Fc09	448	99.9	416	100.0	729	95.4	0.13
Fc10	301	102.3	351	93.3	552	98.5	0.18
Fc11	266	70.8	324	85.0	512	58.5	0.11
Fc12	207	51.1	226	72.7	360	68.8	0.19
Total	4,389	1167.4	4,454	1063.2	7,498	1,024.1	
Average	337.6	89.8	342.6	81.8	576.8	78.8	0.14

Supplementary Table S8. Male associated DNA sequence (MADF) markers information and its genotyping results

Marker name	Foward/ Reverse	Primer sequence	Test population	No. of test plants	No. of male caprifigs	No. of female figs	No. of accurate calling	Accuracy rate (%)
MADF1	F	CAGCAATCCCTGATACAAG	F1 (Violette de Dauphine x M106-238)	71	36	35	69	97.2
	R	CAGCAATCCCATACATTATTC	F1 (Violette de Solies x VC-180)	58	20	38	56	96.6
			F1 (Royal Vineyard x VC-180)	62	24	38	60	96.8
			Cultivars	56	7	49	44	78.6
MADF2	F	TGACGATGCAGCAAGTGAAGGGC	F1 (Violette de Dauphine x M106-238)	71	36	35	71	100.0
	R	TGACGATGCACGACATTGGTGGATG	F1 (Violette de Solies x VC-180)	58	20	38	58	100.0
			F1 (Royal Vineyard x VC-180)	62	24	38	62	100.0
			Cultivars	56	7	49	56	100.0
MADF3	F	CCTGGGTCATCTACATAATAACC	F1 (Violette de Dauphine x M106-238)	71	36	35	71	100.0
	R	CCTGGGTCATTATAGAAAGAACC	F1 (Violette de Solies x VC-180)	58	20	38	58	100.0
			F1 (Royal Vineyard x VC-180)	62	24	38	62	100.0
			Cultivars	56	7	49	56	100.0
MADF4	F	TCTCTAGGGGGCAGCATGGACAGTTTTC	F1 (Violette de Dauphine x M106-238)	71	36	35	71	100.0
	R	TCTCTAGGGGGGGGGGGCGGATTTTTTG	F1 (Violette de Solies x VC-180)	58	20	38	58	100.0
			F1 (Royal Vineyard x VC-180)	62	24	38	62	100.0
			Cultivars	56	7	49	56	100.0
MADF5	F	GATCTAACCGAACTAGAAATGG	F1 (Violette de Dauphine x M106-238)	71	36	35	71	100.0
	R	GATCTAACCGCAAAACAAATGTC	F1 (Violette de Solies x VC-180)	58	20	38	58	100.0
			F1 (Royal Vineyard x VC-180)	62	24	38	62	100.0
			Cultivars	56	7	49	56	100.0
MADF6	F	ACACTCGGCAAGATGGGAAAGAG	F1 (Violette de Dauphine x M106-238)	71	36	35	71	100.0
	R	ACACTCGGCATCAGGAGGTC	F1 (Violette de Solies x VC-180)	58	20	38	58	100.0
			F1 (Royal Vineyard x VC-180)	62	24	38	62	100.0
			Cultivars	56	7	49	56	100.0
MADF7	F	GCTGAAGAAGGTAATTAGAGC	F1 (Violette de Dauphine x M106-238)	71	36	35	69	97.2
	R	GCTGAAGAAGAAGCTGAATTG	F1 (Violette de Solies x VC-180)	58	20	38	45	77.6
			F1 (Royal Vineyard x VC-180)	62	24	38	57	91.9
			Cultivars	56	7	49	48	85.7

Supplementary Table S9. Material list for GWAS of sex determination

species	ID	variety/accession name	sexuality	sampling location	annotation	synonymous name
Ficus carica	0001	Horaishi	female	FAFRC, BB Yukuhashi, Japan	variety	Horaigaki
	0002	Toyomitsuhime	female	FAFRC, BB Yukuhashi, Japan	variety	
	0003	Masui Dauphine	female	FAFRC, BB Yukuhashi, Japan	variety	
	0004	caprifig 6085	male	FAFRC, BB Yukuhashi, Japan	variety	
	0005	Capri Type	male	FAFRC, BB Yukuhashi, Japan	variety	
	0006	Archipel	female	FAFRC, BB Yukuhashi, Japan	variety	
	0007	Atena	female	FAFRC, BB Yukuhashi, Japan	variety	
	0008	Acherb Fangen Black	female	FAFRC, BB Yukuhashi, Japan	variety	
	0009	Achicudo Argentiju	female	FAFRC, BB Yukuhashi, Japan	variety	
	0010	Allison	female	FAFRC, BB Yukuhashi, Japan	variety	
	0011	Ischia White	female	FAFRC, BB Yukuhashi, Japan	variety	
	0012	Ischia Black	female	FAFRC, BB Yukuhashi, Japan	variety	
	0013	Osborn Prolific	female	FAFRC, BB Yukuhashi, Japan	variety	
	0014	Kadota	female	FAFRC, BB Yukuhashi, Japan	variety	
	0015	California Black	female	FAFRC, BB Yukuhashi, Japan	variety	
	0016	King	female	FAFRC, BB Yukuhashi, Japan	variety	
	0017	Kohaku	female	FAFRC, BB Yukuhashi, Japan	variety	Daw Low
	0018	Conadria	female	FAFRC, BB Yukuhashi, Japan	variety	
	0019	San Pedro White	female	FAFRC, BB Yukuhashi, Japan	variety	
	0020	Short Bridge	female	FAFRC, BB Yukuhashi, Japan	variety	
	0021	Genoa White	female	FAFRC, BB Yukuhashi, Japan	variety	
	0022	Zebra Sweet	female	FAFRC, BB Yukuhashi, Japan	variety	
	0023	Celeste	female	FAFRC, BB Yukuhashi, Japan	variety	
	0024	Tanikawa	female	FAFRC, BB Yukuhashi, Japan	variety	
	0025	Chugoku	female	FAFRC, BB Yukuhashi, Japan	variety	
	0026	Dreamy Sweet	female	FAFRC, BB Yukuhashi, Japan	variety	
	0027	Negronne	female	FAFRC, BB Yukuhashi, Japan	variety	
	0028	Noire de Caromb	female	FAFRC, BB Yukuhashi, Japan	variety	
	0029	Negro Largo	female	FAFRC, BB Yukuhashi, Japan	variety	
	0030	Banane	female	FAFRC, BB Yukuhashi, Japan	variety	
	0031	Palmata	male	FAFRC, BB Yukuhashi, Japan	variety	*F. palmata*
	0032	Bellone	female	FAFRC, BB Yukuhashi, Japan	variety	
	0033	Viollette de Sollies	female	FAFRC, BB Yukuhashi, Japan	variety	
	0034	Viollette de Dauphine	female	FAFRC, BB Yukuhashi, Japan	variety	
	0035	Burjassotte Greece	female	FAFRC, BB Yukuhashi, Japan	variety	
	0036	Brunswick	female	FAFRC, BB Yukuhashi, Japan	variety	
	0037	Bodyul	female	FAFRC, BB Yukuhashi, Japan	variety	
	0038	Boldido Negra	female	FAFRC, BB Yukuhashi, Japan	variety	
	0039	White Adriatic	female	FAFRC, BB Yukuhashi, Japan	variety	
	0040	White	female	FAFRC, BB Yukuhashi, Japan	variety	
	0041	Jordan	female	FAFRC, BB Yukuhashi, Japan	variety	
	0042	Lisa	female	FAFRC, BB Yukuhashi, Japan	variety	
	0043	Royal Vineyard	female	FAFRC, BB Yukuhashi, Japan	variety	
	0044	91031	female	FAFRC, BB Yukuhashi, Japan	variety	
	0045	91032	female	FAFRC, BB Yukuhashi, Japan	variety	
	0046	91033	female	FAFRC, BB Yukuhashi, Japan	variety	
	0047	Violette de Bordeaux	female	FAFRC, BB Yukuhashi, Japan	variety	
	0048	Black Fig	female	FAFRC, BB Yukuhashi, Japan	variety	
	0049	Brown Turkey	female	FAFRC, BB Yukuhashi, Japan	variety	
	0050	Cola Branchire	female	FAFRC, BB Yukuhashi, Japan	variety	
	0051	Dieredo	female	FAFRC, BB Yukuhashi, Japan	variety	
	0052	Du Roi	female	FAFRC, BB Yukuhashi, Japan	variety	
	0053	HAA9	female	FAFRC, BB Yukuhashi, Japan	variety	
	0054	Kalmon	female	FAFRC, BB Yukuhashi, Japan	variety	
	0055	Permicula Mitis	female	FAFRC, BB Yukuhashi, Japan	variety	
	0056	San Pietro	female	FAFRC, BB Yukuhashi, Japan	variety	
	0057	Sari Lop	female	FAFRC, BB Yukuhashi, Japan	variety	
	0058	Standord	female	FAFRC, BB Yukuhashi, Japan	variety	
	0059	Verbena	female	FAFRC, BB Yukuhashi, Japan	variety	
	0060	Zidi	female	FAFRC, BB Yukuhashi, Japan	variety	

Supplementary Table S9. Material list for GWAS of sex determination (Continued)

1001	VC-106	bisexual	FAFRC, BB Yukuhashi, Japan	parental line
1002	VC-180	bisexual	FAFRC, BB Yukuhashi, Japan	parental line
1003	H238-107	bisexual	FAFRC, BB Yukuhashi, Japan	parental line
1004	M238-1	bisexual	FAFRC, BB Yukuhashi, Japan	parental line
1006	VS238-53	bisexual	FAFRC, BB Yukuhashi, Japan	parental line
1008	V13-37	bisexual	FAFRC, BB Yukuhashi, Japan	parental line
1009	CH-27	bisexual	FAFRC, BB Yukuhashi, Japan	parental line
1010	CM-15	bisexual	FAFRC, BB Yukuhashi, Japan	parental line
1011	VHV-57	bisexual	FAFRC, BB Yukuhashi, Japan	parental line
1012	VHV-6	bisexual	FAFRC, BB Yukuhashi, Japan	parental line
1013	H180-156	female	FAFRC, BB Yukuhashi, Japan	parental line
1014	H180-2	female	FAFRC, BB Yukuhashi, Japan	parental line
1017	M106-238	bisexual	FAFRC, BB Yukuhashi, Japan	parental line
1018	G180-73	female	FAFRC, BB Yukuhashi, Japan	parental line
1021	VD238-84	female	FAFRC, BB Yukuhashi, Japan	parental line
1022	VD238-96	female	FAFRC, BB Yukuhashi, Japan	parental line
1023	MH84	female	FAFRC, BB Yukuhashi, Japan	parental line
1024	G73-51	female	FAFRC, BB Yukuhashi, Japan	parental line
1025	H156-2	female	FAFRC, BB Yukuhashi, Japan	parental line
1026	H156-42	female	FAFRC, BB Yukuhashi, Japan	parental line
1027	V13-79	female	FAFRC, BB Yukuhashi, Japan	parental line
1028	H156-57	bisexual	FAFRC, BB Yukuhashi, Japan	parental line
1029	H156-59	female	FAFRC, BB Yukuhashi, Japan	parental line
1031	NVD30	female	FAFRC, BB Yukuhashi, Japan	parental line
1032	NVD6	female	FAFRC, BB Yukuhashi, Japan	parental line
1033	HIG24	female	FAFRC, BB Yukuhashi, Japan	parental line
1034	VDV35	female	FAFRC, BB Yukuhashi, Japan	parental line
1035	VHV3	female	FAFRC, BB Yukuhashi, Japan	parental line
1036	CHEO-15	unknown	FAFRC, BB Yukuhashi, Japan	parental line
1037	HICHI13-8	unknown	FAFRC, BB Yukuhashi, Japan	parental line
1038	TV-20	female	FAFRC, BB Yukuhashi, Japan	parental line
1040	NVD6CH-33	female	FAFRC, BB Yukuhashi, Japan	parental line
1041	NVD6G-11	female	FAFRC, BB Yukuhashi, Japan	parental line
1042	NVD6G-23	female	FAFRC, BB Yukuhashi, Japan	parental line
1043	NVD6G-31	female	FAFRC, BB Yukuhashi, Japan	parental line
1044	NVD6G-39	female	FAFRC, BB Yukuhashi, Japan	parental line
1045	NVD6G-44	female	FAFRC, BB Yukuhashi, Japan	parental line
1046	NVD6VHV-17	female	FAFRC, BB Yukuhashi, Japan	parental line
1047	NVD6VHV-27	female	FAFRC, BB Yukuhashi, Japan	parental line
1048	NVD6VHV-34	female	FAFRC, BB Yukuhashi, Japan	parental line
1049	NVD6VHV-43	female	FAFRC, BB Yukuhashi, Japan	parental line
1050	NVD6VHV-46	female	FAFRC, BB Yukuhashi, Japan	parental line
1051	NVD6VHV-48	female	FAFRC, BB Yukuhashi, Japan	parental line
1052	NVD6VHV-50	female	FAFRC, BB Yukuhashi, Japan	parental line
1053	NVD6VS-12	female	FAFRC, BB Yukuhashi, Japan	parental line
1054	NVD6VS-15	female	FAFRC, BB Yukuhashi, Japan	parental line
1055	NVD6VS-2	female	FAFRC, BB Yukuhashi, Japan	parental line
1056	NVD6VS-29	female	FAFRC, BB Yukuhashi, Japan	parental line
1057	NVD6CH-46	unknown	FAFRC, BB Yukuhashi, Japan	parental line
1058	NH46	female	FAFRC, BB Yukuhashi, Japan	parental line
1059	VD238-83	bisexual	FAFRC, BB Yukuhashi, Japan	parental line
1060	V13-73	female	FAFRC, BB Yukuhashi, Japan	parental line
1061	TNG21	female	FAFRC, BB Yukuhashi, Japan	parental line
1062	TNG23	female	FAFRC, BB Yukuhashi, Japan	parental line
1063	TNG25	female	FAFRC, BB Yukuhashi, Japan	parental line
1064	VC-119	bisexual	FAFRC, BB Yukuhashi, Japan	parental line
1065	NVD27	bisexual	FAFRC, BB Yukuhashi, Japan	parental line
1066	CM-1	bisexual	FAFRC, BB Yukuhashi, Japan	parental line
1067	G180-8	bisexual	FAFRC, BB Yukuhashi, Japan	parental line
1068	NG-13	female	FAFRC, BB Yukuhashi, Japan	parental line
1069	NG-60	bisexual	FAFRC, BB Yukuhashi, Japan	parental line
1076	H70NG60	bisexual	FAFRC, BB Yukuhashi, Japan	parental line

Supplementary Table S10. The RAD-seq alignment results of 122 cultivars/parent lines for GWAS.

ID	raw read	input	pair aligned	single aligned	total aligned	alinment rate
0001	2,129,684	2,093,842	1,458,958	99,905	1,558,863	74.45%
0002	2,079,030	2,048,347	1,353,944	139,853	1,493,797	72.93%
0003	2,092,026	2,060,860	1,333,822	118,844	1,452,666	70.49%
0004	2,451,306	2,414,685	1,706,406	137,290	1,843,696	76.35%
0005	2,098,674	2,068,958	1,437,560	130,930	1,568,490	75.81%
0006	1,873,528	1,843,347	1,259,622	111,271	1,370,893	74.37%
0007	2,232,642	2,193,649	1,532,924	132,607	1,665,531	75.93%
0008	2,084,706	2,054,171	1,342,990	112,458	1,455,448	70.85%
0009	2,069,074	2,035,818	1,300,424	136,279	1,436,703	70.57%
0010	2,301,182	2,263,432	1,603,348	134,481	1,737,829	76.78%
0011	2,151,004	2,118,270	1,460,444	133,964	1,594,408	75.27%
0012	2,472,494	2,435,635	1,622,656	159,072	1,781,728	73.15%
0013	2,381,834	2,349,560	1,554,382	157,713	1,712,095	72.87%
0014	2,261,398	2,226,981	1,509,816	144,267	1,654,083	74.27%
0015	2,520,478	2,482,998	1,654,860	162,519	1,817,379	73.19%
0016	2,107,988	2,074,702	1,470,180	104,944	1,575,124	75.92%
0017	2,509,920	2,470,471	1,675,032	141,210	1,816,242	73.52%
0018	2,536,990	2,501,575	1,702,386	168,310	1,870,696	74.78%
0019	2,330,204	2,298,683	1,569,426	151,374	1,720,800	74.86%
0020	2,536,698	2,499,902	1,620,628	184,840	1,805,468	72.22%
0021	2,629,716	2,593,679	1,754,490	171,404	1,925,894	74.25%
0022	2,540,608	2,502,233	1,664,630	176,445	1,841,075	73.58%
0023	2,766,024	2,723,459	1,855,670	178,885	2,034,555	74.70%
0024	2,656,480	2,617,997	1,832,592	155,688	1,988,280	75.95%
0025	2,482,656	2,447,076	1,634,156	168,224	1,802,380	73.65%
0026	2,464,066	2,427,815	1,606,544	163,497	1,770,041	72.91%
0027	2,258,710	2,225,936	1,516,592	157,282	1,673,874	75.20%
0028	2,324,698	2,289,213	1,465,660	168,272	1,633,932	71.38%
0029	2,558,536	2,521,689	1,704,644	166,904	1,871,548	74.22%
0030	2,170,968	2,140,694	1,431,060	162,828	1,593,888	74.46%
0031	2,554,794	2,517,607	1,756,924	168,690	1,925,614	76.49%
0032	2,426,830	2,389,982	1,637,852	140,384	1,778,236	74.40%
0033	2,370,626	2,334,262	1,446,960	150,347	1,597,307	68.43%
0034	2,456,130	2,421,336	1,631,560	166,730	1,798,290	74.27%
0035	2,305,224	2,273,219	1,497,706	164,332	1,662,038	73.11%
0036	2,390,940	2,356,465	1,578,608	166,191	1,744,799	74.04%
0037	2,456,068	2,422,015	1,612,788	168,727	1,781,515	73.56%
0038	2,505,694	2,471,307	1,589,840	169,833	1,759,673	71.20%
0039	2,609,546	2,572,294	1,564,982	188,581	1,753,563	68.17%
0040	2,491,880	2,454,321	1,571,056	168,056	1,739,112	70.86%
0041	2,731,638	2,689,145	1,801,058	183,942	1,985,000	73.82%
0042	2,347,082	2,312,170	1,536,240	162,529	1,698,769	73.47%
0043	2,421,290	2,386,434	1,590,644	163,086	1,753,730	73.49%
0044	2,570,592	2,533,013	1,669,258	188,554	1,857,812	73.34%
0045	2,696,624	2,658,267	1,686,154	200,909	1,887,063	70.99%
0046	2,534,878	2,499,976	1,685,946	167,093	1,853,039	74.12%
0047	2,794,330	2,751,128	1,822,172	189,318	2,011,490	73.12%
0048	2,545,142	2,507,465	1,665,798	172,927	1,838,725	73.33%
0049	2,433,584	2,399,934	1,611,134	152,131	1,763,265	73.47%
0050	2,289,462	2,258,339	1,360,662	165,005	1,525,667	67.56%
0051	2,207,150	2,178,329	1,447,292	155,870	1,603,162	73.60%
0052	2,181,298	2,151,092	1,428,660	162,303	1,590,963	73.96%
0053	2,326,256	2,296,246	1,489,126	175,983	1,665,109	72.51%
0054	2,523,752	2,489,163	1,654,122	185,002	1,839,124	73.89%
0055	2,411,630	2,378,169	1,568,340	179,549	1,747,889	73.50%
0056	2,423,076	2,389,848	1,558,256	169,423	1,727,679	72.29%
0057	2,551,144	2,512,992	1,642,920	176,884	1,819,804	72.42%
0058	2,499,134	2,462,166	1,604,302	163,797	1,768,099	71.81%
0059	2,256,208	2,225,794	1,488,728	156,908	1,645,636	73.93%
0060	2,639,348	2,601,146	1,523,892	138,564	1,662,456	63.91%

Supplementary Table S10. The RAD-seq alignment results of 122 cultivars/parent lines for GWAS (Continued)

1001	2,380,532	2,340,973	1,650,436	126,795	1,777,231	75.92%
1002	2,094,072	2,063,832	1,472,832	118,665	1,591,497	77.11%
1003	2,528,680	2,490,728	1,798,212	130,103	1,928,315	77.42%
1004	2,125,508	2,090,348	1,455,440	124,441	1,579,881	75.58%
1006	2,179,678	2,145,108	1,483,016	125,625	1,608,641	74.99%
1008	1,783,744	1,755,864	1,279,542	76,039	1,355,581	77.20%
1009	1,972,638	1,933,609	1,296,874	132,685	1,429,559	73.93%
1010	1,377,966	1,347,630	981,606	37,040	1,018,646	75.59%
1011	2,489,734	2,446,421	1,690,582	151,198	1,841,780	75.28%
1012	2,149,898	2,117,367	1,496,766	131,078	1,627,844	76.88%
1013	2,345,418	2,307,990	1,627,588	144,742	1,772,330	76.79%
1014	2,208,152	2,171,332	1,525,702	140,990	1,666,692	76.76%
1017	2,021,360	1,988,491	1,373,934	129,345	1,503,279	75.60%
1018	1,928,374	1,896,331	1,305,988	115,510	1,421,498	74.96%
1021	2,275,974	2,236,773	1,562,542	129,566	1,692,108	75.65%
1022	1,482,166	1,449,291	1,105,210	43,896	1,149,106	79.29%
1023	2,138,298	2,093,177	1,517,630	91,344	1,608,974	76.87%
1024	2,160,378	2,123,484	1,479,588	131,682	1,611,270	75.88%
1025	2,059,524	2,023,152	1,399,498	130,637	1,530,135	75.63%
1026	2,065,556	2,029,285	1,464,170	123,193	1,587,363	78.22%
1027	1,862,676	1,829,831	1,306,850	110,055	1,416,905	77.43%
1028	1,865,114	1,833,653	1,280,248	105,829	1,386,077	75.59%
1029	2,054,728	2,019,394	1,367,438	144,324	1,511,762	74.86%
1031	1,543,246	1,514,135	1,063,166	69,546	1,132,712	74.81%
1032	2,646,010	2,598,628	1,823,170	155,315	1,978,485	76.14%
1033	2,104,054	2,067,536	1,412,208	133,201	1,545,409	74.75%
1034	2,407,858	2,367,255	1,656,350	143,743	1,800,093	76.04%
1035	2,139,690	2,102,198	1,464,746	139,462	1,604,208	76.31%
1036	2,185,456	2,148,439	1,503,282	131,740	1,635,022	76.10%
1037	2,076,486	2,042,062	1,397,922	129,277	1,527,199	74.79%
1038	2,502,290	2,456,749	1,729,648	149,089	1,878,737	76.47%
1040	2,182,430	2,140,374	1,487,936	93,788	1,581,724	73.90%
1041	2,156,106	2,117,919	1,502,486	110,431	1,612,917	76.16%
1042	2,202,898	2,164,003	1,486,278	145,924	1,632,202	75.43%
1043	2,529,164	2,486,183	1,748,434	159,678	1,908,112	76.75%
1044	2,309,604	2,273,581	1,572,086	142,470	1,714,556	75.41%
1045	1,919,138	1,886,242	1,299,262	123,527	1,422,789	75.43%
1046	1,826,286	1,795,727	1,252,566	111,165	1,363,731	75.94%
1047	2,159,996	2,122,978	1,462,608	135,835	1,598,443	75.29%
1048	1,988,062	1,954,957	1,408,068	91,285	1,499,353	76.69%
1049	2,579,770	2,535,946	1,762,820	152,148	1,914,968	75.51%
1050	2,345,248	2,306,247	1,663,922	134,621	1,798,543	77.99%
1051	2,140,950	2,105,523	1,449,724	133,881	1,583,605	75.21%
1052	2,092,938	2,057,128	1,455,902	127,705	1,583,607	76.98%
1053	2,151,512	2,112,803	1,470,566	129,336	1,599,902	75.72%
1054	2,008,160	1,971,862	1,381,532	114,566	1,496,098	75.87%
1055	2,233,774	2,193,709	1,544,472	135,944	1,680,416	76.60%
1056	1,915,080	1,879,732	1,339,878	89,221	1,429,099	76.03%
1057	2,302,582	2,256,746	1,601,046	122,688	1,723,734	76.38%
1058	2,351,286	2,308,340	1,579,324	134,284	1,713,608	74.24%
1059	2,466,262	2,419,409	1,730,624	162,319	1,892,943	78.24%
1060	2,514,002	2,468,984	1,654,458	168,805	1,823,263	73.85%
1061	2,247,840	2,205,749	1,574,198	134,359	1,708,557	77.46%
1062	2,106,768	2,068,052	1,428,862	128,511	1,557,373	75.31%
1063	2,461,918	2,413,467	1,644,438	156,151	1,800,589	74.61%
1064	2,048,108	2,008,797	1,388,362	115,234	1,503,596	74.85%
1065	2,555,974	2,508,579	1,717,038	156,776	1,873,814	74.70%
1066	2,266,854	2,227,977	1,517,476	146,169	1,663,645	74.67%
1067	2,175,930	2,136,717	1,505,644	133,385	1,639,029	76.71%
1068	1,993,126	1,954,712	1,302,494	130,612	1,433,106	73.32%
1069	2,321,468	2,279,143	1,574,992	143,759	1,718,751	75.41%
1076	2,074,932	2,039,431	1,425,056	127,732	1,552,788	76.14%
Total	277,808,096	273,351,384	186,263,562	17,331,672	203,595,234	
Average	2,277,116	2,240,585	1,526,751	142,063	1,668,813	74.60%

Supplementary Table S11. SNP positon genotypes of seq000259 scaffold in GWAS materials.

ID	sexuality	seq000259 SNP position										
		8998	9255	39953	100369	100417	100426	100428	134172	134173	134177	134322
0004	bisexual	M	R	W	K	W	Y	M	A	G	A	A
0005	bisexual	M	R	W	K	W	Y	M	R	R	R	R
1001	bisexual	M	R	W	K	W	Y	M	R	R	R	R
1002	bisexual	M	R	W	K	W	Y	M	A	G	A	A
1003	bisexual	M	R	W	K	W	Y	M	R	R	R	R
1004	bisexual	M	R	W	K	A	C	C	R	R	R	R
1006	bisexual	M	R	W	K	W	Y	M	R	R	R	R
1008	bisexual	M	R	W	K	W	Y	M	R	R	R	R
1009	bisexual	M	R	W	K	W	Y	M	G	A	G	G
1010	bisexual	M	R	W	K	W	Y	M	R	R	R	R
1011	bisexual	M	R	W	K	A	C	C	A	G	A	A
1012	bisexual	M	R	W	K	W	Y	M	R	R	R	R
1017	bisexual	M	R	W	K	A	C	C	R	R	R	R
1028	bisexual	M	R	W	K	W	Y	M	A	G	A	A
1059	bisexual	M	R	W	K	W	Y	M	G	A	G	G
1064	bisexual	M	R	W	K	A	C	C	A	G	A	A
1065	bisexual	M	R	W	K	A	C	C	R	R	R	R
1066	bisexual	M	R	W	K	W	Y	M	R	R	R	R
1067	bisexual	M	R	W	K	A	C	C	R	R	R	R
1069	bisexual	M	R	W	K	W	Y	M	R	R	R	R
1076	bisexual	M	R	W	K	A	C	C	A	G	A	A
0001	female	A	A	A	G	T	T	A	G	A	G	G
0002	female	A	A	A	G	W	Y	M	R	R	R	R
0003	female	A	A	A	G	W	Y	M	R	R	R	R
0006	female	A	A	A	G	W	Y	M	R	R	R	R
0007	female	A	A	A	G	W	Y	M	R	R	R	R
0008	female	A	A	A	G	W	Y	M	G	A	G	G
0009	female	A	A	A	G	T	T	A	G	A	G	G
0010	female	A	A	A	G	W	Y	M	R	R	R	R
0011	female	A	A	A	G	T	T	A	G	R	G	G
0012	female	A	A	A	G	W	Y	M	R	R	R	R
0013	female	A	A	A	G	W	Y	M	R	R	R	R
0014	female	A	A	A	G	W	Y	M	R	R	R	R
0015	female	A	A	A	G	T	T	A	A	G	A	A
0016	female	A	A	A	G	W	Y	M	R	R	R	R
0017	female	A	A	A	G	W	Y	M	G	A	G	G
0018	female	A	A	A	G	T	T	A	G	A	G	G
0019	female	A	A	A	G	W	T	A	R	R	R	R
0020	female	A	A	A	G	W	Y	M	R	R	R	R
0021	female	A	A	A	G	W	Y	M	G	A	G	G
0022	female	A	A	A	G	W	Y	M	G	A	G	G
0023	female	A	A	A	G	T	T	A	G	A	G	G
0024	female	A	A	A	G	T	T	A	G	A	G	G
0025	female	A	A	A	G	T	T	A	R	R	R	R
0026	female	A	A	A	G	W	Y	M	G	R	G	G
0027	female	A	A	A	G	W	Y	M	G	A	G	G
0028	female	A	A	A	G	T	T	A	A	G	A	A
0029	female	A	A	A	G	W	Y	M	G	A	G	G
0030	female	A	A	A	G	T	T	A	R	R	R	R
0031	female	M	A	A	G	W	Y	M	G	G	G	G
0032	female	A	A	A	G	T	T	A	R	R	R	R
0033	female	A	A	A	G	T	T	A	R	R	R	R
0034	female	A	A	A	G	W	Y	M	R	R	R	R
0035	female	A	A	A	G	T	T	A	R	R	R	R
0036	female	A	A	A	G	W	Y	M	G	A	G	G
0037	female	A	A	A	G	W	Y	M	G	A	G	G
0038	female	A	A	A	G	W	Y	M	G	A	G	G
0039	female	A	A	A	G	W	Y	M	R	R	R	R
0040	female	A	A	A	G	T	T	A	G	R	G	G

Supplementary Table S11. SNP positon genotypes of seq000259 scaffold in GWAS materials. (Continued)

0041	female	A	A	A	G	T	T	A	R	R	R	R
0042	female	A	A	A	G	W	Y	M	R	R	R	R
0043	female	A	A	A	G	W	Y	M	G	A	G	G
0044	female	A	A	A	G	T	T	A	G	A	G	G
0045	female	A	A	A	G	T	T	A	A	G	A	A
0046	female	A	A	A	G	T	T	A	A	G	A	A
0047	female	A	A	A	G	T	T	A	R	R	R	R
0048	female	A	A	A	G	W	Y	M	G	A	G	G
0049	female	A	A	A	G	T	T	A	R	R	R	R
0050	female	A	A	A	G	T	T	A	R	R	R	R
0051	female	A	A	A	G	T	T	A	R	R	R	R
0052	female	A	A	A	G	W	Y	M	G	A	G	G
0053	female	A	A	A	G	T	T	A	G	A	G	G
0054	female	A	A	A	G	W	Y	M	R	R	R	R
0055	female	A	A	A	G	T	T	A	R	R	R	R
0056	female	A	A	A	G	T	T	A	G	R	G	G
0057	female	A	A	A	G	T	T	A	G	A	G	G
0058	female	A	A	A	G	T	T	A	G	A	G	G
0059	female	A	A	A	G	W	Y	M	R	R	R	R
0060	female	A	A	A	G	T	T	A	G	A	G	G
1013	female	A	A	A	G	T	T	A	R	R	R	R
1014	female	A	A	A	G	T	T	A	R	R	R	R
1018	female	A	A	A	G	T	T	A	R	R	R	R
1021	female	A	A	A	G	W	Y	M	R	R	R	R
1022	female	A	A	A	G	A	C	C	G	A	G	G
1023	female	A	A	A	G	W	Y	M	R	R	R	R
1024	female	A	A	A	G	W	Y	M	A	G	A	A
1025	female	A	A	A	G	W	Y	M	R	R	R	R
1026	female	A	A	A	G	W	Y	M	A	G	A	A
1027	female	A	A	A	G	W	Y	M	R	R	R	R
1029	female	A	A	A	G	W	Y	M	R	R	R	R
1031	female	A	A	A	G	W	Y	M	R	R	R	R
1032	female	A	A	A	G	T	T	A	R	R	R	R
1033	female	A	A	A	G	T	T	A	G	A	G	G
1034	female	A	A	A	G	W	Y	M	A	G	A	A
1035	female	A	A	A	G	T	T	A	G	A	G	G
1038	female	A	A	A	G	T	T	A	G	A	G	G
1040	female	A	A	A	G	T	T	A	R	R	R	R
1041	female	A	A	A	G	T	T	A	R	R	R	R
1042	female	A	A	A	G	T	T	A	R	R	R	R
1043	female	A	A	A	G	T	T	A	G	A	G	G
1044	female	A	A	A	G	T	T	A	R	R	R	R
1045	female	A	A	A	G	T	T	A	A	G	A	A
1046	female	A	A	A	G	T	T	A	R	R	R	R
1047	female	A	A	A	G	T	T	A	R	R	R	R
1048	female	A	A	A	G	T	T	A	R	R	R	R
1049	female	A	A	A	G	T	T	A	R	R	R	R
1050	female	A	A	A	G	T	T	A	A	G	A	A
1051	female	A	A	A	G	T	T	A	A	G	A	A
1052	female	A	A	A	G	T	T	A	R	R	R	R
1053	female	A	A	A	G	T	T	A	R	R	R	R
1054	female	A	A	A	G	T	T	A	A	G	A	A
1055	female	A	A	A	G	T	T	A	R	R	R	R
1056	female	A	A	A	G	T	T	A	G	A	G	G
1058	female	A	A	A	G	T	T	A	G	A	G	G
1060	female	A	A	A	G	W	Y	M	R	R	R	R
1061	female	A	A	A	G	T	T	A	R	R	R	R
1062	female	A	A	A	G	W	Y	M	R	R	R	R
1063	female	A	A	A	G	T	T	A	R	R	R	R
1068	female	A	A	A	G	W	Y	M	G	A	G	G
1036	unknown	A	A	A	G	T	T	A	G	A	G	G
1037	unknown	M	R	W	K	W	Y	M	R	R	R	R
1057	unknown	M	R	W	K	W	Y	M	R	R	R	R

Supplementary Table S12. Male material list for SNPs sequencing.

species	ID	variety/accession name	sexuality	sampling location	annotation
F. carica	1001	VC-106	male	FAFRC, BB Yukuhashi, Japan	breeding parent
	1002	VC-180	male	FAFRC, BB Yukuhashi, Japan	breeding parent
	1003	H238-107	male	FAFRC, BB Yukuhashi, Japan	breeding parent
	1004	M238-1	male	FAFRC, BB Yukuhashi, Japan	breeding parent
	1006	VS238-53	male	FAFRC, BB Yukuhashi, Japan	breeding parent
	1008	V13-37	male	FAFRC, BB Yukuhashi, Japan	breeding parent
	1009	CH-27	male	FAFRC, BB Yukuhashi, Japan	breeding parent
	1010	CM-15	male	FAFRC, BB Yukuhashi, Japan	breeding parent
	1011	VHV-57	male	FAFRC, BB Yukuhashi, Japan	breeding parent
	1012	VHV-6	male	FAFRC, BB Yukuhashi, Japan	breeding parent
	1059	VD238-83	male	FAFRC, BB Yukuhashi, Japan	breeding parent
	1064	VC-119	male	FAFRC, BB Yukuhashi, Japan	breeding parent
	1065	NVD27	male	FAFRC, BB Yukuhashi, Japan	breeding parent
	1066	CM-1	male	FAFRC, BB Yukuhashi, Japan	breeding parent
	1067	G180-8	male	FAFRC, BB Yukuhashi, Japan	breeding parent
	1069	NG-60	male	FAFRC, BB Yukuhashi, Japan	breeding parent
	1076	H70NG60	male	FAFRC, BB Yukuhashi, Japan	breeding parent
	0031	Palmata	male	FAFRC, BB Yukuhashi, Japan	variety

Supplementary Table S13. Sex associated SNP panels in the 18 male lines.

					ID																
Scaffold	Position	REF	SNP	ALT	1001	1002	1003	1004	1006	1008	1009	1010	1011	1012	1059	1064	1065	1066	1067	1069	0031
seq000259	430	C	Y	T	C	C	C	C	C	C	C	C	C	C	C	C	C	C	C	C	C
seq000259	433	C	Y	T	C	C	C	C	C	C	C	C	C	C	C	C	C	C	C	C	C
seq000259	543	T	Y	C	T	T	T	T	T	T	T	T	T	T	T	T	T	T	T	T	T
seq000259	9876	T	Y	C	Y	Y	Y	Y	Y	Y	Y	Y	Y	Y	Y	Y	Y	Y	Y	Y	Y
seq000259	9900	C	Y	T	Y	Y	Y	Y	Y	Y	Y	Y	Y	Y	Y	Y	Y	Y	Y	Y	C
seq000259	12314	C	Y	T	Y	Y	Y	Y	Y	Y	Y	Y	Y	Y	Y	Y	Y	Y	Y	Y	Y
seq000259	12722	C	S	G	S	S	S	S	S	S	S	S	S	S	S	S	S	S	S	S	C
seq000259	12743	A	R	G	R	R	R	R	R	R	R	R	R	R	R	R	R	R	R	R	A
seq000259	16247	C	M	A	C	C	C	C	C	C	C	C	C	C	C	C	C	C	C	C	na
seq000259	16262	G	R	A	G	G	G	G	G	G	G	G	G	G	G	G	G	G	G	G	na
seq000259	16332	G	R	A	G	G	G	G	G	G	G	G	G	G	G	G	G	G	G	G	G
seq000259	16431	G	K	T	G	G	G	G	G	G	G	G	G	G	G	G	G	G	G	G	G
seq000259	16644	A	R	G	A	A	A	A	A	A	A	A	A	A	A	A	A	A	A	A	na
seq000259	33672	G	R	A	R	R	R	R	R	R	R	R	R	R	R	R	R	R	R	R	G
seq000259	33676	C	Y	T	Y	Y	Y	Y	Y	Y	Y	Y	Y	Y	Y	Y	Y	Y	Y	Y	C
seq000259	33702	G	R	A	R	R	R	R	R	R	R	R	R	R	R	R	R	R	R	R	G
seq000259	33801	G	S	C	S	S	S	S	S	S	S	S	S	S	S	S	S	S	S	S	C
seq000259	33951	A	R	G	R	R	R	R	R	R	R	R	R	R	R	R	R	R	R	R	G
seq000259	36750	G	K	T	K	K	K	K	K	K	K	K	K	K	K	K	K	K	K	K	G
seq000259	36821	T	Y	C	Y	Y	Y	Y	Y	Y	Y	Y	Y	Y	Y	Y	Y	Y	Y	Y	C
seq000259	36935	C	Y	T	Y	Y	Y	Y	Y	Y	Y	Y	Y	Y	Y	Y	Y	Y	Y	Y	C
seq000259	37211	G	R	A	R	R	R	R	R	R	R	R	R	R	R	R	R	R	R	R	G
seq000259	37307	C	M	A	M	M	M	M	M	M	M	M	M	M	M	M	M	M	M	M	C
seq000259	38048	A	W	T	W	W	W	W	W	W	W	W	W	W	W	W	W	W	W	W	A
seq000259	39084	A	W	T	W	W	W	W	W	W	W	W	W	W	W	W	W	W	W	W	A
seq000259	39144	C	M	A	M	M	M	M	M	M	M	M	M	M	M	M	M	M	M	M	C
seq000259	41088	C	Y	T	Y	Y	Y	Y	Y	Y	Y	Y	Y	Y	Y	Y	Y	Y	Y	Y	C
seq000259	45751	A	Del	-	na	na	na	na	na	na	na	Del	Del	na	na	na	na	na	na	na	A
seq000259	45832	G	R	A	na	na	na	na	na	na	na	R	R	na	na	na	na	na	na	na	G
seq000259	45840	A	R	G	na	na	na	na	na	na	na	R	R	na	na	na	na	na	na	na	G
seq000259	64179	A	W	T	W	W	W	W	W	W	W	W	W	W	W	W	W	W	W	W	T
seq000259	65387	G	R	A	R	R	R	R	R	R	R	R	R	R	R	R	R	R	R	R	G
seq000259	67378	G	R	A	na	R	R	A	na	R	R	R	A	na	R	A	A	R	A	na	G
seq000259	68029	G	K	T	K	K	K	K	K	K	K	K	K	K	K	K	K	K	K	K	G
seq000259	68033	C	M	A	na	na	na	na	na	na	na	na	na	na	na	na	na	na	na	na	C
seq000259	68512	G	R	A	na	na	na	na	na	na	na	na	na	na	na	na	na	na	na	na	G
seq000259	68583	G	R	A	na	na	na	na	na	na	na	na	na	na	na	na	na	na	na	na	G
seq000259	71075	C	Y	T	Y	Y	Y	Y	Y	Y	Y	Y	Y	Y	Y	Y	Y	Y	Y	Y	C
seq000259	100369	G	K	T	na	na	na	na	na	na	na	na	na	na	na	na	na	na	na	na	G
