# Supplementary material for: Identification of RAN1 orthologue associated with sex determination through whole genome sequencing analysis in fig (Ficus carica L.)
Source: Sci Rep. 2017 Jan 25;7:41124. doi: 10.1038/srep41124 (PMC5264649; doi:10.1038/srep41124)
Supplement: Supplementary Dataset 8 [file srep41124-s9.doc]

>HORAISHI

atggcggcgagcgtccgacacctCCAGCTCACCCAACTCTCCGCCGCCGGCGCCGGCGAC

GACGACGACTCCGGCGACCTCGAGGACGTGCGGCTCCTCGACGCGTACAAGAATTCGGAG

GAGAACGATGAGGGAGTTGTCGGAGAGGCGACGATGAAGAGGATTCAGGTCGGCGTCACC

GGCATGACCTGCGCGGCTTGCTCGAACTCCGTCGAAGCTGCTCTGATGAGCGTCAATGGC

GTTCTCAGGGCTTCCGTCGCTCTGCTCCAGAACAAGGCCGACGTGGTCTTCGATCCCAGA

TTGGTCAAGGATGAAGATATCAAGAATGCAATCGAGGATGCTGGGTTTGAAGCTGAGATT

CTACCTGAATCAAGTGCCATTGGAACAAAGCCTCAAGCGACCATGTTAGGGCAGTTCTCT

ATAGGTGGCATGACATGTGCGGCTTGTGTGAACTCGGTAGAAGGCATTTTACGAGATCTT

CCCGGTGTCAAAAGGGCTGTAGTTGCCTTGGCTACTTCATTAGGTGAAGTTGAGTATGAT

CCAGCTGTTATCAGTAAAGAGGATATCGTCAATGCAATTGAAGACGCTGGTTTTGAAGGA

GCCTTTGTACAGAGCAGTGAACAAGATAAGATTGTGTTGGGCGTTGCTGGCATATACAGT

GAGATGGATGTACAGATTTTAGGAGGCATACTTAGCAACTTGAAAGGGGTGAGACAATTT

CATTTTGACCGAATTTCAAGAGAACTTGAAGTTCTGTTCGATCCTGAAGTTATTCATTCA

AGATCCTTAGTTGATGGGGTTGAAGGGGGAAGTAGCGGAAGGTTTAAATTACGTGTTGCT

AATCCTTATTCAAGAATGACTTCTAAAGATGTGGAAGAAGCCTCAAACATGTTTAGACTG

TTCATCTCTAGTCTATTTCTCAGCGTTCCTGTCTTTCTCATACGAGTAGTTTGTCCACAC

ATACCACTGATTTATTCCTTATTGCTTTGGCGATGTGGGCCCTTTCAAATGGGTGACTGG

TTGAAGTGGGCGTTGGTGAGTGTTGTTCAATTTGTTGTTGGAAAGCGCTTCTACATTGCT

GCTGCAAGAGCTCTACGAAATGGTTCAACTAACATGGATGTTTTGGTTGCATTGGGGACA

TCAGCCTCCTATTTCTACTCTGTTTGTGCACTTCTATATGGTGCACTCACTGGGTTTTGG

TCCCCAACTTACTTTGAAACAAGTGCAATGCTGATTACATTTGTGCTGTTAGGAAAATAT

CTAGAGTGTCTTGCAAAGGGGAAAACATCTGACGCCATCAAAAAGTTGGTAGAACTTGCA

CCAGCAACGGCAACATTGCTTATCAAGGATAAAGATGGAAGATATCTTGGAGAAAGGGAA

ATAGATGCTCTACTAATTCAGCCCGGTGACACATTGAAAGTTTCACCGGGTGCAAAATTA

CCTGCTGATGGTGTTGTTGCTTGGGGTTCAAGTTATGTAAATGAAAGTATGGTAACTGGT

GAATCTGTACCCATTTCGAAGGAGGTCGGTTCATCAGTAATTGGAGGTACAATAAATTTA

CATGGTGCTCTACACGTACAAGCTACCAAAGTAGGTTCTGACACAGTTTTGAGCCAGATA

ATCAGTTTAGTTGAAACAGCGCAGATGTCCAAAGCTCCTATTCAGAAATTTGCTGATTTC

ATCGCTAGCATTTTTGTTCCTACAGTTGTTTTGTTGGCATTGTTGACGTTATTAGGATGG

TACGCTGCTGGAGCTCTTCGTGCTTACCCAGAAAGTTGGCTTCCAGAAAATGGAAATTAC

TTTGTTTTCGCCCTTATGTTTTCTATATCAGTTGTGGTGATCGCGTGCCCCTGTGCTCTT

GGCTTGGCAACACCAACTGCGGTCATGGTTGCAACCGGAGTCGGTGCTAACAATGGTGTG

CTGATAAAAGGGGGCGATGCCTTAGAAAGGGCTCAAAAAATAAAGTATGTGATCTTTGAT

AAAACAGGTACCCTAACTCAGGGAAAGGCCTCGGTTACAACTACAAAAGTGTTTTCGGGA

ATGGATCGTGGAGAATTCCTCAAGTTGGTGGCTTCTGCTGAGGCTAGTAGTGAACATCCA

CTGGCAAAGGCAATAGTTGCATATGCCCGTCATTTTCATTTCTTTGATGATTCTGCTACC

AAGGATGCCAAAAGCCATAGCAAAGACTCCGAAGTATCTGGTTGGCTTTTTGATGTAGCA

GAATTTNCTACTCTTCCCGGCAGAGGAGTTCAATGCTTTATTAATGAAAAACAGATTTTG

GTTGGTAATCGCAAGTTGATGACCGAAAGTGGAATCGACATCCCTGATGATGTAGAAAAA

TTTGTTGTAGAACTCGAAGAAAGTGCGAAAACAGGCATACTTGTCGCATATAACGGTAAC

TTAATTGGTGTTTTGGGGGTTGCAGACCCACTGAAAAGAGAGGCAGCTGTGGTAGTTGAG

GGCCTGAGTAAAATGGGCATCCGACCGGTCATGGTTACTGGGGACAACTGGAGGACAGCG

CAGGCTGTTGCTAAGGAGGTTGGCATTCACGATGTAAGGGCAGAGGTAATGCCTGCAGGA

AAAGCCGATGTAGTTCGTTCGTTTCATAAGGATGGAATCACGGTTGCGATGGTGGGAGAC

GGAATCAATGATTCTCCAGCTCTAGCTGCAGCAGATGTTGGCATGGCAATTGGAGCAGGG

ACGGATATTGCCATTGAAGCGGCCAACTATGTTTTGATGAGGAACAACTTGGAGGATGTG

ATCACCGCCATTGATCTCTCAAGAAAGACTTTCTCTCGAATCAGATTAAATTATGTGTTT

GCCATGGCTTACAATGTCATAGCAATCCCTGTTGCTGCCGGAGTTTTCTTTCCGTCGTTG

GGGATTCAGTTGCCGCCATGGGCGGCTGGTGCATGCATGGCTTTGTCTTCTGTTAGTGTT

GTATGTTCTTCTCTATTACTTAGGAGATACAGAAAACCAAGgctgaccactatactagaa

ataa

>Caprifig6085

atggcggcgagcgtccgacacctCCAGCTCACCCAACTCTCCGCCGCCGGCGCCGGCGAC

GACGACGACTCCGGCGACCTCGAGGACGTGCGGCTCCTCGACGCGTACAAGAATTCGGAG

GAGAACGATGAGGGAGTTGTCGGAGAGGCGACGATGAAGAGGATTCAGGTCGGCGTCACC

GGCATGACCTGCGCGGCTTGCTCGAACTCCGTCGAAGCTGCTCTGATGAGCGTCAATGGC

GTTCTCAGGGCTTCCGTCGCTCTGCTCCAGAACAAGGCCGACGTGGTCTTCGATCCCAGA

TTGGTCAAGGATGAAGATATCAAGAATGCAATCGAGGATGCTGGGTTTGAAGCTGAGATT

CTACCTGAATCAAGTGCCATTGGAACAAAGCCTCAAGCGACCAYGTTAGGGCAGTTCTCT

ATAGSTGGYATGACATGTGCGGCTTGTGTGAACTCGGTAGAAGGCATTTTACGAGATCTT

CCCGGTGTCAAAAGGGCTGTMGTTGCCTTGGCTACTTCATTAGGTGAAGTTGAGTATGAT

CCAGCTGTTATCAGTAAAGAGGATATCGTCAATGCAATTGAAGACGCTGGTTTTGAAGGA

GCCTTTGTACAGAGCAGTGAACAAGATAAGATTGTGTTGGGCGTTGCTGGCATATACAGT

GAGATGGATGTACAGATTTTAGGAGGCATACTTAGCAACTTGAAAGGGGTGAGACAATTT

CATTTTGACCGAATTTCAAGAGAACTTGAAGTTCTGTTYGATCCTGAAGTTATTCATTCA

AGATCCTTAGTTGATGGGGTTGAAGGGGGAAGTAGCGGAAGGTTTAAATTACRTGTTGCT

AATCCTTATTCAAGAATGACTTCTAAAGATGTGGAAGAAGCCTCAAACATGTTTAGACTG

TTCATCTCTAGTCTATTTCTCAGCGTTCCTGTCTTTCTCATACGAGTAGTTTGTCCACAC

ATACCACTGATTTATTCCTTATTGCTTTGGCGATGTGGGCCCTTTCAAATGGGTGACTGG

TTGAAGTGGGCGTTGGTGAGTGTYGTTCAATTTGTTGTTGGAAAGCGCTTCTACATTGCT

GCTGCAAGAGCTCTACGAAATGGTTCAACTAACATGGATGTTTTGGTTGCATTGGGGACA

TCAGCCTCCTATTTCTACTCTGTTTGTGCACTTCTATATGGTGCACTCACTGGGTTTTGG

TCCCCAACTTACTTTGAAACAAGTGCAATGCTGATTACATTTGTGCTGTTAGGAAAATAT

CTAGAGTGTCTTGCAAAGGGGAAAACATCTGACGCCATCAAAAAGTTGGTAGAACTTGCA

CCAGCAACGGCAACATTGCTTATCAAGGATAAAGATGGAAGATATCTTGGAGAAAGGGAA

ATAGATGCTCTACTAATTCAGCCCGGTGACACATTGAAAGTTTCACCGGGTGCAAAATTA

CCTGCTGATGGTGTTGTTGCTTGGGGTTCAAGTTATGTAAATGAAAGTATGGTAACTGGT

GAATCTGTACCCATTTCGAAGGAGGTCGGTTCATCAGTAATTGGAGGTACAATAAATTTA

CATGGTGCTCTACACGTACAAGCTACCAAAGTAGGTTCTGACACAGTTTTGAGCCAGATA

ATCAGTTTAGTTGAAACAGCGCAGATGTCCAAAGCTCCTATTCAGAAATTTGCTGATTTC

ATCGCTAGCATTTTTGTTCCTACAGTTGTTTTGTTGGCATTGTTGACGTTATTAGGATGG

TACGCTGCTGGAGCTCTTCGTGCTTACCCAGAAAGTTGGCTTCCAGAAAATGGAAATTAC

TTTGTTTTCGCCCTTATGTTTTCTATATCAGTTGTGGTGATYGCGTGCCCCTGTGCTCTT

GGCTTGGCAACACCAACTGCGGTCATGGTTGCAACAGGAGTCGGTGCTAACAATGGTGTG

CTGATAAAAGGGGGCGATGCCTTAGAAAGGGCTCAAAAAATAAAGTATGTGATCTTTGAT

AAAACAGGTACCCTAACTCAGGGAAAGGCCTCGGTTACAACTACAAAAGTGTTTTCGGGA

ATGGATCGTGGAGAATTCCTCAAGTTGGTGGCTTCTGCTGAGGCTAGTAGTGAACATCCA

CTGGCAAAGGCAATAGTTGCATATGCCCGTCATTTTCATTTCTTTRATGATTCTGCTACC

AAGGATGCCRAAAGCCATAGCAAAGACTCCRAAGTATCTGGTTGGCTTTTTGATGTAGCA

GAATTTTCTACTCTTCCCGGCAGAGGAGTTCAATGCTTTATTAATGAAAAACAGATTTTG

GTTGGTAATCGCAAGTTGATGACCGAAAGTGGAATCGACATCCCTGATGATGTAGAAAAA

TTTGTTGTAGAACTCGAAGAAAGTGCGAAAACAGGCATACTTGTCGCATATAACGGTAAC

TTAATTGGTGTTTTGGGGGTTGCAGACCCACTGAAAAGAGAGGCAGCTGTGGTAGTTGAG

GGCCTGAGTAAAATGGGCATCCGACCGGTCATGGTTACTGGGGACAACTGGAGGACAGCG

CAGGCTGTTGCTAAGGAGGTTGGCATTCACGATGTAAGGGCAGAGGTAATGCCTGCAGGA

AAAGCCGATGTAGTTCGTTCGTTTCATAAGGATGGAATCACRGTTGCGATGGTGGGAGAC

GGAATCAATGATTCTCCAGCTCTAGCTGCAGCAGATGTTGGCATGGCAATTGGAGCAGGG

ACGGATATTGCCATTGAAGCGGCCAACTATGTTTTGATGAGGAACAACTTGGAGGATGTG

ATCACCGCCATTGATCTCTCAAGAAAGACKTTCTCTCGAATCAGATTAAATTATGTGTTT

GCCATGGCTTACAATGTCATAGCAATCCCTGTTGCTGCCGGAGTTTTCTTTCCGTCGTTG

GGGATTCAGTTGCCGCCATGGGCGGCTGGTGCATGCATGGCTTTGTCTTCTGTTAGTGTT

GTATGTTCTTCTCTATTACTTAGGAGATACAGAAAACCAAGgctgaccactatactagaa

ataa

>s00259g14131.CDS

ATGGCGGCGAGCGTCCGACACCTCCAGCTCACCCAACTCTCCGCCGCCGGCGCCGGCGAC

GACGACGACTCCGGCGACCTCGAGGACGTGCGGCTCCTCGACGCGTACAAGAATTCGGAG

GAGAACGATGAGGGAGTTGTCGGAGAGGCGACGATGAAGAGGATTCAGGTCGGCGTCACC

GGCATGACCTGCGCGGCTTGCTCGAACTCCGTCGAAGCTGCTCTGATGAGCGTCAATGGC

GTTCTCAGGGCTTCCGTCGCTCTGCTCCAGAACAAGGCCGACGTGGTCTTCGATCCCAGA

TTGGTCAAGGATGAAGATATCAAGAATGCAATCGAGGATGCTGGGTTTGAAGCTGAGATT

CTACCTGAATCAAGTGCCATTGGAACAAAGCCTCAAGCGACCATGTTAGGGCAGTTCTCT

ATAGGTGGCATGACATGTGCGGCTTGTGTGAACTCGGTAGAAGGCATTTTACGAGATCTT

CCCGGTGTCAAAAGGGCTGTAGTTGCCTTGGCTACTTCATTAGGTGAAGTTGAGTATGAT

CCAGCTGTTATCAGTAAAGAGGATATCGTCAATGCAATTGAAGACGCTGGTTTTGAAGGA

GCCTTTGTACAGAGCAGTGAACAAGATAAGATTGTGTTGGGCGTTGCTGGCATATACAGT

GAGATGGATGTACAGATTTTAGGAGGCATACTTAGCAACTTGAAAGGGGTGAGACAATTT

CATTTTGACCGAATTTCAAGAGAACTTGAAGTTCTGTTCGATCCTGAAGTTATTCATTCA

AGATCCTTAGTTGATGGGGTTGAAGGGGGAAGTAGCGGAAGGTTTAAATTACGTGTTGCT

AATCCTTATTCAAGAATGACTTCTAAAGATGTGGAAGAAGCCTCAAACATGTTTAGACTG

TTCATCTCTAGTCTATTTCTCAGCGTTCCTGTCTTTCTCATACGAGTAGTTTGTCCACAC

ATACCACTGATTTATTCCTTATTGCTTTGGCGATGTGGGCCCTTTCAAATGGGTGACTGG

TTGAAGTGGGCGTTGGTGAGTGTTGTTCAATTTGTTGTTGGAAAGCGCTTCTACATTGCT

GCTGCAAGAGCTCTACGAAATGGTTCAACTAACATGGATGTTTTGGTTGCATTGGGGACA

TCAGCCTCCTATTTCTACTCTGTTTGTGCACTTCTATATGGTGCACTCACTGGGTTTTGG

TCCCCAACTTACTTTGAAACAAGTGCAATGCTGATTACATTTGTGCTGTTAGGAAAATAT

CTAGAGTGTCTTGCAAAGGGGAAAACATCTGACGCCATCAAAAAGTTGGTAGAACTTGCA

CCAGCAACGGCAACATTGCTTATCAAGGATAAAGATGGAAGATATCTTGGAGAAAGGGAA

ATAGATGCTCTACTAATTCAGCCCGGTGACACATTGAAAGTTTCACCGGGTGCAAAATTA

CCTGCTGATGGTGTTGTTGCTTGGGGTTCAAGTTATGTAAATGAAAGTATGGTAACTGGT

GAATCTGTACCCATTTCGAAGGAGGTCGGTTCATCAGTAATTGGAGGTACAATAAATTTA

CATGGTGCTCTACACGTACAAGCTACCAAAGTAGGTTCTGACACAGTTTTGAGCCAGATA

ATCAGTTTAGTTGAAACAGCGCAGATGTCCAAAGCTCCTATTCAGAAATTTGCTGATTTC

ATCGCTAGCATTTTTGTTCCTACAGTTGTTTTGTTGGCATTGTTGACGTTATTAGGATGG

TACGCTGCTGGAGCTCTTCGTGCTTACCCAGAAAGTTGGCTTCCAGAAAATGGAAATTAC

TTTGTTTTCGCCCTTATGTTTTCTATATCAGTTGTGGTGATCGCGTGCCCCTGTGCTCTT

GGCTTGGCAACACCAACTGCGGTCATGGTTGCAACCGGAGTCGGTGCTAACAATGGTGTG

CTGATAAAAGGGGGCGATGCCTTAGAAAGGGCTCAAAAAATAAAGTATGTGATCTTTGAT

AAAACAGGTACCCTAACTCAGGGAAAGGCCTCGGTTACAACTACAAAAGTGTTTTCGGGA

ATGGATCGTGGAGAATTCCTCAAGTTGGTGGCTTCTGCTGAGGCTAGTAGTGAACATCCA

CTGGCAAAGGCAATAGTTGCATATGCCCGTCATTTTCATTTCTTTGATGATTCTGCTACC

AAGGATGCCAAAAGCCATAGCAAAGACTCCGAAGTATCTGGTTGGCTTTTTGATATTTTG

GTTGGTAATCGCAAGTTGATGACCGAAAGTGGAATCGACATCCCTGATGATGTAGAAAAA

TTTGTTGTAGAACTCGAAGAAAGTGCGAAAACAGGCATACTTGTCGCATATAACGGTAAC

TTAATTGGTGTTTTGGGGGTTGCAGACCCACTGAAAAGAGAGGCAGCTGTGGTAGTTGAG

GGCCTGAGTAAAATGGGCATCCGACCGGTCATGGTTACTGGGGACAACTGGAGGACAGCG

CAGGCTGTTGCTAAGGAGGTTGGCATTCACGATGTAAGGGCAGAGGTAATGCCTGCAGGA

AAAGCCGATGTAGTTCGTTCGTTTCATAAGGATGGAATCACGGTTGCGATGGTGGGAGAC

GGAATCAATGATTCTCCAGCTCTAGCTGCAGCAGATGTTGGCATGGCAATTGGAGCAGGG

ACGGATATTGCCATTGAAGCGGCCAACTATGTTTTGATGAGGAACAACTTGGAGGATGTG

ATCACCGCCATTGATCTCTCAAGAAAGACTTTCTCTCGAATCAGATTAAATTATGTGTTT

GCCATGGCTTACAATGTCATAGCAATCCCTGTTGCTGCCGGAGTTTTCTTTCCGTCGTTG

GGGATTCAGTTGCCGCCATGGGCGGCTGGTGCATGCATGGCTTTGTCTTCTGTTAGTGTT

GTATGTTCTTCTCTATTACTTAGGAGATACAGAAAACCAAGGCTGACCACTATACTAGAA

ATAACTGTAGAATAG

Primer sequences

>RAN1_seqf2

AACTCGGTAGAAGGCATTTTACGAG

>RAN1_seqf3

TTGTCCACACATACCACTGATTTAT

>RAN1_seqf4

GTGTTGTTGCTTGGGGTTCAAGTTA

>RAN1_seqf5

GCTCAAAAAATAAAGTATGTGATCT

>RAN1_seqf6

GGTAGTTGAGGGCCTGAGTAAAATG

>RAN1_seqr2

CTCTGCCCTTACATCGTGAATGCCA

>RAN1_seqr3

ACTTGAGGAATTCTCCACGATCCAT

>RAN1_seqr4

CCATGTAAATTTATTGTACCTCCAA

>RAN1_seqr5

CTTTCCAACAACAAATTGAACAACA

>RAN1_seqr6

TATCCTCTTTACTGATAACAGCTGG

>FcRAN1-pRI201-F1

CACTGTTGATACATATGGCGGCGAGCGTCCGACACCT

>FcRAN1-pRI201-R1

CTATTCTACAGTTATTTCTAGTATAGTGGTCAGCCTT

CLUSTAL 2.1 multiple sequence alignment

<-14396

HORAISHI atggcggcgagcgtccgacacctCCAGCTCACCCAACTCTCCGCCGCCGGCGCCGGCGAC

Caprifig6085 atggcggcgagcgtccgacacctCCAGCTCACCCAACTCTCCGCCGCCGGCGCCGGCGAC

s00259g14131.CDS ATGGCGGCGAGCGTCCGACACCTCCAGCTCACCCAACTCTCCGCCGCCGGCGCCGGCGAC

************************************************************

HORAISHI GACGACGACTCCGGCGACCTCGAGGACGTGCGGCTCCTCGACGCGTACAAGAATTCGGAG

Caprifig6085 GACGACGACTCCGGCGACCTCGAGGACGTGCGGCTCCTCGACGCGTACAAGAATTCGGAG

s00259g14131.CDS GACGACGACTCCGGCGACCTCGAGGACGTGCGGCTCCTCGACGCGTACAAGAATTCGGAG

************************************************************

HORAISHI GAGAACGATGAGGGAGTTGTCGGAGAGGCGACGATGAAGAGGATTCAGGTCGGCGTCACC

Caprifig6085 GAGAACGATGAGGGAGTTGTCGGAGAGGCGACGATGAAGAGGATTCAGGTCGGCGTCACC

s00259g14131.CDS GAGAACGATGAGGGAGTTGTCGGAGAGGCGACGATGAAGAGGATTCAGGTCGGCGTCACC

************************************************************

HORAISHI GGCATGACCTGCGCGGCTTGCTCGAACTCCGTCGAAGCTGCTCTGATGAGCGTCAATGGC

Caprifig6085 GGCATGACCTGCGCGGCTTGCTCGAACTCCGTCGAAGCTGCTCTGATGAGCGTCAATGGC

s00259g14131.CDS GGCATGACCTGCGCGGCTTGCTCGAACTCCGTCGAAGCTGCTCTGATGAGCGTCAATGGC

************************************************************

HORAISHI GTTCTCAGGGCTTCCGTCGCTCTGCTCCAGAACAAGGCCGACGTGGTCTTCGATCCCAGA

Caprifig6085 GTTCTCAGGGCTTCCGTCGCTCTGCTCCAGAACAAGGCCGACGTGGTCTTCGATCCCAGA

s00259g14131.CDS GTTCTCAGGGCTTCCGTCGCTCTGCTCCAGAACAAGGCCGACGTGGTCTTCGATCCCAGA

************************************************************

14088-><-12837

HORAISHI TTGGTCAAGGATGAAGATATCAAGAATGCAATCGAGGATGCTGGGTTTGAAGCTGAGATT

Caprifig6085 TTGGTCAAGGATGAAGATATCAAGAATGCAATCGAGGATGCTGGGTTTGAAGCTGAGATT

s00259g14131.CDS TTGGTCAAGGATGAAGATATCAAGAATGCAATCGAGGATGCTGGGTTTGAAGCTGAGATT

************************************************************

|(12743)

HORAISHI CTACCTGAATCAAGTGCCATTGGAACAAAGCCTCAAGCGACCATGTTAGGGCAGTTCTCT

Caprifig6085 CTACCTGAATCAAGTGCCATTGGAACAAAGCCTCAAGCGACCAYGTTAGGGCAGTTCTCT

s00259g14131.CDS CTACCTGAATCAAGTGCCATTGGAACAAAGCCTCAAGCGACCATGTTAGGGCAGTTCTCT

******************************************* ****************

(12722)| |12718

HORAISHI ATAGGTGGCATGACATGTGCGGCTTGTGTGAACTCGGTAGAAGGCATTTTACGAGATCTT

Caprifig6085 ATAGSTGGYATGACATGTGCGGCTTGTGTGAACTCGGTAGAAGGCATTTTACGAGATCTT

s00259g14131.CDS ATAGGTGGCATGACATGTGCGGCTTGTGTGAACTCGGTAGAAGGCATTTTACGAGATCTT

****.*** ***************************************************

|12646

HORAISHI CCCGGTGTCAAAAGGGCTGTAGTTGCCTTGGCTACTTCATTAGGTGAAGTTGAGTATGAT

Caprifig6085 CCCGGTGTCAAAAGGGCTGTMGTTGCCTTGGCTACTTCATTAGGTGAAGTTGAGTATGAT

s00259g14131.CDS CCCGGTGTCAAAAGGGCTGTAGTTGCCTTGGCTACTTCATTAGGTGAAGTTGAGTATGAT

******************** ***************************************

HORAISHI CCAGCTGTTATCAGTAAAGAGGATATCGTCAATGCAATTGAAGACGCTGGTTTTGAAGGA

Caprifig6085 CCAGCTGTTATCAGTAAAGAGGATATCGTCAATGCAATTGAAGACGCTGGTTTTGAAGGA

s00259g14131.CDS CCAGCTGTTATCAGTAAAGAGGATATCGTCAATGCAATTGAAGACGCTGGTTTTGAAGGA

************************************************************

HORAISHI GCCTTTGTACAGAGCAGTGAACAAGATAAGATTGTGTTGGGCGTTGCTGGCATATACAGT

Caprifig6085 GCCTTTGTACAGAGCAGTGAACAAGATAAGATTGTGTTGGGCGTTGCTGGCATATACAGT

s00259g14131.CDS GCCTTTGTACAGAGCAGTGAACAAGATAAGATTGTGTTGGGCGTTGCTGGCATATACAGT

************************************************************

HORAISHI GAGATGGATGTACAGATTTTAGGAGGCATACTTAGCAACTTGAAAGGGGTGAGACAATTT

Caprifig6085 GAGATGGATGTACAGATTTTAGGAGGCATACTTAGCAACTTGAAAGGGGTGAGACAATTT

s00259g14131.CDS GAGATGGATGTACAGATTTTAGGAGGCATACTTAGCAACTTGAAAGGGGTGAGACAATTT

************************************************************

|12388

HORAISHI CATTTTGACCGAATTTCAAGAGAACTTGAAGTTCTGTTCGATCCTGAAGTTATTCATTCA

Caprifig6085 CATTTTGACCGAATTTCAAGAGAACTTGAAGTTCTGTTYGATCCTGAAGTTATTCATTCA

s00259g14131.CDS CATTTTGACCGAATTTCAAGAGAACTTGAAGTTCTGTTCGATCCTGAAGTTATTCATTCA

************************************** *********************

|[12314]

HORAISHI AGATCCTTAGTTGATGGGGTTGAAGGGGGAAGTAGCGGAAGGTTTAAATTACGTGTTGCT

Caprifig6085 AGATCCTTAGTTGATGGGGTTGAAGGGGGAAGTAGCGGAAGGTTTAAATTACRTGTTGCT

s00259g14131.CDS AGATCCTTAGTTGATGGGGTTGAAGGGGGAAGTAGCGGAAGGTTTAAATTACGTGTTGCT

**************************************************** *******

HORAISHI AATCCTTATTCAAGAATGACTTCTAAAGATGTGGAAGAAGCCTCAAACATGTTTAGACTG

Caprifig6085 AATCCTTATTCAAGAATGACTTCTAAAGATGTGGAAGAAGCCTCAAACATGTTTAGACTG

s00259g14131.CDS AATCCTTATTCAAGAATGACTTCTAAAGATGTGGAAGAAGCCTCAAACATGTTTAGACTG

************************************************************

12223-><-11879

HORAISHI TTCATCTCTAGTCTATTTCTCAGCGTTCCTGTCTTTCTCATACGAGTAGTTTGTCCACAC

Caprifig6085 TTCATCTCTAGTCTATTTCTCAGCGTTCCTGTCTTTCTCATACGAGTAGTTTGTCCACAC

s00259g14131.CDS TTCATCTCTAGTCTATTTCTCAGCGTTCCTGTCTTTCTCATACGAGTAGTTTGTCCACAC

************************************************************

HORAISHI ATACCACTGATTTATTCCTTATTGCTTTGGCGATGTGGGCCCTTTCAAATGGGTGACTGG

Caprifig6085 ATACCACTGATTTATTCCTTATTGCTTTGGCGATGTGGGCCCTTTCAAATGGGTGACTGG

s00259g14131.CDS ATACCACTGATTTATTCCTTATTGCTTTGGCGATGTGGGCCCTTTCAAATGGGTGACTGG

************************************************************

|11760

HORAISHI TTGAAGTGGGCGTTGGTGAGTGTTGTTCAATTTGTTGTTGGAAAGCGCTTCTACATTGCT

Caprifig6085 TTGAAGTGGGCGTTGGTGAGTGTYGTTCAATTTGTTGTTGGAAAGCGCTTCTACATTGCT

s00259g14131.CDS TTGAAGTGGGCGTTGGTGAGTGTTGTTCAATTTGTTGTTGGAAAGCGCTTCTACATTGCT

*********************** ************************************

HORAISHI GCTGCAAGAGCTCTACGAAATGGTTCAACTAACATGGATGTTTTGGTTGCATTGGGGACA

Caprifig6085 GCTGCAAGAGCTCTACGAAATGGTTCAACTAACATGGATGTTTTGGTTGCATTGGGGACA

s00259g14131.CDS GCTGCAAGAGCTCTACGAAATGGTTCAACTAACATGGATGTTTTGGTTGCATTGGGGACA

************************************************************

HORAISHI TCAGCCTCCTATTTCTACTCTGTTTGTGCACTTCTATATGGTGCACTCACTGGGTTTTGG

Caprifig6085 TCAGCCTCCTATTTCTACTCTGTTTGTGCACTTCTATATGGTGCACTCACTGGGTTTTGG

s00259g14131.CDS TCAGCCTCCTATTTCTACTCTGTTTGTGCACTTCTATATGGTGCACTCACTGGGTTTTGG

************************************************************

HORAISHI TCCCCAACTTACTTTGAAACAAGTGCAATGCTGATTACATTTGTGCTGTTAGGAAAATAT

Caprifig6085 TCCCCAACTTACTTTGAAACAAGTGCAATGCTGATTACATTTGTGCTGTTAGGAAAATAT

s00259g14131.CDS TCCCCAACTTACTTTGAAACAAGTGCAATGCTGATTACATTTGTGCTGTTAGGAAAATAT

************************************************************

HORAISHI CTAGAGTGTCTTGCAAAGGGGAAAACATCTGACGCCATCAAAAAGTTGGTAGAACTTGCA

Caprifig6085 CTAGAGTGTCTTGCAAAGGGGAAAACATCTGACGCCATCAAAAAGTTGGTAGAACTTGCA

s00259g14131.CDS CTAGAGTGTCTTGCAAAGGGGAAAACATCTGACGCCATCAAAAAGTTGGTAGAACTTGCA

************************************************************

11450-><-11253

HORAISHI CCAGCAACGGCAACATTGCTTATCAAGGATAAAGATGGAAGATATCTTGGAGAAAGGGAA

Caprifig6085 CCAGCAACGGCAACATTGCTTATCAAGGATAAAGATGGAAGATATCTTGGAGAAAGGGAA

s00259g14131.CDS CCAGCAACGGCAACATTGCTTATCAAGGATAAAGATGGAAGATATCTTGGAGAAAGGGAA

************************************************************

HORAISHI ATAGATGCTCTACTAATTCAGCCCGGTGACACATTGAAAGTTTCACCGGGTGCAAAATTA

Caprifig6085 ATAGATGCTCTACTAATTCAGCCCGGTGACACATTGAAAGTTTCACCGGGTGCAAAATTA

s00259g14131.CDS ATAGATGCTCTACTAATTCAGCCCGGTGACACATTGAAAGTTTCACCGGGTGCAAAATTA

************************************************************

HORAISHI CCTGCTGATGGTGTTGTTGCTTGGGGTTCAAGTTATGTAAATGAAAGTATGGTAACTGGT

Caprifig6085 CCTGCTGATGGTGTTGTTGCTTGGGGTTCAAGTTATGTAAATGAAAGTATGGTAACTGGT

s00259g14131.CDS CCTGCTGATGGTGTTGTTGCTTGGGGTTCAAGTTATGTAAATGAAAGTATGGTAACTGGT

************************************************************

HORAISHI GAATCTGTACCCATTTCGAAGGAGGTCGGTTCATCAGTAATTGGAGGTACAATAAATTTA

Caprifig6085 GAATCTGTACCCATTTCGAAGGAGGTCGGTTCATCAGTAATTGGAGGTACAATAAATTTA

s00259g14131.CDS GAATCTGTACCCATTTCGAAGGAGGTCGGTTCATCAGTAATTGGAGGTACAATAAATTTA

************************************************************

HORAISHI CATGGTGCTCTACACGTACAAGCTACCAAAGTAGGTTCTGACACAGTTTTGAGCCAGATA

Caprifig6085 CATGGTGCTCTACACGTACAAGCTACCAAAGTAGGTTCTGACACAGTTTTGAGCCAGATA

s00259g14131.CDS CATGGTGCTCTACACGTACAAGCTACCAAAGTAGGTTCTGACACAGTTTTGAGCCAGATA

************************************************************

10928->

HORAISHI ATCAGTTTAGTTGAAACAGCGCAGATGTCCAAAGCTCCTATTCAGAAATTTGCTGATTTC

Caprifig6085 ATCAGTTTAGTTGAAACAGCGCAGATGTCCAAAGCTCCTATTCAGAAATTTGCTGATTTC

s00259g14131.CDS ATCAGTTTAGTTGAAACAGCGCAGATGTCCAAAGCTCCTATTCAGAAATTTGCTGATTTC

************************************************************

<-10680 10622-><

HORAISHI ATCGCTAGCATTTTTGTTCCTACAGTTGTTTTGTTGGCATTGTTGACGTTATTAGGATGG

Caprifig6085 ATCGCTAGCATTTTTGTTCCTACAGTTGTTTTGTTGGCATTGTTGACGTTATTAGGATGG

s00259g14131.CDS ATCGCTAGCATTTTTGTTCCTACAGTTGTTTTGTTGGCATTGTTGACGTTATTAGGATGG

************************************************************

-10533

HORAISHI TACGCTGCTGGAGCTCTTCGTGCTTACCCAGAAAGTTGGCTTCCAGAAAATGGAAATTAC

Caprifig6085 TACGCTGCTGGAGCTCTTCGTGCTTACCCAGAAAGTTGGCTTCCAGAAAATGGAAATTAC

s00259g14131.CDS TACGCTGCTGGAGCTCTTCGTGCTTACCCAGAAAGTTGGCTTCCAGAAAATGGAAATTAC

************************************************************

|10431

HORAISHI TTTGTTTTCGCCCTTATGTTTTCTATATCAGTTGTGGTGATCGCGTGCCCCTGTGCTCTT

Caprifig6085 TTTGTTTTCGCCCTTATGTTTTCTATATCAGTTGTGGTGATYGCGTGCCCCTGTGCTCTT

s00259g14131.CDS TTTGTTTTCGCCCTTATGTTTTCTATATCAGTTGTGGTGATCGCGTGCCCCTGTGCTCTT

***************************************** ******************

|10377

HORAISHI GGCTTGGCAACACCAACTGCGGTCATGGTTGCAACCGGAGTCGGTGCTAACAATGGTGTG

Caprifig6085 GGCTTGGCAACACCAACTGCGGTCATGGTTGCAACAGGAGTCGGTGCTAACAATGGTGTG

s00259g14131.CDS GGCTTGGCAACACCAACTGCGGTCATGGTTGCAACCGGAGTCGGTGCTAACAATGGTGTG

***********************************.************************

HORAISHI CTGATAAAAGGGGGCGATGCCTTAGAAAGGGCTCAAAAAATAAAGTATGTGATCTTTGAT

Caprifig6085 CTGATAAAAGGGGGCGATGCCTTAGAAAGGGCTCAAAAAATAAAGTATGTGATCTTTGAT

s00259g14131.CDS CTGATAAAAGGGGGCGATGCCTTAGAAAGGGCTCAAAAAATAAAGTATGTGATCTTTGAT

************************************************************

HORAISHI AAAACAGGTACCCTAACTCAGGGAAAGGCCTCGGTTACAACTACAAAAGTGTTTTCGGGA

Caprifig6085 AAAACAGGTACCCTAACTCAGGGAAAGGCCTCGGTTACAACTACAAAAGTGTTTTCGGGA

s00259g14131.CDS AAAACAGGTACCCTAACTCAGGGAAAGGCCTCGGTTACAACTACAAAAGTGTTTTCGGGA

************************************************************

10191-><-9963

HORAISHI ATGGATCGTGGAGAATTCCTCAAGTTGGTGGCTTCTGCTGAGGCTAGTAGTGAACATCCA

Caprifig6085 ATGGATCGTGGAGAATTCCTCAAGTTGGTGGCTTCTGCTGAGGCTAGTAGTGAACATCCA

s00259g14131.CDS ATGGATCGTGGAGAATTCCTCAAGTTGGTGGCTTCTGCTGAGGCTAGTAGTGAACATCCA

************************************************************

|(9900)

HORAISHI CTGGCAAAGGCAATAGTTGCATATGCCCGTCATTTTCATTTCTTTGATGATTCTGCTACC

Caprifig6085 CTGGCAAAGGCAATAGTTGCATATGCCCGTCATTTTCATTTCTTTRATGATTCTGCTACC

s00259g14131.CDS CTGGCAAAGGCAATAGTTGCATATGCCCGTCATTTTCATTTCTTTGATGATTCTGCTACC

********************************************* **************

|[9876] |(9855)

HORAISHI AAGGATGCCAAAAGCCATAGCAAAGACTCCGAAGTATCTGGTTGGCTTTTTGATGTAGCA

Caprifig6085 AAGGATGCCRAAAGCCATAGCAAAGACTCCRAAGTATCTGGTTGGCTTTTTGATGTAGCA

s00259g14131.CDS AAGGATGCCAAAAGCCATAGCAAAGACTCCGAAGTATCTGGTTGGCTTTTTGAT------

********* ******************** ***********************

|9823 9767->

HORAISHI GAATTTNCTACTCTTCCCGGCAGAGGAGTTCAATGCTTTATTAATGAAAAACAGATTTTG

Caprifig6085 GAATTTTCTACTCTTCCCGGCAGAGGAGTTCAATGCTTTATTAATGAAAAACAGATTTTG

s00259g14131.CDS ------------------------------------------------------ATTTTG

******

<-9675

HORAISHI GTTGGTAATCGCAAGTTGATGACCGAAAGTGGAATCGACATCCCTGATGATGTAGAAAAA

Caprifig6085 GTTGGTAATCGCAAGTTGATGACCGAAAGTGGAATCGACATCCCTGATGATGTAGAAAAA

s00259g14131.CDS GTTGGTAATCGCAAGTTGATGACCGAAAGTGGAATCGACATCCCTGATGATGTAGAAAAA

************************************************************

HORAISHI TTTGTTGTAGAACTCGAAGAAAGTGCGAAAACAGGCATACTTGTCGCATATAACGGTAAC

Caprifig6085 TTTGTTGTAGAACTCGAAGAAAGTGCGAAAACAGGCATACTTGTCGCATATAACGGTAAC

s00259g14131.CDS TTTGTTGTAGAACTCGAAGAAAGTGCGAAAACAGGCATACTTGTCGCATATAACGGTAAC

************************************************************

HORAISHI TTAATTGGTGTTTTGGGGGTTGCAGACCCACTGAAAAGAGAGGCAGCTGTGGTAGTTGAG

Caprifig6085 TTAATTGGTGTTTTGGGGGTTGCAGACCCACTGAAAAGAGAGGCAGCTGTGGTAGTTGAG

s00259g14131.CDS TTAATTGGTGTTTTGGGGGTTGCAGACCCACTGAAAAGAGAGGCAGCTGTGGTAGTTGAG

************************************************************

HORAISHI GGCCTGAGTAAAATGGGCATCCGACCGGTCATGGTTACTGGGGACAACTGGAGGACAGCG

Caprifig6085 GGCCTGAGTAAAATGGGCATCCGACCGGTCATGGTTACTGGGGACAACTGGAGGACAGCG

s00259g14131.CDS GGCCTGAGTAAAATGGGCATCCGACCGGTCATGGTTACTGGGGACAACTGGAGGACAGCG

************************************************************

9418-><-9249

HORAISHI CAGGCTGTTGCTAAGGAGGTTGGCATTCACGATGTAAGGGCAGAGGTAATGCCTGCAGGA

Caprifig6085 CAGGCTGTTGCTAAGGAGGTTGGCATTCACGATGTAAGGGCAGAGGTAATGCCTGCAGGA

s00259g14131.CDS CAGGCTGTTGCTAAGGAGGTTGGCATTCACGATGTAAGGGCAGAGGTAATGCCTGCAGGA

************************************************************

|9166

HORAISHI AAAGCCGATGTAGTTCGTTCGTTTCATAAGGATGGAATCACGGTTGCGATGGTGGGAGAC

Caprifig6085 AAAGCCGATGTAGTTCGTTCGTTTCATAAGGATGGAATCACRGTTGCGATGGTGGGAGAC

s00259g14131.CDS AAAGCCGATGTAGTTCGTTCGTTTCATAAGGATGGAATCACGGTTGCGATGGTGGGAGAC

***************************************** ******************

HORAISHI GGAATCAATGATTCTCCAGCTCTAGCTGCAGCAGATGTTGGCATGGCAATTGGAGCAGGG

Caprifig6085 GGAATCAATGATTCTCCAGCTCTAGCTGCAGCAGATGTTGGCATGGCAATTGGAGCAGGG

s00259g14131.CDS GGAATCAATGATTCTCCAGCTCTAGCTGCAGCAGATGTTGGCATGGCAATTGGAGCAGGG

************************************************************

HORAISHI ACGGATATTGCCATTGAAGCGGCCAACTATGTTTTGATGAGGAACAACTTGGAGGATGTG

Caprifig6085 ACGGATATTGCCATTGAAGCGGCCAACTATGTTTTGATGAGGAACAACTTGGAGGATGTG

s00259g14131.CDS ACGGATATTGCCATTGAAGCGGCCAACTATGTTTTGATGAGGAACAACTTGGAGGATGTG

************************************************************

|8998

HORAISHI ATCACCGCCATTGATCTCTCAAGAAAGACTTTCTCTCGAATCAGATTAAATTATGTGTTT

Caprifig6085 ATCACCGCCATTGATCTCTCAAGAAAGACKTTCTCTCGAATCAGATTAAATTATGTGTTT

s00259g14131.CDS ATCACCGCCATTGATCTCTCAAGAAAGACTTTCTCTCGAATCAGATTAAATTATGTGTTT

*****************************.******************************

HORAISHI GCCATGGCTTACAATGTCATAGCAATCCCTGTTGCTGCCGGAGTTTTCTTTCCGTCGTTG

Caprifig6085 GCCATGGCTTACAATGTCATAGCAATCCCTGTTGCTGCCGGAGTTTTCTTTCCGTCGTTG

s00259g14131.CDS GCCATGGCTTACAATGTCATAGCAATCCCTGTTGCTGCCGGAGTTTTCTTTCCGTCGTTG

************************************************************

HORAISHI GGGATTCAGTTGCCGCCATGGGCGGCTGGTGCATGCATGGCTTTGTCTTCTGTTAGTGTT

Caprifig6085 GGGATTCAGTTGCCGCCATGGGCGGCTGGTGCATGCATGGCTTTGTCTTCTGTTAGTGTT

s00259g14131.CDS GGGATTCAGTTGCCGCCATGGGCGGCTGGTGCATGCATGGCTTTGTCTTCTGTTAGTGTT

************************************************************

HORAISHI GTATGTTCTTCTCTATTACTTAGGAGATACAGAAAACCAAGGCTGACCACTATACTAGAA

Caprifig6085 GTATGTTCTTCTCTATTACTTAGGAGATACAGAAAACCAAGGCTGACCACTATACTAGAA

s00259g14131.CDS GTATGTTCTTCTCTATTACTTAGGAGATACAGAAAACCAAGGCTGACCACTATACTAGAA

************************************************************

8773->

HORAISHI ATAACTGTAGAATAG

Caprifig6085 ATAACTGTAGAATAG

s00259g14131.CDS ATAACTGTAGAATAG

****
